# Sequential hydrozirconation/Pd-catalyzed cross coupling of acyl chlorides towards conjugated (2*E*,4*E*)-dienones

**DOI:** 10.3762/bjoc.19.17

**Published:** 2023-02-17

**Authors:** Benedikt Kolb, Daniela Silva dos Santos, Sanja Krause, Anna Zens, Sabine Laschat

**Affiliations:** 1 Institut für Organische Chemie, Universität Stuttgart, Pfaffenwaldring 55, D-70569 Stuttgart, Germany

**Keywords:** cross coupling, hydrozirconation, palladium catalysis, Schwartz's reagent, terpenes

## Abstract

Dienones are challenging building blocks in natural product synthesis due to their high reactivity and complex synthesis. Based on previous work and own initial results, a new stereospecific sequential hydrozirconation/Pd-catalyzed acylation of enynes with acyl chlorides towards conjugated (2*E*,4*E*)-dienones is reported. We investigated a number of substrates with different alkyl and aryl substituents in the one-pot reaction and showed that regardless of the substitution pattern, the reactions lead to the stereoselective formation (≥95% (2*E*,4*E*)) of the respective dienones under mild conditions. It was found that enynes with alkyl chains gave higher yields than the corresponding aryl-substituted analogues, whereas the variation of the acyl chlorides did not affect the reaction significantly. The synthetic application is demonstrated by formation of non-natural and natural dienone-containing terpenes such as β-ionone which was available in 4 steps and 6% overall yield.

## Introduction

**Scheme 1:**
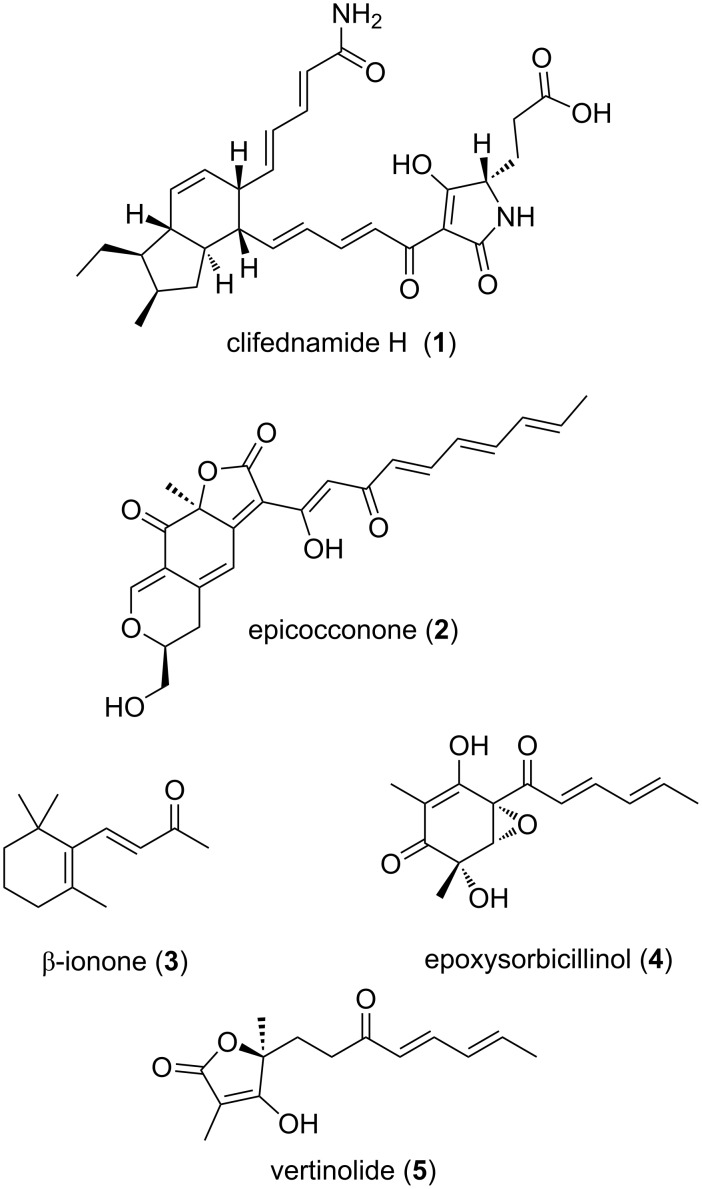
Examples of biologically active compounds with (2*Ε*,4*E*)-unsaturated ketone units.

Conjugated dienones are recurring structural motifs in natural products. Several biologically relevant compounds carry (2*Ε*,4*E*)-unsaturated ketones or the corresponding esters or amides. Selected examples are clifednamide H (**1**) which displays pronounced cytotoxicity against two human cancer cell lines [1], epicocconone (**2**), a fluorescent compound from the fungus *Epicoccum nigrum* [2], β-ionone (**3**) from many plant-derived sources [3], epoxysorbicillinol (**4**) from the saltwater culture of the fungus *Trichoderma longibrachiatum* separated from a haliclona marine sponge [4], and vertinolide (**5**) from *Verticillium intertextum* [5] (Scheme 1).

**Scheme 2:**
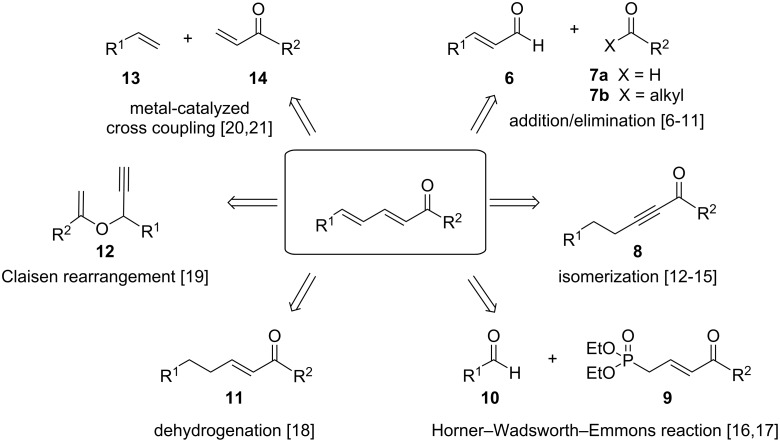
Selected examples for the synthesis of conjugated dienones from the literature [6–21].

As outlined in Scheme 2, a variety of methods has been reported for the synthesis of conjugated dienones, mostly via addition/elimination reactions such as Knoevenagel condensation or Claisen–Schmidt condensation of enals **6** with aldehydes **7a** or ketones **7b** [6–11], isomerization of alkynones **8** [12–15], Horner–Wadsworth–Emmons reaction of unsaturated phosphonates **9** and aldehydes **10** [16–17], and dehydrogenation of enones **11** [18]. Further, Claisen rearrangement of vinyl propargylic ethers **12** [19] and metal-catalyzed cross coupling of alkenes **13** and enones **14** [20–21] have been reported.

However, these reactions face multiple disadvantages such as limited substrate scope, use of hazardous solvents and harsh reaction conditions such as high temperatures or acidic/basic conditions, which might be incompatible with existing functional groups and/or the stereochemical integrity [18,22]. Especially in natural product synthesis, dienone functional groups suffer from isomerization and polymerization [23]. Therefore, a late stage introduction of dienone units is advantageous [24].

**Scheme 3:**
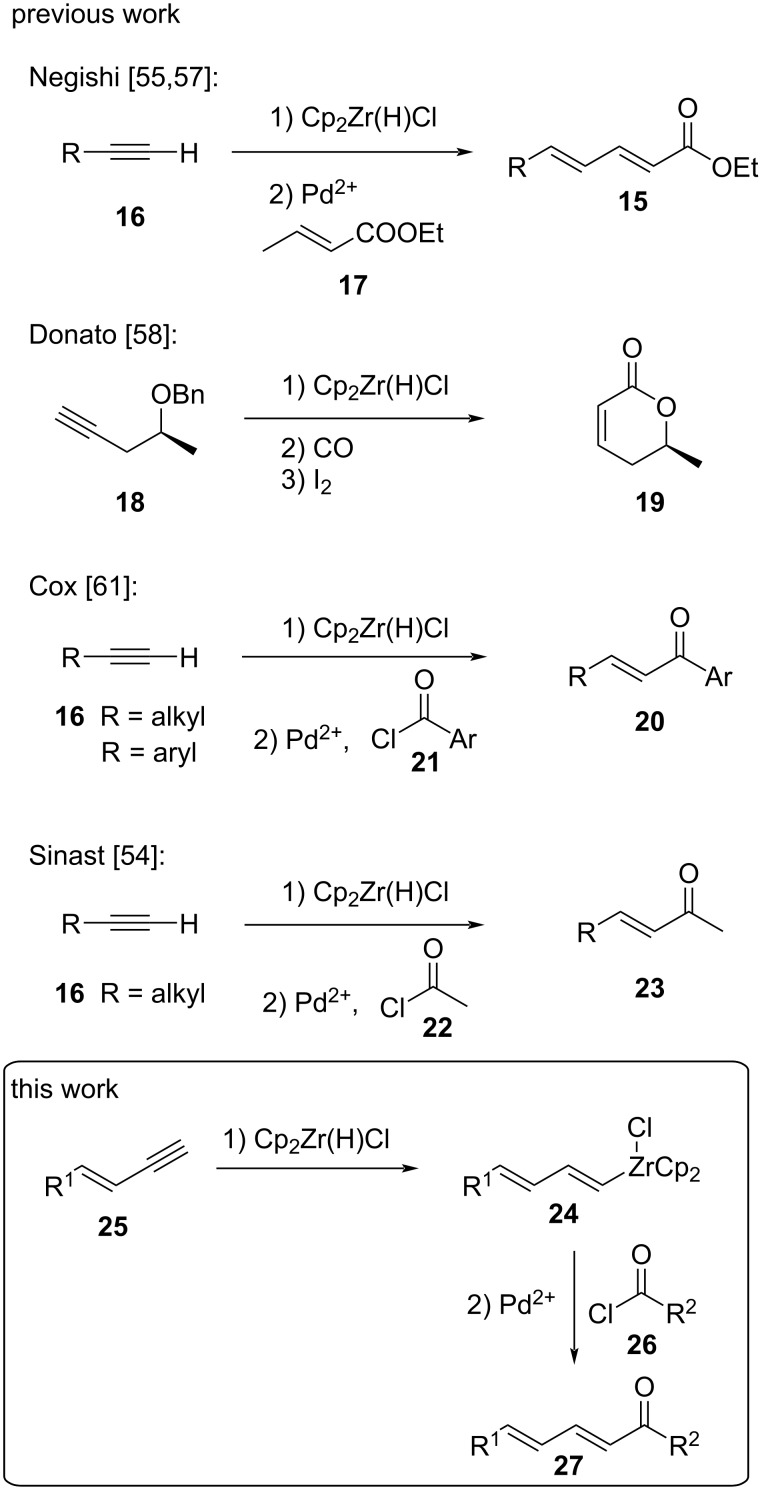
Previous work of hydrozirconations with Schwartz's reagent and our work [54–55,57–58,61–62].

Since the early work by Wailes, Schwartz and Buchwald on the Schwartz reagent Cp_2_Zr(H)Cl and its reactivity towards alkynes, alkenes, and C–X double bonds particularly hydrozirconation has gained much attention [25–30]. It has been successfully employed in methodology studies [31–40] as well as in several total syntheses of natural products [41–46]. Especially the combination of hydrozirconation and Pd or Ni-catalyzed cross coupling was elaborated by several groups (Scheme 3) [47–54].

Negishi extended Crombie’s work on dienamides and prepared dienoates **15** by hydrozirconation of terminal alkynes **16** followed by Pd-catalyzed cross coupling with enoates **17** [55–56]. A repetitive approach gave rise to oligoenoates [57]. Hydrozirconations were also combined with carbonylations to install carbonyl groups. For example, the sequential hydrozirconation/carbonylation of propargylic ethers **18** reported by Donato [58] yielded α,β-unsaturated lactones **19**. Beside the hydrozirconation/acylation sequence of nitriles utilizing acid chlorides published by Majoral/Floreancig [59–60], Cox revealed that terminal alkynes **16** could be converted to enones **20** by hydrozirconation followed by Pd-catalyzed acylation with acyl chlorides **21** [61]. The substrate scope required aryl units at either alkyne or acid chloride unit. Recently, we could extend this method to alkyl-substituted alkynes **16** and acetyl chloride (**22**), providing enone building blocks **23** for the synthesis of clifednamides [54,62]. The addition of Schwartz's reagent proceeds as a *syn*-addition affording (*E*)-alkenylzirconocenes **24** from terminal alkynes [28]. Based on these precedents from the literature, we surmised that it might be possible to establish a related approach to convert (*E*)-enynes **25** via hydrozirconation to the corresponding (*E*)-alkenylzirconocenes **24** and subsequent Pd-catalyzed acylation to conjugated (2*E*,4*E*)-dienones **27**.

## Results and Discussion

The investigation of the hydrozirconation/acylation sequence required first the synthesis of (*E*)-enynes **25** via Corey–Fuchs reaction (Table 1) [63]. Starting from different substituted, (*E*)-configurated enals **28**, the respective dibromo-olefines **29** were formed by reaction of **28** with 4 equiv of PPh_3_ and 2 equiv of CBr_4_ for 3 h. Treating **29** with 2.2 equiv of *n*-butyllithium for 1 h resulted in the desired enynes **25a**–**e** in yields up to 77%. Unfortunately, in the case of nitro-substituted enyne **25d** only 4% were isolated due to rapid decomposition and instability issues (Table 1, entry 4).

**Table 1:** Synthesis of enynes **25** via Corey–Fuchs reaction.

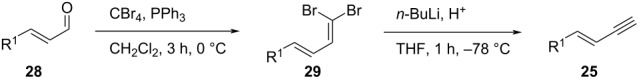			

entry	**25**	R^1^	yield **25** [%]

1	**a**	Ph	48
2	**b**	4-Me-C_6_H_4_	53
3	**c**	4-Cl-C_6_H_4_	48
4	**d**	4-NO_2_-C_6_H_4_	4
5	**e**	*n*-C_7_H_15_	77

With enynes **25a**–**e** in hand, the influence of solvent, reaction temperature, time, and Pd source on the hydrozirconation and subsequent coupling were examined by using phenylenyne **25a** and benzoyl chloride (**26a**) as benchmark substrates. The results are summarized in Table 2. For example, treatment of phenylenyne **25a** with 1.12 equiv of commercially available Schwartz reagent in THF at 50 °C for 1 h and subsequent treatment with benzoyl chloride (**26a**) in the presence of 5 mol % of Pd(PPh_3_)_2_Cl_2_ for 20 h at room temperature yielded 49% of the desired (2*E*,4*E*)-configurated dienone **27a** (Table 2, entry 1). No other stereoisomer was detected in the crude product, suggesting an all *E*-configuration ≥95% (via ^1^H NMR). Accordingly, the internal double bond in **25a** stayed unaffected whereas the triple bond, as expected, selectively formed the double bond with (*E*)-configuration. Note that a reaction temperature of 50 °C is required in the first step of the sequence to obtain rapid solubility of the Schwartz reagent in the solvent but is not necessarily required in the subsequent steps. When the reaction was carried out in benzene, CH_2_Cl_2_ or dioxane, much lower yields of 28%, 31%, and 8%, respectively, were obtained (Table 2, entries 2–4). Toluene gave the best yield with 55% (Table 2, entry 5). Therefore, further optimization steps were performed with toluene. By running the reaction at room temperature and decreased reaction times (3 h), the yield decreased to 28% (Table 2, entry 6). On increasing the reaction temperature to 50 °C instead, the product was isolated in 31% yield (Table 2, entry 7). Longer reaction times of 20 h at 50 °C led only to 8% yield (Table 2, entry 8).

**Table 2:** Hydrozirconation and Pd-catalyzed cross coupling of **25a** and **26a** with various solvents, reaction times, and temperatures.

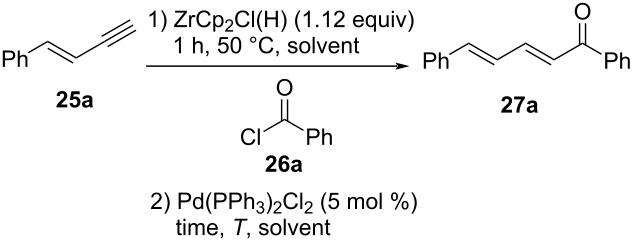				

entry	solvent	temp. [°C]	time [h]	yield **27a** [%]

1	THF	rt	20	49
2	benzene	rt	20	28
3	CH_2_Cl_2_	rt	20	31
4	dioxane	rt	20	8
5	toluene	rt	20	55
6	toluene	rt	3	28
7	toluene	50	3	31
8	toluene	50	20	8

Trapping experiments of the in situ-formed (*E*)-alkenylzirconocene **24** revealed quantitative conversion of the starting material **25a** and only traces of chain walking (less than 4%) (for details see Supporting Information File 1, chapter 2.1). To optimize the second step of the reaction sequence, several Pd complexes were tested in the reaction of **25a** with **26a**. However, the yields of the dienone **27a** decreased considerably, when (Ph_3_P)_2_PdCl_2_ was replaced by other Pd complexes (Table 3, entries 2–5). In particular, (AntPhos)_2_Pd(dba) and (XPhos)_2_Pd(dba) were catalytically inactive (Table 3, entries 6 and 7). Furthermore, in situ-formed Schwartz reagent was found to be less effective as compared to the use of isolated Cp_2_Zr(H)Cl and thus further experiments in this direction were abandoned.

**Table 3:** Hydrozirconation and Pd-catalyzed cross coupling of **25a** and **26a** by using different Pd catalysts.

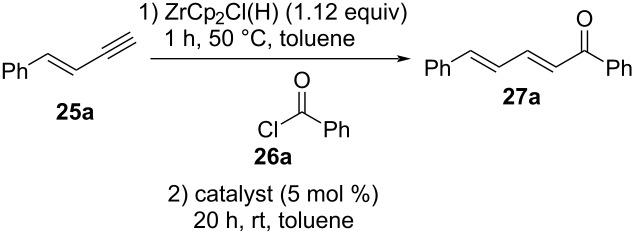		

entry	catalyst	yield **27a** [%]

1	(PPh_3_)_2_PdCl_2_	55
2	(PhCN)_2_PdCl_2_	25
3	(dppp)PdCl_2_	14
4	(dppf)PdCl_2_	11
5	Pd(PPh_3_)_4_	7
6	(AntPhos)_2_Pd(dba)	traces
7	(XPhos)_2_Pd(dba)	–

In order to explore the substrate scope of the sequential reaction, different enynes **25** and acid chlorides **26** were studied (Table 4). Fortunately, all desired products **27** were formed in ≥95% (2*E*,4*E*)-configuration (via ^1^H NMR). First, phenylenyne **25a** (R^1^ = Ph) was chosen as the starting material and reacted with different acid chlorides **26a**–**s** (Table 4). While 4-methyl-benzoic acid chloride (**26b**) behaved similarly to benzoyl chloride (**26a**) giving **27ab** in a slightly higher yield of 57% (Table 4, entry 2), the yield of **27ac** dropped to only 17% upon use of the corresponding 2-methylbenzoyl chloride (**26c**) (Table 4, entry 3). By attaching further electron-donating or electron-withdrawing groups at the benzoyl chloride **26** (Table 4, entries 4–8), the yields of the desired products decreased to 10–30% compared to **27ab**. Benzoyl chlorides carrying multiple substituents, such as 2,4,6-trichlorobenzoyl chloride (**26i**), 3,5-dinitrobenzoyl chloride (**26j**), 3,4,5-trimethoxybenzoyl chloride (**26k**), and pentafluorobenzoyl chloride (**26l**) were also tested, but did not give any trace of the respective dienone **27** (Table 4, entries 9–12). Consequently, the hydrozirconation and Pd-catalyzed cross coupling is rather sensitive towards both electron-donating and electron-withdrawing substituents at the benzoyl moiety of **25**. The analysis of crude ^1^H NMR spectra of **27** indicated decomposition and side product formation (for details see Supporting Information File 1, chapter 2.2). Unfortunately, due to the poor amount of side products, these compounds could not be isolated by chromatography and HPLC purification steps. However, GC–MS analysis of the crude product of one exemplary dienone **27ac** (with only 17% yield) indicated only decomposition in the reaction sequence.

**Table 4:** Hydrozirconation and Pd-catalyzed cross coupling of enyne **25** and acyl-chlorides **26**.

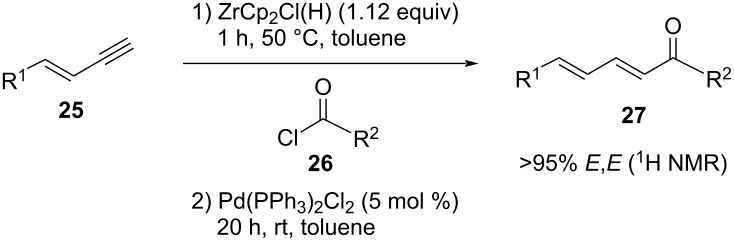						

entry	R^1^	**25**	R^2^	**26**	yield **27** [%]	**27**

1	Ph	**a**	Ph	**a**	55	**aa**
2	Ph	**a**	4-Me-C_6_H_4_	**b**	57	**ab**
3	Ph	**a**	2-Me-C_6_H_4_	**c**	17	**ac**
4	Ph	**a**	4-F-C_6_H_4_	**d**	19	**ad**
5	Ph	**a**	4-Cl-C_6_H_4_	**e**	10	**ae**
6	Ph	**a**	4-Br-C_6_H_4_	**f**	20	**af**
7	Ph	**a**	4-NO_2_-C_6_H_4_	**g**	30	**ag**
8	Ph	**a**	4-MeO-C_6_H_4_	**h**	14	**ah**
9	Ph	**a**	2,4,6-Cl_3_-C_6_H_2_	**i**	–	**ai**
10	Ph	**a**	2,4-NO_2_-C_6_H_3_	**j**	–	**aj**
11	Ph	**a**	3,4,5-OMe-C_6_H_2_	**k**	–	**ak**
12	Ph	**a**	C_6_F_5_	**l**	–	**al**

13	Ph	**a**	C_2_H_2_-Ph	**m**	12	**am**
14	Ph	**a**	C_8_H_17_	**n**	43	**an**
15	Ph	**a**	C_2_H_2_-Me	**o**	40	**ao**
16	Ph	**a**	*t*-Bu	**p**	32	**ap**

17	Ph	**a**	Me	**22**	32	**aq**
18	Ph	**a**	OEt	**r**	22	**ar**
19	Ph	**a**	CH_2_Cl	**s**	23	**as**

20	4-Me-C_6_H_4_	**b**	Ph	**a**	9	**ba**
21	4-Cl-C_6_H_4_	**c**	Ph	**a**	17	**ca**
22	4-NO_2_-C_6_H_4_	**d**	Ph	**a**	10	**da**
23	*n*-C_7_H_15_	**e**	Ph	**a**	34	**ea**
24	*n*-C_7_H_15_	**e**	Me	**22**	65	**eq**

Further, phenylenyne **25a** was treated with different conjugated and aliphatic acyl chlorides **26m**–**p** (Table 4, entries 13–16). While the reaction with cinnamoyl chloride (**26m**) gave only 12% of desired dienone **27am** (Table 4, entry 13), the yield increased up to 40% by using crotonoyl chloride **26o** instead (Table 4, entry 15). In general, by using aliphatic acyl chlorides, the yields increased, e.g., nonanoyl chloride (**26n**) yielded 43% of the desired product **27an** (Table 4, entry 14) and even the sterically hindered pivaloyl chloride (**26p**) gave 32% of dienone **27ap** (Table 4, entry 16). Due to these promising results, we tested the combination of phenylenyne **25a** with acetyl chloride (**22**), which however provided 32% of the corresponding dienone **27aq** (Table 4, entry 17). The reaction with ethyl chloroformate (**26r**) and chloroacetyl chloride (**26s**) gave decreased yields with 22% and 23% (Table 4, entries 18 and 19).

Next, the variation of enynes **25** was investigated (Table 4, entries 20–24). The sequence again showed its sensitivity towards both electron-donating and electron-withdrawing substituents on the phenyl group of the enyne **25**. The reaction of 4-methylphenylenyne **25b** with benzoyl chloride (**26a**, Table 4, entry 20) led to the desired product **27ba** in only 9% yield. With 4-chlorophenylenyne **25c** as well as 4-nitrophenylenyne **25d**, 17% and 10% yield of product were obtained, respectively (Table 4, entries 21 and 22). In contrast, aliphatic enynes showed promising results. Due to their high volatility, we limited the following experiments to enyne **25e** with a long alkyl chain. Reaction of **25e** with benzoyl chloride (**26a**) gave 34% of the dienone **27ea** (Table 4, entry 23), whereas the reaction with acetyl chloride (**22**) gave 65% yield of the desired product **27eq** (Table 4, entry 24).

**Scheme 4:**
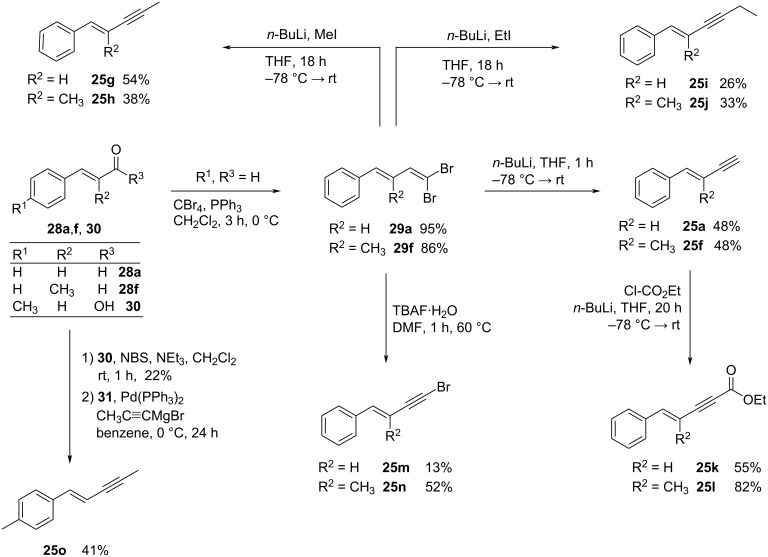
Synthesis of substituted enynes **25f–o** via Corey–Fuchs reaction and Hunsdiecker reaction.

In the following series of experiments, substituted enynes **25f–o** were employed, which were synthesized beforehand via Corey–Fuchs reaction [63] starting from (*E*)-configurated enals **28a** and **28f** forming **29a** and **29f** in 95% and 86% yield, respectively (Scheme 4). The dibromo-olefines **29a** and **29f** were then each treated with 2.2 equiv of *n*-butyllithium for 1 h to form enynes **25a** and **25f** in 48% yield. Whereas treating **29a** and **29f** with 2.2 equiv of *n*-butyllithium and 5 equiv of alkyl iodide led to isolation of the alkyl-substituted compounds **25g–j** with up to 54% yield. The reaction of **29a** and **29f** with TBAF·H_2_O gave bromoenynes **25m** in 13% and **25n** in 52% yield. Deprotonation of **25a** and **25f** with *n*-butyllithium and reaction with ethyl chloroformate yielded **25k** and **25l** in 55% and 82% yield, respectively.

Furthermore, methylated enyne **25o** was obtained via Hunsdiecker reaction [64] with subsequent Pd-catalyzed Kumada coupling [65]. Therefore, 4-methylcinnamic acid (**30**) was treated with triethylamine and NBS first. After isolating the respective bromide **31** in 22% yield, it was subsequently coupled with ethynylmagnesium bromide to form enyne **25o** in 41% yield.

With the substituted enynes **25f**–**o** in hand, the hydrozirconation and cross coupling was investigated. Therefore, 4-phenyl-3-methylenyne **25f** reacted with benzoyl chloride (**26a**) smoothly to the dienone **27fa** in 55% yield (Table 5, entry 1). In agreement with the previous observations, methyl substituents at the aryl moiety and/or the alkyne terminus compromised the yield (Table 5, entries 3 and 10). Furthermore, dienones **27g**,**i–n** with bromo-, ethyl-, and ethoxycarbonyl substituents were not accessible through this approach.

**Table 5:** Hydrozirconation and Pd-catalyzed cross coupling of substituted enynes **25f–o** and acyl chloride **26a**.

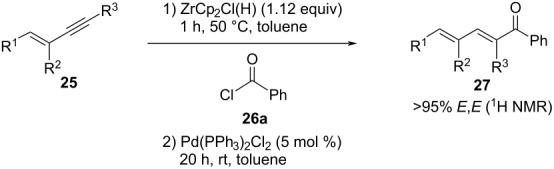						

entry	**25**	R^1^	R^2^	R^3^	**27**	yield [%]

1	**f**	Ph	Me	H	**fa**	55
2	**g**	Ph	H	Me	**ga**	0
3	**h**	Ph	Me	Me	**ha**	17
4	**i**	Ph	H	Et	**ia**	0
5	**j**	Ph	Me	Et	**ja**	0
6	**k**	Ph	H	COOEt	**ka**	0
7	**l**	Ph	Me	COOEt	**la**	0
8	**m**	Ph	H	Br	**ma**	0
9	**n**	Ph	Me	Br	**na**	0
10	**o**	4-Me-C_6_H_4_	H	Me	**oa**	10

**Scheme 5:**
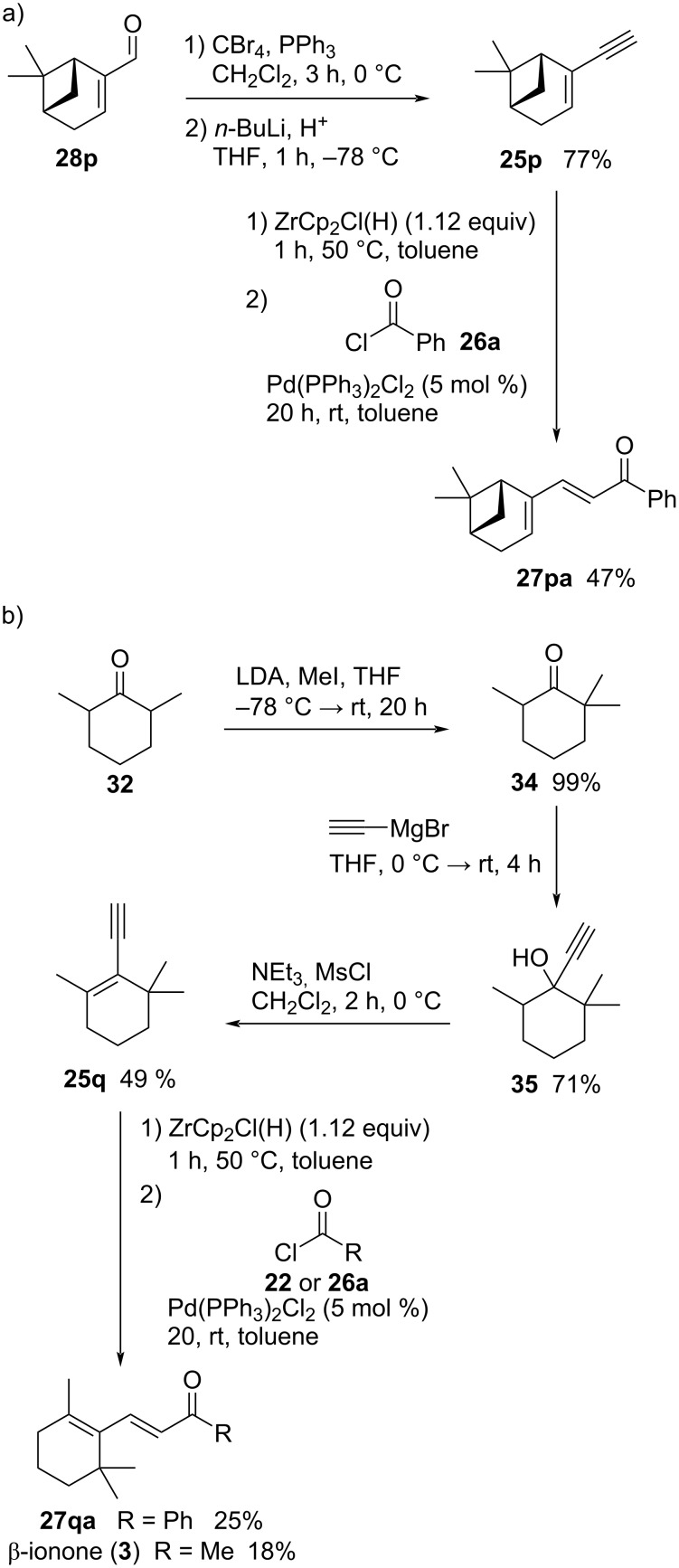
Synthesis of non-natural (a) and natural (b) dienone-containing terpenes: synthesis of β-ionone (**3**).

As the sequential hydrozirconation/Pd-catalyzed acylation worked reasonably well for aliphatic substrates, we surmised that terpene-derived enynes might be suitable starting materials for natural product synthesis. For this purpose, two terpene enynes **25p** and **25q** were synthesized and investigated in the hydrozirconation/acylation sequence (Scheme 5).

Following the Corey–Fuchs procedure as described above, enyne **25p** was synthesized using (−)-myrtenal (**28p**) as starting material in 77% yield. The synthesis of enyne **25q** started from 2,6-dimethylcyclohexanone (**32**), which was deprotonated with LDA at −78 °C in THF and subsequently methylated to give **34** in 99%, followed by treatment with alkynyl Grignard reagent to give the tertiary alcohol **35** in 71% yield. Final elimination with MsCl and NEt_3_ yielded the desired enyne **25q** (49%). When terpene enynes **25p** and **25q** were submitted to the hydrozirconation/Pd-catalyzed acylation sequence with benzoyl chloride (**26a**), the corresponding dienones **27pa** and **27qa** could be isolated in 47% and 25% yield, respectively. The reaction of **25q** with acetyl chloride (**22**) gave the fragrant β*-*ionone (**3**) in 18% yield.

## Conclusion

Based on initial results by Cox on the sequential hydrozirconation/Pd-catalyzed acylation of alkynes with acyl chlorides, the scope was extended towards a novel stereospecific dienone synthesis starting from enynes **25** and acyl chlorides **26**. Our results revealed that the reaction sequence formed selectively (2*E*,4*E*)-dienones **27** (≥95% (2*E*,4*E*)) under mild conditions, as the acetylene moiety in substrates **25** only reacted to the (*E*)-olefin while the internal double bond stayed unaffected. Compared to the reaction with benzoyl chloride (**26a**), which led to the desired dienone **27aa** in 55% yield, aliphatic or conjugated acyl chlorides did not affect the reaction with phenylenyne **25a** significantly. However, in case of substituted aromatic acyl chlorides, both electron-donating and electron-withdrawing substituents at the aryl unit, decreased the yield remarkably up to 10%. The same effect was observed in the reaction of substituted phenylenynes **25b–d** with benzoyl chloride (**26a**). The best results were obtained in the reaction of aliphatic enyne **25e** with benzoyl chloride (**26a**) and acetyl chloride (**22**) in yields of 34% and 65%, respectively. Methyl substitution on the alkene functionality of enyne **25** did not affect the yield, however, methyl substitution at the alkyne terminus significantly decreased the yield. Finally, non-natural and natural dienone-containing terpenes were synthesized such as β-ionone (**3**), which was available in 4 steps (6% overall yield). Thereby, the synthetic utility was demonstrated by a late-stage introduction of the dienone unit by a hydrozirconation/acylation sequence.

## Supporting Information

File 1 Experimental part.
