# Supplementary material for: Sequential hydrozirconation/Pd-catalyzed cross coupling of acyl chlorides towards conjugated (2E,4E)-dienones
Source: Beilstein J Org Chem. 2023 Feb 17;19:176–85. doi: 10.3762/bjoc.19.17 (PMC9940601; doi:10.3762/bjoc.19.17)
Supplement: File 1 — Experimental part. [file Beilstein_J_Org_Chem-19-176-s001.pdf]

BEILSTEIN JOURNAL OF ORGANIC CHEMISTRY

Supporting Information

for

Sequential hydrozirconation/Pd-catalyzed cross coupling of acyl chlorides towards conjugated (2*E*,4*E*)-dienones

Benedikt Kolb, Daniela Silva dos Santos, Sanja Krause, Anna Zens and Sabine Laschat

Beilstein J. Org. Chem. **2023,** *19,* 176–185. doi:10.3762/bjoc.19.17

Experimental part

License and Terms: This is a supporting information file under the terms of the Creative Commons Attribution License (https://creativecommons.org/licenses/by/4.0). Please note that the reuse, redistribution and reproduction in particular requires that the author(s) and source are credited and that individual graphics may be subject to special legal provisions.
The license is subject to the *Beilstein Journal of Organic Chemistry* terms and conditions: (https://www.beilstein-journals.org/bjoc/terms)

Table of contents

1. Experimental details S2

1.1. General information S2

1.2. General procedures S2

1.3. Synthesis of **29a**, **29f**, and **31** S3

1.4. Synthesis of enynes **25** S5

1.5. Synthesis of dienones **27** S12

1.6. Synthesis of substituted dienones S21

1.7. Hydrozirconation of terpene-derived enynes S22

1.8. Synthesis of bromo diene **S1** S26

2. Control experiments S27

2.1. Trapping experiments with NBS S27

2.2. Crude NMR spectra S27

3. Analytical data S30

4. References S54

1. **Experimental details**

1.1. General information

Unless otherwise stated, reagents and solvents were used without further purification and all reactions were carried out under air. Commercially available chemicals were purchased at Sigma-Aldrich, Alfa Aesar or TCI. Hexane, CH_2Cl_2, and ethyl acetate (EtOAc) for chromatography were distilled prior to use. Air and moisture-sensitive reactions are explicitly stated and were conducted under an atmosphere of nitrogen in anhydrous solvents using Schlenk techniques. Anhydrous CH_2Cl_2 and triethylamine were obtained by distillation over CaH_2, whereas toluene was distilled over sodium. Cooling baths were prepared using acetone and liquid N_2. Thin-layer chromatography was conducted on DC Fertigfolien ALUGRAM® Xtra SIL G/UV254 from Machery-Nagel and R_f values were rounded to 0.05. Compounds were detected by fluorescence (UV light λ = 254 nm) and molybdatophosphoric acid stain (prepared from molybdatophosphoric acid (12.0 g) in EtOH (250 mL)). Flash chromatography was performed on Fluka silica gel 60 (40–63 μm) using the indicated solvents. NMR spectra were recorded on Bruker Avance 300, Ascend 400, Avance 500, and Avance 700 spectrometers in $CDCl_3$. The respective ^1H (^{13}C)-resonance frequencies were 300 MHz (75 MHz), 400 MHz (101 MHz), 500 MHz (126 MHz), and 700 MHz (176 MHz). Chemical shifts are given in ppm and $CDCl_3$ (residual) solvent peaks were used as reference for ^1H and ^{13}C spectra as internal standard (^1H NMR: $\delta(CDCl_3)$ = 7.26 ppm, ^{13}C NMR: $\delta(CDCl_3)$ = 77.1 ppm). Proton and carbon signal assignment as well as stereochemical elucidation was conducted with 2D NMR techniques (COSY, HSQC, HMBC, NOESY). The shown numbers are freely chosen for NMR assignment and do not match the IUPAC nomenclature. No assignment is given for previously reported compounds. For FTIR measurements a Bruker Vektor 22 with a MKII Golden Gate Single Reflection Diamant ATR for reduced total reflection was used. Mass spectra were obtained on Bruker Daltonics micro-TOF-Q (ESI) and Varian MAT 711 (EI) devices.

1.2. General procedures

General procedure for the synthesis of enynes (GP1)

Under an inert atmosphere (N_2), carbon tetrabromide (5.27 g, 15.9 mmol, 2.0 equiv), dissolved in dichloromethane (15 mL), was added to a solution of triphenylphosphine (8.34 g, 31.8 mmol 4 equiv) in dichloromethane (40 mL) at 0 °C. After 15 min of stirring, the respective enal (1.00 mL, 1.05 g, 7.94 mmol, 1 equiv), dissolved in dichloromethane (15 mL), was added dropwise and the reaction mixture was stirred for 3 h at the same temperature. After adding petroleum ether, the mixture was filtered through a pad of silica and washed with petroleum ether/EtOAc 20:1. The resulting dibromo compound was directly used without further purification.

The respective dibromo compound was dissolved in dry THF (20 mL) and *n*-butyllithium (6.99 mL, 1.12 g, 17.5 mmol, 2.5 M in hexane, 2.2 equiv) was added dropwise at −78 °C. After stirring for 1 h, the mixture was warmed to room temperature, quenched with sat. NH_4Cl (50 mL), and extracted with EtOAc (3 × 50 mL). The organic phase was washed with sat. NaCl (50 mL), dried ($MgSO_4$), and concentrated. Purification by column chromatography on silica gel yielded the desired product.

General procedure for the synthesis of alkylated enynes (GP2)

The respective dibromo compound **29** was dissolved in dry THF (20 ml) and *n*-butyllithium (1.50 mL, 3.72 mmol, 2.5 M in hexane) was added dropwise at −78 °C. After stirring for 1 h, alkyl iodide (0.40 mL, 0.88 mg, 6.19 mmol) was added slowly to the reaction mixture, stirred for 1 h at −78 °C and further for 16 h at room temperature. After quenching with sat. NH_4Cl (50 mL), the mixture was extracted with EtOAc (3 × 50 mL) and the organic phase was washed with sat. NaCl (50 mL), dried ($MgSO_4$). and concentrated. Purification by column chromatography yielded the desired product.

General procedure for the synthesis of dienones (GP3)

Under an inert atmosphere (N_2), the respective enyne (100 mg, 0.78 mmol, 1.1 equiv) was dissolved in anhydrous toluene under absence of light and Schwartz's reagent (205 mg, 0.80 mmol, 1.12 equiv) was added. After stirring for 1 h at 50 °C, the formed dark red solution showed complete conversion. Acyl chloride (89.0 µL, 99.8 mg, 0.71 mmol, 1.0 equiv) and $Pd(PPh_3)_2Cl_2$ (24.9 mg, 35.5 µmol, 0.05 equiv) was added at room temperature and the reaction mixture was stirred for 18 h, filtered through a pad of SiO_2, and washed with EtOAc. After removing the solvent, the mixture was purified by column chromatography on silica gel.

1.3. Synthesis of 29a, 29f, and 31

(*E*)-(4,4-Dibromobuta-1,3-dien-1-yl)benzene (29a)

Under an inert atmosphere (N_2), carbon tetrabromide (5.27 g, 15.9 mmol), dissolved in dichloromethane (15 mL), was added to a solution of triphenylphosphine (8.34 g, 31.8 mmol) in dichloromethane (60 mL) at 0 °C. After 15 min of stirring, (*E*)-cinnamaldehyde (**28a**, 1.00 mL, 1.05 g, 7.94 mmol), dissolved in dichloromethane (15 mL), was added dropwise and the reaction mixture was stirred for 3 h at the same temperature. After adding petroleum ether, the mixture was filtered through a pad of silica and washed with petroleum ether/EtOAc 20:1. Purification by column chromatography on silica gel (petroleum ether) afforded (*E*)-(4,4-dibromobuta-1,3-dien-1-yl)benzene (**29a**, 2.17 g, 7.53 mmol, 95%) as colorless solid.

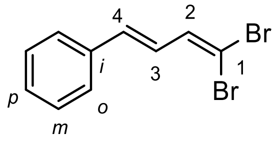

$C_{10}H_8Br_2$
287.98

29a

R_f 0.54 (petroleum ether); ^{1}H NMR (500 MHz, $CDCl_3$): δ = 6.72 (d, J = 15.7 Hz, 1H, 4-H), 6.75 - 6.84 (m, 1H, 3-H), 7.10 (d, J = 9.8 Hz, 1H, 2-H), 7.27 - 7.37 (m, 3H, *m*-H, *p*-H), 7.43 - 7.48 (m, 2H, *o*-H) ppm.; ^{13}C NMR (126 MHz, $CDCl_3$): δ = 91.4 (C-1), 125.4 (C-3), 127.0 (C-*o*), 128.8 (C-*p*), 129.0 (C-*m*), 135.8 (C-4), 136.5 (C-*i*), 137.3 (C-2) ppm. Spectroscopic data are in agreement with literature [1].

(*E*)-(4,4-Dibromo-2-methylbuta-1,3-dien-1-yl)benzene (29f)

Under an inert atmosphere (N_2), carbon tetrabromide (4.75 g, 14.3 mmol), dissolved in dichloromethane (15 mL), was added to a solution of triphenylphosphine (7.51 g, 28.6 mmol) in dichloromethane (60 mL) at 0 °C. After 15 min of stirring, *trans*-*p*-methyl

cinnamaldehyde (**28f**, 1.00 mL, 1.05 g, 7.16 mmol), dissolved in dichloromethane (15 mL), was added dropwise and the reaction mixture was stirred for 3 h at the same temperature. After adding petroleum ether, the mixture was filtered through a pad of silica and washed with petroleum ether/EtOAc 20:1. Purification by column chromatography on silica gel (petroleum ether) afforded (*E*)-(4,4-dibromo-2-methylbuta-1,3-dien-1-yl)benzene (**29f**, 1.85 g, 6.13 mmol, 86%) as yellow oil.

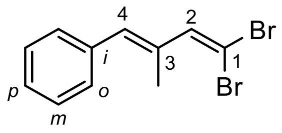

$C_{11}H_{10}Br_2$

302.01

29f

R_f 0.77 (petroleum ether); ^{1}H NMR (700 MHz, $CDCl_3$): δ = 2.15 (s, 3H, Me), 6.68 (s, 1H, 4-H), 7.14 (s, 1H, 2-H), 7.27 - 7.34 (m, 3H, *m*-H, *p*-H), 7.38 (t, *J* = 7.8 Hz, 2H, *o*-H) ppm.; ^{13}C NMR (176 MHz, $CDCl_3$): δ = 17.3 (Me), 87.7 (C-4), 127.4 (C-*p*), 128.4 (C-*m*), 129.3 (C-*o*), 133.7 (C-*i*), 134.0 (C-3), 136.9 (C-2), 141.3 (C-1) ppm.; FT-IR (ATR): $\tilde{\nu}$ = 3055 (w), 3022 (w), 2922 (w), 2855 (w), 1950 (w), 1597 (w), 1573 (w), 1492 (w), 1443 (w), 1366 (w), 1288 (w), 1196 (w), 1157 (w), 1075 (w), 1020 (w), 1003 (w), 921 (m), 890 (m), 835 (m), 790 (s), 742 (s), 696 (s), 646 (w), 620 (w), 603 (w), 577 (w), 511 (w), 467 (w), 408 (w) cm^{-1}.; GC-MS (EI): *m / z* for $C_{11}H_{10}Br_2$ calcd.: 302 [$M^{+\bullet}$], found: 302.; HRMS (EI): *m / z* for $C_{11}H_{10}Br_2$ calcd.: 301.9149 [$M^{+\bullet}$], found: 301.9129.

(*E*)-1-Bromo-*p*-methylstyrene (31)

Triethylamine (0.04 mL, 0.03 mg 0.31 mmol) was added to a suspension of *p*-methylcinnamic acid (**30**, 1.00 g, 6.17 mmol) in CH_2Cl_2 (25 mL) and stirred for 5 min at room temperature. *N*-bromosuccinimide (1.32 g, 7.40 mmol) was added and the reaction mixture was stirred for 1 h until no more carbon dioxide was formed. The suspension was filtered through a pad of silica and washed with petroleum ether/EtOAc 100:1 (200 mL). Purification via column chromatography (petroleum ether/EtOAc 100:1) yielded (*E*)-1-bromo-*p*-methylstyrene (**31**, 270 mg, 1.37 mmol, 22%) as colorless solid.

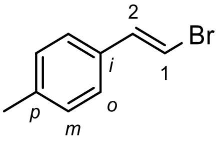

C_9H_9Br

197.08

31

R_f 0.71 (petroleum ether/EtOAc 100:1).; ^{1}H NMR (400 MHz, $CDCl_3$): δ = 2.33 (s, 3H, Me), 6.70 (d, *J* = 14.0 Hz, 1H, 2-H), 7.07 (d, *J* = 14.0 Hz, 1H, 1-H), 7.13 (d, *J* = 7.9 Hz, 2H, *m*-H), 7.19 (d, *J* = 7.9 Hz, 2H, *o*-H) ppm.; ^{13}C NMR (75 MHz, $CDCl_3$): δ = 21.4 (Me), 105.6 (C-1), 126.2 (C-*o*), 129.6 (C-*m*), 133.3 (C-*i*), 137.2 (C-2), 138.4 (C-*p*) ppm. Spectroscopic data are in agreement with literature [2].

1.4. Synthesis of enynes 25

(*E*)-But-1-en-3-yn-1-ylbenzene (25a)

According to GP1, cinnamaldehyde (**28a**, 1.00 mL, 1.05 mg, 7.94 mmol) was treated with carbon tetrabromide (5.27 g, 15.9 mmol) and triphenylphosphine (8.34 g, 15.9 mmol) in dichloromethane (70 mL) at 0 °C. The dibromo compound was dissolved in dry THF (30 mL) and *n*-butyllithium (6.99 mL, 1.12 g, 17.5 mmol, 2.5 M in hexane) was added at −78 °C. Purification by column chromatography on silica gel (petroleum ether) afforded **25a** (489 mg, 3.82 mmol, 48%) as yellow oil.

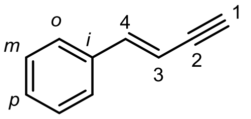

$C_{10}H_8$
128.17

25a

R_f 0.42 (petroleum ether); ^{1}H NMR (300 MHz, $CDCl_3$): δ = 3.06 (d, *J* = 2.4 Hz, 1H, 1-H), 6.14 (dd, *J* = 16.3 Hz, 2.4 Hz, 1H, 3-H), 7.05 (d, *J* = 16.3 Hz, 1H, 4-H), 7.29 - 7.42 (m, 5H, *o*-H, *m*-H, *p*-H) ppm.; ^{13}C NMR (75 MHz, $CDCl_3$): *δ* = 79.3 (C-1), 83.0 (C-2), 107.2 (C-3), 126.5 (C-*o*), 128.9 (C-*m*), 129.1 (C-*p*), 136.0 (C-*i*), 143.3 (C-4) ppm. Spectroscopic data are in agreement with literature [3].

(*E*)-1-(But-1-en-3-yn-1-yl)-4-methylbenzene (25b)

According to GP1, 4-methylcinnamaldehyde (**28b**, 500 mg, 3.42 mmol) was treated with carbon tetrabromide (2.27 g, 6.84 mmol) and triphenylphosphine (3.59 g, 13.9 mmol) in dichloromethane (40 mL) at 0 °C. The dibromo compound was dissolved in dry THF (20 mL) and *n*-butyllithium (2.48 mL, 398 mg, 6.21 mmol, 2.5 M in hexane) was added at −78 °C. Purification by column chromatography on silica gel (petroleum ether) afforded **25b** (257 mg, 1.81 mmol, 53%) as yellow oil.

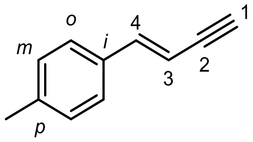

$C_{11}H_{10}$
142.20

25b

R_f 0.37 (petroleum ether); ^{1}H NMR (500 MHz, $CDCl_3$): *δ* = 2.35 (s, 3H, Me), 3.03 (d, *J* = 2.3 Hz, 1H, 1-H), 6.08 (dd, *J* = 16.4 Hz, 2.3 Hz, 1H, 3-H), 7.02 (d, *J* = 16.3 Hz, 1H, 4-H), 7.14 (d, *J* = 7.9 Hz, 2H, *o*-H), 7.27 - 7.31 (m, 2H, *m*-H) ppm.; ^{13}C NMR (126 MHz, $CDCl_3$): *δ* = 21.3 (Me), 78.8 (C-1), 83.1 (C-2), 105.9 (C-3), 126.3 (C-*m*), 129.5 (C-*o*), 133.1 (C-4), 139.1 (C-*i*), 143.1 (C-*p*) ppm. Spectroscopic data are in agreement with literature [4].

(*E*)-1-(But-1-en-3-yn-1-yl)-4-chlorobenzene (25c)

According to GP1, 4-chlorocinnamaldehyde (**28c**, 300 mg, 1.80 mmol) was treated with carbon tetrabromide (1.19 g, 3.60 mmol) and triphenylphosphine (1.89 g, 7.20 mmol) in dichloromethane (40 mL) at 0 °C. The dibromo compound was dissolved in dry THF (20 mL) and *n*-butyllithium (1.58 mL, 254 mg, 3.96 mmol, 2.5 M in hexane) was added at −78 °C.

Purification by column chromatography on silica gel (petroleum ether/EtOAc 40:1) afforded **25c** (140 mg, 48%, 0.86 mmol) as yellow solid.

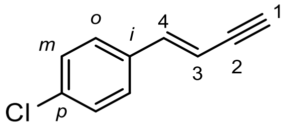

$C_{10}H_7Cl$
162.62

25c

R_f 0.62 (petroleum ether/EtOAc 40:1); ^{1}H NMR (400 MHz, $CDCl_3$): δ = 3.07 (dd, J = 2.3 Hz, 0.7 Hz, 1H, 1-H), 6.10 (dd, J = 16.3 Hz, 2.4 Hz, 1H, 3-H), 6.99 (dd, J = 16.4 Hz, 0.7 Hz, 1H, 4-H), 7.31 (m, 4H, *o*-H, *m*-H) ppm.; ^{13}C NMR (101 MHz, $CDCl_3$) δ = 79.8 (C-1), 82.6 (C-2), 107.7 (C-3), 127.5 (C-*o*), 129.0 (C-*m*), 134.4 (C-*p*), 134.7 (C-*i*), 141.8 (C-4) ppm. Spectroscopic data are in agreement with literature [4].

(*E*)-1-(But-1-en-3-yn-1-yl)-4-nitrobenzene (25d)

According to GP1, 4-nitrocinnamaldehyde (**28d**, 560 mg, 3.16 mmol) was treated with carbon tetrabromide (2.10 g, 6.32 mmol), triphenylphosphine (3.32 g, 9.71 mmol) in dichloromethane (40 mL) at 0 °C. The dibromo compound was dissolved in dry THF (20 mL) and *n*-butyllithium (2.78 mL, 446 mg, 5.34 mmol, 2.5 M in hexane) was added at −78 °C. Purification by column chromatography on silica gel (petroleum ether/EtOAc 20:1) afforded **25d** (21.0 mg, 0.12 mmol, 4%) as yellow oil.

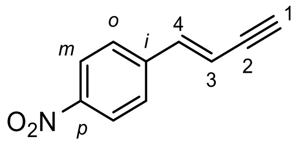

$C_{10}H_7NO_2$
173.17

25d

R_f 0.36 (petroleum ether/EtOAc 20:1); ^{1}H NMR (500 MHz, $CDCl_3$) δ = 3.14 (d, J = 2.3 Hz, 1H, 1-H), 6.22 (dd, J = 16.4 Hz, 2.4 Hz, 1H, 3-H), 7.00 (d, J = 16.3 Hz, 1H, 4-H), 7.43 - 7.49 (m, 2H, *o*-H), 8.10 - 8.16 (m, 2H, *m*-H) ppm.; ^{13}C NMR (126 MHz, $CDCl_3$): δ = 81.9 (C-1), 82.0 (C-2), 111.9 (C-3), 124.2 (C-*m*), 126.9 (C-*o*), 140.5 (C-4), 142.0 (C-*i*), 147.7 (C-*p*) ppm.; GC-MS (EI): m/z for $C_{10}H_7NO_2$ [M]: 173.0.; HRMS (EI): m/z for $C_{10}H_7NO_2$ calcd.: 173.0471 [M], found: 173.0473.; FT-IR (ATR): $\tilde{\nu}$ = 3310 (w), 3254 (s), 2954 (m), 2924 (m), 2856 (m), 1594 (s), 1505 (vs), 1343 (vs), 1319 (s), 1105 (m), 968 (m), 949 (s), 865 (m), 827 (s), 746 (s), 687 (s), 654 (s), 535 (m), 511 (s) cm^{-1}. Spectroscopic data are in agreement with literature [5]

(*E*)-Undec-3-en-1-yne (25e)

According to GP1 dec-2-enal (**28e**, 595 µL, 500 mg, 3.24 mmol) was treated with carbon tetrabromide (2.15 g, 6.48 mmol) and triphenylphosphine (3.40 g, 13.0 mmol) in dichloromethane (60 mL) at 0 °C. The dibromo compound was dissolved in dry THF (20 mL) and *n*-butyllithium (2.85 mL, 457 mg, 7.13 mmol, 2.5 M in hexane) was added at −78 °C. Purification by column chromatography on silica gel (petroleum ether) afforded **25e** (376 mg, 2.50 mmol, 77%) as colorless oil.

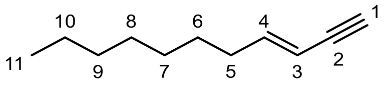

$C_{11}H_{18}$
150.27

25e

R_f 0.71 (petroleum ether); ^{1}H NMR (500 MHz, $CDCl_3$): δ = 0.88 (t, J = 6.9 Hz, 3H, 11-H), 1.20 - 1.34 (m, 8H, 6-H, 7-H, 8-H, 9-H), 1.34 - 1.45 (m, 2H, 5-H), 2.10 (qd, J = 7.2 Hz, 1.6 Hz, 2H, 10-H), 2.77 (d, J = 2.2 Hz, 1H, 1-H), 5.45 (dq, J = 16.0 Hz, 1.8 Hz, 1H, 4-H), 6.25 (dt, J = 15.9 Hz, 7.0 Hz, 1H, 3-H) ppm.; ^{13}C NMR (126 MHz, $CDCl_3$): δ = 14.1 (C-11), 22.7 (C-9), 28.6 (C-8), 29.1(C-5), 29.1 (C-7), 31.8 (C-6), 33.0 (C-10), 75.5 (C-1), 82.6(C-2), 108.4 (C-4), 147.1 (C-3) ppm.; GC-MS (EI): m/z for $C_{11}H_{17}^+$ $[M]^+$: 149.1.; HRMS (ESI): m/z for $C_{11}H_{17}^+$ calcd.: 149.1325 $[M]^+$, found: 149.1326.; FT-IR (ATR): $\tilde{\nu}$ = 3314 (m), 3023 (m), 2957 (vs), 2924 (s), 2855 (s), 2163 (w), 2106 (w), 1630 (w), 1465 (m), 1378 (w), 1198 (w), 1034 (w), 956 (vs), 723 (w), 633 (vs), 602 (vs), 417 (w) cm^{-1}.

(*E*)-(2-Methylbut-1-en-3-yn-1-yl)benzene (25f)

According to GP1, *trans*-α-methylcinnamaldehyde (**28f**, 1.00 mL, 1.05 g, 7.16 mmol) was treated with carbon tetrabromide (4.75 g, 14.3 mmol) and triphenylphosphine (7.51 g, 28.6 mmol) in dichloromethane (60 mL) at 0 °C. The dibromo compound was dissolved in dry THF (20 mL) and *n*-butyllithium (3.70 mL, 9.25 mmol, 2.5 M in hexane) was added at −78 °C. Purification by column chromatography on silica gel (petroleum ether) afforded **25f** (288 mg, 2.03 mmol, 48%) as yellow oil.

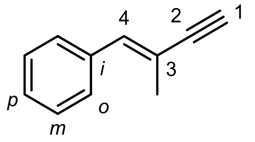

$C_{11}H_{10}$

142.20

25f

R_f 0.63 (petroleum ether); ^{1}H NMR (400 MHz, $CDCl_3$): δ = 2.10 (s, 3H, Me), 2.99 (s, 1H, 1-H), 6.95 (s, 1H, 4-H), 7.25 - 7.34 (m, 3H, *m*-H, *p*-H), 7.38 (t, J = 7.6 Hz, 2H, *o*-H) ppm.; ^{13}C NMR (101 MHz, $CDCl_3$): δ = 19.1 (Me), 76.0 (C-1), 87.6 (C-2), 119.0 (C-3), 127.5 (C-*p*), 128.4 (C-*m*), 129.1 (C-*o*), 136.6 (C-*i*), 137.6 (C-4) ppm. Spectroscopic data are in agreement with literature [6].

(*E*)-Pent-1-en-3-yn-1-ylbenzene (25g)

According to GP2, dibromo compound **29a** (1.15 g, 3.98 mmol) was dissolved in dry THF (20 mL) and *n*-butyllithium (3.50 mL, 8.76 mmol, 2.5 M in hexane) was added at −78 °C. After 1 h, iodomethane (1.20 mL, 2.82 mg, 19.9 mmol) was added. Purification by column chromatography on silica gel (petroleum ether) afforded **25g** (307 mg, 2.16 mmol, 54%) as yellow oil.

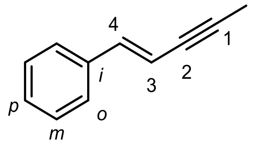

$C_{11}H_{10}$

142.20

25g

R_f 0.37 (petroleum ether); ^{1}H NMR (700 MHz, $CDCl_3$): δ = 2.01 (d, J = 2.4 Hz, 3H, 1-Me), 6.13 (dq, J = 16.2 Hz, 2.4 Hz, 1H, 3-H), 6.86 (d, J = 16.2 Hz, 1H, 4-H), 7.24 - 7.27 (m, 1H, *p*-H), 7.31 (t, J = 7.6 Hz, 2H, *m*-H), 7.36 (d, J = 8.4 Hz, 2H, *o*-H) ppm.; ^{13}C NMR (176 MHz, $CDCl_3$): δ = 4.7 (4-Me), 79.1 (C-2), 88.5 (C-1), 109.0 (C-3), 126.2 (C-*m*), 128.4 (C-*p*), 128.8 (C-*o*), 136.7 (C-4), 140.2 (C-*i*) ppm. Spectroscopic data are in agreement with literature [7].

(*E*)-(2-Methylpent-1-en-3-yn-1-yl)benzene (25h)

According to GP2, dibromo compound **29f** (0.37 g, 1.24 mmol) was dissolved in dry THF (20 mL) and *n*-butyllithium (1.50 mL, 3.72 mmol, 2.5 M in hexane) was added at −78 °C. After 1 h, iodomethane (0.40 mL, 0.88 mg, 6.19 mmol) was added. Purification by column chromatography on silica gel (petroleum ether) afforded **25h** (74.0 mg, 0.47 mmol, 38%) as colorless oil.

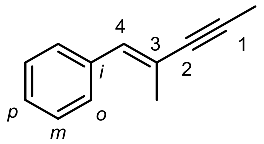

$C_{12}H_{12}$

156.23

25h

R_f 0.57 (petroleum ether); ^{1}H NMR (700 MHz, $CDCl_3$): δ = 2.00 (s, 3H, 1-Me), 2.03 (s, 3H, 3-Me), 6.75 (s, 1H, 4-H), 7.21 - 7.24 (m, 1H, *p*-H), 7.24 - 7.27 (m, 2H, *m*-H), 7.31 - 7.35 (m, 2H, *o*-H) ppm.; ^{13}C NMR (176 MHz, $CDCl_3$): δ = 4.5 (1-Me), 19.5 (3-Me), 83.6 (C-2), 84.9 (C-1), 120.6 (C-3), 127.0 (C-*p*), 128.3 (C-*m*), 129.0 (C-*o*), 134.8 (C-*i*), 137.2 (C-4) ppm. Spectroscopic data are in agreement with literature [2].

(*E*)-Hex-1-en-3-yn-1-ylbenzene (25i)

According to GP2, dibromo compound **29a** (1.68 g, 5.84 mmol) was dissolved in dry THF (20 mL) and *n*-butyllithium (5.20 mL, 12.8 mmol, 2.5 M in hexane) was added at −78 °C. After 1 h, iodoethane (2.40 mL, 4.56 mg, 29.2 mmol) was added. Purification by column chromatography on silica gel (petroleum ether) afforded **25i** (240 mg, 1.54 mmol, 26%) as yellow oil.

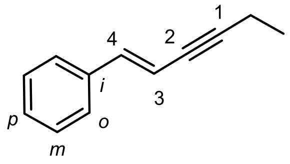

$C_{12}H_{12}$

156.23

25i

R_f 0.37 (petroleum ether); ^{1}H NMR (500 MHz, $CDCl_3$): δ = 1.21 (t, *J* = 7.5 Hz, 3H, CH_2CH_3), 2.34 - 2.43 (m, 2H, CH_2CH_3), 6.16 (dt, *J* = 16.2 Hz, 2.2 Hz, 1H, 3-H), 6.87 (d, *J* = 16.2 Hz, 1H, 4-H), 7.22 - 7.40 (m, 5H, *o*-H, *m*-H, *p*-H) ppm.; ^{13}C NMR (126 MHz, $CDCl_3$): δ = 13.5 (CH_2CH_3), 14.1 (CH_2CH_3), 79.2 (C-2), 94.4 (C-1), 109.0 (C-3), 126.2 (C-*o*), 128.4 (C-*p*), 128.8 (C-*m*), 136.7 (C-*i*), 140.2 (C-4) ppm. Spectroscopic data are in agreement with literature [8].

(*E*)-(2-Methylhex-1-en-3-yn-1-yl)benzene (25j)

According to GP2, dibromo compound **29f** (1.04 g, 3.46 mmol) was dissolved in dry THF (20 mL) and *n*-butyllithium (3.00 mL, 7.61 mmol, 2.5 M in hexane) was added at −78 °C. After 1 h, iodoethane (1.40 mL, 2.70 mg, 17.3 mmol) was added. Purification by column chromatography on silica gel (petroleum ether) afforded **25j** (191 mg, 1.12 mmol, 33%) as colorless oil.

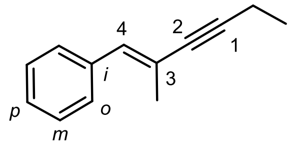

$C_{13}H_{14}$

170.26

25j

R_f 0.63 (petroleum ether); ^{1}H NMR (500 MHz, $CDCl_3$): δ = 1.21 (t, J = 7.5 Hz, 3H, CH_2CH_3), 2.04 (d, J = 1.5 Hz, 3H, Me), 2.38 (q, J = 7.5 Hz, 2H, CH_2CH_3), 6.77 (s, 1H, 4-H), 7.19 - 7.29 (m, 3H, *p*-H, *m*-H), 7.33 (t, J = 7.7 Hz, 2H, *o*-H) ppm.; ^{13}C NMR (126 MHz, $CDCl_3$): δ = 13.3 (CH_2CH_3), 14.2 (CH_2CH_3), 19.6 (Me), 83.7 (C-2), 90.8 (C-1), 120.6 (C-3), 127.0 (C-*p*), 128.3 (C-*m*), 129.0 (C-*o*), 134.8 (C-*i*), 137.3 (C-4) ppm.; FT-IR (ATR): $\tilde{\nu}$ = 3060 (w), 3022 (w), 2976 (m), 2937 (m), 2917 (w), 2877 (w), 1614 (w), 1597 (w), 1573 (w), 1492 (w), 1442 (m), 1376 (w), 1320 (m), 1241 (w), 1195 (w), 1076 (w), 1045 (w), 1031 (w), 981 (w), 919 (w), 867 (w), 758 (m), 717 (m), 696 (s), 600 (w), 508 (w), 433 (w), 416 (w) cm^{-1}.; GC-MS (EI): m/z for $C_{13}H_{14}$ calcd.: 170 [$M^{+\bullet}$], found: 170.; HRMS (EI): m/z for $C_{13}H_{14}$ calcd.: 170.1096 [$M^{+\bullet}$], found: 170.1096.

Ethyl (*E*)-5-phenylpent-4-en-2-ynoate (25k)

Under an inert atmosphere, (*E*)-(2-methylbut-1-en-3-yn-1-yl)benzene (**25a**, 0.19 g, 1.48 mmol) was dissolved in abs. THF (2 mL) at −78 °C and *n*-buthyllithium (0.62 mL, 1.55 mmol, 2.5 M in hexane) was added dropwise. After 1.5 h, ethyl chloroformate (0.17 mL, 0.19 g, 1.78 mmol) was added, and the mixture stirred for 2 h at the same temperature and further for 16 h at room temperature. The reaction mixture was poured into sat. NH_4Cl solution (30 mL), extracted with EtOAc (3 × 20 mL) and the organic phase was dried ($MgSO_4$), and concentrated. Purification by column chromatography (petroleum ether/EtOAc 20:1) yielded ethyl (*E*)-4-methyl-5-phenylpent-4-en-2-ynoate (**25k**, 163 mg, 0.81 mmol, 55%) as orange oil.

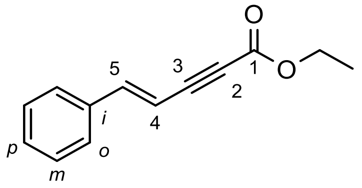

$C_{13}H_{12}O_2$

200.24

25k

R_f 0.57 (petroleum ether/EtOAc 20:1); ^{1}H NMR (700 MHz, $CDCl_3$): δ = 1.34 (t, J = 7.2 Hz, 3H, CH_2CH_3), 4.27 (q, J = 7.2 Hz, 2H, CH_2CH_3), 6.21 (d, J = 16.3 Hz, 1H, 4-H), 7.26 (d, J = 16.3 Hz, 1H, 5-H), 7.35 - 7.39 (m, 3H, *m*-H, *p*-H), 7.41 - 7.45 (m, 2H, *o*-H) ppm.; ^{13}C NMR (176 MHz, $CDCl_3$): δ = 14.2 (CH_2CH_3), 62.2 (CH_2CH_3), 82.5 (C-2), 86.0 (C-3), 104.9 (C-4), 127.1 (C-*m*), 129.1 (C-*o*), 130.2 (C-*p*), 135.2 (C-5), 147.8 (C-*i*), 154.2 (C-1) ppm. Spectroscopic data are in agreement with literature [9].

Ethyl (*E*)-4-methyl-5-phenylpent-4-en-2-ynoate (25l)

Under an inert atmosphere, (*E*)-(2-methylbut-1-en-3-yn-1-yl)benzene (**25f**, 338 mg, 2.38 mmol) was dissolved in abs. THF (5 mL) at −78 °C and *n*-buthyllithium (1.00 mL, 2.50 mmol, 2.5 M in hexane) was added dropwise. After 1.5 h, ethyl chloroformate (0.17 mL, 0.19 g, 1.78 mmol) was added, the mixture stirred for 2 h at the same temperature and for further 16 h at room temperature. The reaction mixture was poured into sat. NH_4Cl solution (40 mL), extracted with EtOAc (3 × 30 mL) and the organic phase was dried ($MgSO_4$) and

concentrated. Purification by column chromatography (petroleum ether/EtOAc 80:1) yielded ethyl (*E*)-4-methyl-5-phenylpent-4-en-2-ynoate (**25l**, 415 mg, 1.94 mmol, 82%) as orange oil.

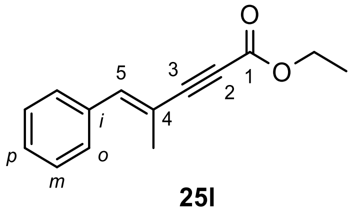

25l

$C_{14}H_{14}O_2$

214.26

R_f 0.29 (petroleum ether/EtOAc 80:1); ^{1}H NMR (500 MHz, $CDCl_3$):δ = 1.34 (t, J = 7.1 Hz, 3H, CH_2CH_3), 2.12 (d, J = 1.5 Hz, 3H, CH_3), 4.27 (q, J = 7.2 Hz, 2H, CH_2CH_3), 7.11 (d, J = 1.7 Hz, 1H, 5-H), 7.31 (d, J = 7.6 Hz, 3H, *m*-H, *p*-H), 7.34 - 7.41 (m, 2H, *o*-H) ppm.; ^{13}C NMR (126 MHz, $CDCl_3$): δ = 14.2 (CH_2CH_3), 18.4 (Me), 62. 1 (CH_2CH_3), 79.5 (C-2), 90.2 (C-3), 117.0 (C-4), 128.4 (C-*p*), 128.6 (C-*m*), 129.4 (C-*o*), 135.9 (C-*i*), 141.9 (C-1), 154.4 (C-1) ppm. Spectroscopic data are in agreement with literature [9].

(*E*)-(4-Bromobut-1-en-3-yn-1-yl)benzene (25m)

(*E*)-(4,4-Dibromobuta-1,3-dien-1-yl)benzene (**29a**, 0.92 g, 3.20 mmol) was dissolved in DMF (20 mL) and TBAF·3H_2O (5.05 g, 16.0 mmol) was added and the mixture stirred for 1 h at 60 °C. After cooling to room temperature, the reaction mixture was poured into diethyl ether (75 mL) and washed with H_2O (50 mL). The solution was extracted with diethyl ether (3 × 50 mL), washed with sat. NaCl solution, dried ($MgSO_4$), and concentrated. Purification by column chromatography (petroleum ether) yielded (*E*)-(4-bromobut-1-en-3-yn-1-yl)benzene (**25m**, 84 mg, 0.41 mmol, 13%) as orange oil.

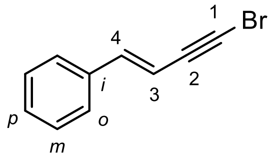

25m

$C_{10}H_7Br$

207.07

R_f 0.57 (petroleum ether); ^{1}H NMR (500 MHz, $CDCl_3$): δ = 6.12 (d, J = 16.2 Hz, 1H, 3-H), 7.01 (d, J = 16.2 Hz, 1H, 4-H), 7.27 - 7.36 (m, 3H, *m*-H, *p*-H), 7.36 - 7.39 (m, 2H, *o*-H) ppm.; ^{13}C NMR (126 MHz, $CDCl_3$): δ = 51.4 (C-1), 79.5 (C-2), 107.7 (C-3), 126.4 (C-*m*), 128.9 (C-*o*), 129.1 (C-*p*), 135.9 (C-*i*), 143.1 (C-4) ppm. Spectroscopic data are in agreement with literature [9].

(*E*)-(4-Bromo-2-methylbut-1-en-3-yn-1-yl)benzene (25n)

(*E*)-(4,4-Dibromobuta-1,3-dien-1-yl)benzene (**29f**, 1.70 g, 5.64 mmol) was dissolved in DMF (30 mL) and TBAF·3H_2O (8.89 g, 28.2 mmol) was added and the mixture stirred for 1.5 h at 60 °C. After cooling to room temperature, the reaction mixture was poured into diethyl ether (75 mL) and washed with H_2O (50 mL). The solution was extracted with diethyl ether (3 × 50 mL), washed with sat. NaCl solution, dried ($MgSO_4$), and concentrated. Purification by column chromatography (petroleum ether) yielded (*E*)-(4-bromo-2-methylbut-1-en-3-yn-1-yl)benzene (**25n**, 647 mg, 2.93 mmol, 52%) as yellow oil.

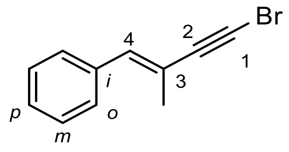

$C_{11}H_9Br$

221.10

25n

R_f 0.57 (petroleum ether); ^{1}H NMR (700 MHz, $CDCl_3$): δ = 2.07 (d, J = 1.5 Hz, 3H, Me), 6.88 (s, 1H, 4-H), 7.25 - 7.33 (m, 3H, *p*-H, *m*-H), 7.33 - 7.38 (m, 2H, *o*-H) ppm.; ^{13}C NMR (176 MHz, $CDCl_3$): δ = 19.0 (Me), 48.4 (C-4), 83.8 (C-3), 119.6 (C-2), 127.6 (C-*p*), 128.5 (C-*m*), 129.1 (C-*o*), 136.5 (C-*i*), 137.4 (C-1) ppm.; FT-IR (ATR): $\tilde{\nu}$ = 3082 (w), 3059 (w), 3024 (w), 2980 (w), 2955 (w), 2919 (w), 2857 (w), 2177 (w), 2026 (w), 1996 (w), 1598 (w), 1574 (w), 1494 (w), 1442 (m), 1378 (w), 1293 (w), 1228 (w), 1184 (w), 1157 (w), 1076 (w), 1032 (w), 1009 (w), 919 (w), 871 (w), 748 (m), 696 (s), 618 (w), 571 (w), 509 (w), 427 (w) cm^{-1}.; GC-MS (EI): m/z for $C_{11}H_9Br$ calcd.: 221 [$M^{+•}$], found: 222.; HRMS (EI): m/z for $C_{11}H_9Br$ calcd.: 219.9888 [$M^{+•}$], found: 219.9888.

(*E*)-1-Methyl-4-(pent-1-en-3-yn-1-yl)benzene (25o)

Under an inert atmosphere, (*E*)-1-bromo-*p*-methylstyrene (**31**) was dissolved in 3.20 mL benzene and tetrakis(triphenylphosphine)palladium(0) (16 mg, 0.01 mmol) was added. 1-propynylmagnesium bromide (3.20 mL, 0.23 mg, 0.5 M in THF) was added at 0 °C. The reaction mixture was stirred further for 24 h at room temperature and quenched with HCl solution (10 mL, 1 M), extracted with diethyl ether (3 × 35 mL), and dried ($MgSO_4$). Column chromatography on silica gel (petroleum ether) yielded (*E*)-1-methyl-4-(pent-1-en-3-yn-1-yl)benzene (**25o**, 86 mg, 0.55 mmol, 41%) as yellow oil.

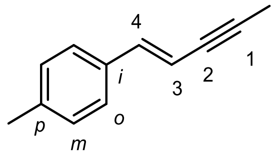

$C_{12}H_{12}$

156.23

25o

R_f 0.37 (petroleum ether); ^{1}H NMR (500 MHz, $CDCl_3$): δ = 2.01 (d, J = 2.4 Hz, 3H, 1-Me), 2.34 (s, 3H, *p*-Me), 6.08 (dq, J = 16.2 Hz, 2.4 Hz, 1H, 4-H), 6.84 (d, J = 16.2 Hz, 1H, 3-H), 7.12 (d, J = 7.9 Hz, 2H, *m*-H), 7.26 (d, J = 8.1 Hz, 2H, *o*-H) ppm.; ^{13}C NMR (126 MHz, $CDCl_3$): δ = 4.7 (*p*-Me), 21.4 (1-Me), 79.2 (C-1), 88.0 (C-2), 107.8 (C-3), 126.1 (C-*o*), 129.5 (C-*m*), 133.9 (C-*i*), 138.4 (C-4), 140.2 (C-*p*) ppm. Spectroscopic data are in agreement with literature [8].

2-Ethynyl-6,6-dimethylbicyclo[3.1.1]hept-2-ene (25p)

According to GP1, (−)-myrtenal (**28p**, 0.51 mL, 500 mg, 3.33 mmol) was treated with carbon tetrabromide (2.21 g, 6.66 mmol) and triphenylphosphine (3.46 g, 13.1 mmol) in dichloromethane (60 mL) at 0 °C. The dibromo compound dissolved in dry THF (20 mL) and *n*-butyllithium (2.93 mL, 469 mg, 7.32 mmol, 2.5 M in hexane) was added at −78 °C. Purification by column chromatography on silica gel (petroleum ether) afforded **25p** (385 mg, 2.56 mmol, 77%) as colorless oil.

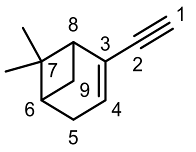

25p

$C_{11}H_{14}$
146.23

R_f 0.71 (petroleum ether); ^{1}H NMR (500 MHz, $CDCl_3$): δ = 0.89 (s, 3H, Me[1]), 1.24 (d, J = 9.1 Hz, 1H, 9-H_A), 1.29 (s, 3H, Me[2]), 2.08 - 2.13 (m, 1H, H-6), 2.25 - 2.30 (m, 1H, H-8), 2.30 - 2.37 (m, 2H, 5-H), 2.38 - 2.44 (m, 1H, 9-H_B), 2.98 (d, J = 1.0 Hz, 1H, 1-H), 6.01 - 6.12 (m, 1H, 4-H) ppm.; ^{13}C NMR (126 MHz, $CDCl_3$): δ = 21.0 (Me[1]), 26.0 (Me[2]), 31.4 (C-9), 32.0 (C-5), 38.0 (C-7), 40.2 (C-6), 46.8 (C-8), 76.7 (C-1), 84.5 (C-2), 129.1 (C-3), 132.7 (C-4) ppm.; GC-MS (EI): m/z for $C_{11}H_{14}^+$ $[M]^+$: 146.1.; HRMS (ESI): m/z for $C_{11}H_{14}^+$ calcd.: 146.1090 $[M]^+$, found: 146.1090.; FT-IR (ATR): $\tilde{\nu}$ = 3310 (s), 3031 (w), 2986 (s), 2950 (vs), 2921 (vs). 2888 (s), 2829 (m), 2092 (m), 1610 (w), 1468 (m), 1428 (m), 1383 (m), 1321 (m), 1265 (m), 1204 (m), 1115 (m), 1083 (m), 966 (m), 886 (m), 806 (s), 763 (m), 635 (s), 589 (s), 520 (m), 484 (w), 465 (w), 447 (w), 434 (w), 409 (w) cm^{-1}. Spectroscopic data are in agreement with literature [10].

1.5. Synthesis of dienones 27

(2*E*,4*E*)-1,5-Diphenylpenta-2,4-dien-1-one (27aa)

According to GP3, (*E*)-but-1-en-3-ynylbenzene (**25a**, 100 mg, 0.78 mmol) was treated with Schwartz's reagent (205 mg, 0.80 mmol), benzoyl chloride (**26a**, 89.0 µL, 99.8 mg, 0.71 mmol), and $Pd(PPh_3)_2Cl_2$ (24.9 mg, 35.5 µmol). Purification by column chromatography on silica gel (petroleum ether/EtOAc 50:1) afforded **27aa** (91 mg, 0.39 mmol, 55%) as yellow solid.

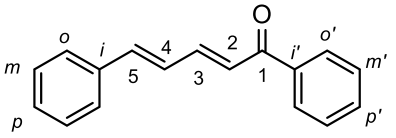

27aa

$C_{17}H_{14}O$
234.30

R_f 0.23 (petroleum ether/EtOAc 50:1); ^{1}H NMR (700 MHz, $CDCl_3$): δ = 7.00 - 7.07 (m, 2H, 4-H, 5-H), 7.10 (d, J = 15.0 Hz, 1H, 2-H), 7.30 - 7.35 (m, 1H, *p*-H), 7.36 - 7.41 (m, 2H, *m*-H), 7.47 - 7.54 (m, 4H, *o*-H, *m*'-H), 7.55 - 7.65 (m, 2H, 3-H, *p*'-H), 7.96 - 8.00 (m, 2H, *o*'-H) ppm. ; ^{13}C NMR (126 MHz, $CDCl_3$): δ = 125.6 (C-2), 127.1 (C-4), 127.5 (C-*o*), 128.6 (C-*o*'), 128.7 (C-*m*'), 129.0 (C-*m*), 129.4 (C-*p*), 132.8 (C-*p*'), 136.3 (C-*i*), 138.4 (C-*i*'), 142.1 (C-5), 145.0 (C-3), 190.7 (C-1) ppm. Spectroscopic data are in agreement with literature [11].

(2*E*,4*E*)-5-Phenyl-1-(*p*-tolyl)penta-2,4-dienone (27ab)

According to GP3, (*E*)-but-1-en-3-ynylbenzene (**25a**, 100 mg, 0.78 mmol) was treated with Schwartz's reagent (205 mg, 0.80 mmol), *p*-toluoyl chloride (**26b**, 93.8 µL, 110 mg, 0.71 mmol) and $Pd(PPh_3)_2Cl_2$ (24.9 mg, 35.5 µmol). Purification by column chromatography on silica gel (petroleum ether/EtOAc 40:1) afforded **27ab** (100 mg, 0.40 mmol, 57%) as yellow solid.

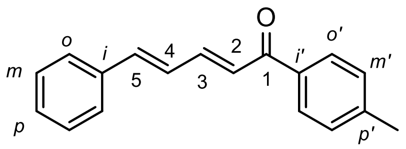

27ab

$C_{18}H_{16}O$
248.33

R_f 0.31 (petroleum ether/EtOAc 20:1); ^{1}H NMR (500 MHz, $CDCl_3$): δ = 2.43 (s, 3H, Me), 6.97 - 7.07 (m, 2H, 4-H, 5-H), 7.10 (d, J = 15.0 Hz, 1H, 2-H), 7.27 - 7.35 (m, 3H, *m*'-H, *p*-H), 7.35 - 7.41 (m, 2H, *m*-H), 7.48 - 7.53 (m, 2H, *o*-H), 7.60 (ddd, J = 15.0 Hz, 8.6 Hz, 1.7 Hz, 1H, 3-H), 7.87 - 7.93 (m, 2H, *o*'-H) ppm.; ^{13}C NMR (126 MHz, $CDCl_3$): δ = 21.7 (Me), 125.5 (C-2), 127.1 (C-4), 127.3 (C-*o*), 128.6 (C-*o*'), 128.9 (C-*m*), 129.2 (C-*p*), 129.3 (C-*m*'), 135.7 (C-*i*), 136.2 (C-*p*'), 141.6 (C-3), 143.5 (C-5), 144.4 (C-*i*'), 190.0 (C-1) ppm. Spectroscopic data are in agreement with literature [12].

(2*E*,4*E*)-5-Phenyl-1-(*o*-tolyl)penta-2,4-dienone (27ac)

According to GP3, (*E*)-but-1-en-3-ynylbenzene (**25a**, 110 mg, 0.86 mmol) was treated with Schwartz's reagent (225 mg, 0.88 mmol), *o*-toluoyl chloride **26c** (102 µL, 121 mg, 0.78 mmol) and $Pd(PPh_3)_2Cl_2$ (27.4 mg, 39.0 µmol). Purification by column chromatography on silica gel (petroleum ether/EtOAc 40:1) afforded **27ac** (32 mg, 0.13mmol, 17%) as yellow solid.

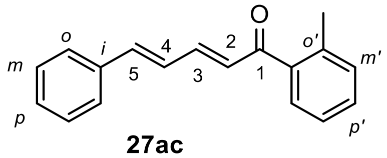

27ac

$C_{18}H_{16}O$
248.33

R_f 0.37 (petroleum ether/EtOAc 20:1); ^{1}H NMR (500 MHz, $CDCl_3$): δ = 2.48 (s, 3H, Me), 6.72 (d, J = 15.3 Hz, 1H, 2-H), 6.93 - 7.06 (m, 2H, 4-H, 5-H), 7.27 - 7.33 (m, 3H, 3-H, *m*-H_A, *p*-H), 7.34 - 7.45 (m, 4H, *m*-H_B, *m*'-H, *p*'-H), 7.51 (m, 3H, *o*-H, *o*'-H) ppm.; ^{13}C NMR (126 MHz, $CDCl_3$): δ = 20.2 (Me), 125.5 (C^1-*m*'), 126.8 (C-4), 127.3 (C-*o*), 128.0 (C^1-*o*'), 128.9 (C-*m*), 129.3 (C^2-*m*'), 130.2 (C-2), 130.4 (C-*p*'), 131.3 (C-*p*), 136.0 (C-*i*), 136.8 (C^2-*o*'), 139.2 (C-*i*'), 141.9 (C-5), 146.0 (C-3), 196.7 (C-1) ppm. Spectroscopic data are in agreement with literature [12].

(2*E*,4*E*)-1-(*p*-Fluorophenyl)-5-phenylpenta-2,4-dienone (27ad)

According to GP3, (*E*)-but-1-en-3-ynylbenzene (**25a**, 100 mg, 0.78 mmol) was treated with Schwartz's reagent (205 mg, 0.80 mmol), *p*-fluorobenzoyl chloride (**26d**, 83.8 µL, 112 mg, 0.71 mmol) and $Pd(PPh_3)_2Cl_2$ (24.9 mg, 35.5 µmol). Purification by column chromatography on silica gel (petroleum ether/EtOAc 20:1) afforded **27ad** (33 mg, 0.13 mmol, 19%) as yellow solid.

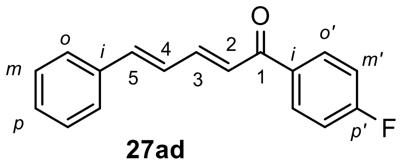

27ad

$C_{17}H_{13}FO$
252.29

R_f 0.37 (petroleum ether/EtOAc 20:1); ^{1}H NMR (500 MHz, $CDCl_3$): δ = 7.03 (d, J = 6.4 Hz, 2H, 4-H, 5-H), 7.07 (d, J = 15.0 Hz, 1H, 2-H), 7.12 - 7.20 (m, 2H, *m*'-H), 7.31 - 7.42 (m, 3H, *m*-H, *p*-H), 7.49 - 7.53 (m, 2H, *o*-H), 7.61 (ddd, J = 15.0 Hz, 6.3 Hz, 3.9 Hz, 1H, 3-H), 7.98 - 8.05 (m, 2H, *o*'-H) ppm.; ^{13}C NMR (126 MHz, $CDCl_3$): δ = 115.6 (C^1-*m*'), 115.8 (C^2-*m*'), 124.9 (C-2), 126.8 (C-4), 127.3 (C-*o*), 128.9 (C-*m*), 129.3 (C-*p*), 130.9 (C-*p*'), 131.0 (C-*o*'), 134.6 (C-*i*'), 136.1 (C-*i*), 142.2 (C-5), 145.1 (C-3), 188.8 (C-1) ppm. Spectroscopic data are in agreement with literature [12].

(2*E*,4*E*)-1-(*p*-Chlorophenyl)-5-phenylpenta-2,4-dienone (27ae)

According to GP3, (*E*)-but-1-en-3-ynylbenzene (**25a**, 100 mg, 0.78 mmol) was treated with Schwartz's reagent (205 mg, 0.80 mmol), *p*-chlorobenzoyl chloride (**26e**, 97.7 µL, 124 mg, 0.71 mmol) and $Pd(PPh_3)_2Cl_2$ (24.9 mg, 35.5 µmol). Purification by column chromatography on silica gel (petroleum ether/EtOAc 20:1) afforded **27ae** (19 mg, 70.7 µmol, 10%) as yellow solid.

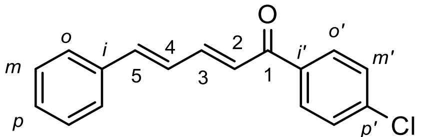

27ae

$C_{17}H_{13}ClO$
268.74

R_f 0.43 (petroleum ether/EtOAc 10:1); ^{1}H NMR (500 MHz, $CDCl_3$): δ = 7.01 - 7.07 (m, 3H, 2-H, 4-H, 5-H), 7.32 - 7.41 (m, 3H, *m*-H, *p*-H), 7.44 - 7.48 (m, 2H, *m*'-H), 7.49 - 7.54 (m, 2H, *o*-H), 7.61 (ddd, J = 15.0 Hz, 7.3 Hz, 3.1 Hz, 1H, 3-H), 7.89 - 7.95 (m, 2H, *o*'-H) ppm.; ^{13}C NMR (126 MHz, $CDCl_3$): δ = 124.8 (C-2), 126.8 (C-4), 127.4 (C-*o*), 128.9 (C-*m*'), 128.9 (C-*m*), 129.4 (C-*p*), 129.8 (C-*o*'), 136.0 (C-*p*'), 136.6 (C-*i*), 139.1 (C-*i*'), 142.4 (C-5), 145.4 (C-3), 189.2 (C-1) ppm. Spectroscopic data are in agreement with literature [13].

(2*E*,4*E*)-1-(*p*-Bromophenyl)-5-phenylpenta-2,4-dienone (27af)

According to GP3, (*E*)-but-1-en-3-ynylbenzene (**25a**, 104 mg, 0.81 mmol) was treated with Schwartz's reagent (213 mg, 0.83 mmol), *p*-bromobenzoyl chloride (**26f**, 162 mg, 0.74 mmol) and $Pd(PPh_3)_2Cl_2$ (25.9 mg, 36.9 µmol). Purification by column chromatography on silica gel (petroleum ether/EtOAc 30:1) afforded **27af** (47.0 mg, 0.15 mmol, 20%) as yellow solid.

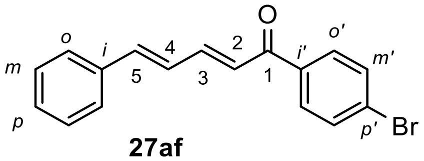

27af

$C_{17}H_{13}BrO$
313.19

R_f 0.43 (petroleum ether/EtOAc 20:1); ^{1}H NMR (400 MHz, CDCl3): δ = 6.90 - 7.02 (m, 3H, 5-H, 4-H, 2-H), 7.23 - 7.35 (m, 3H, *m*-H, *p*-H), 7.40 - 7.46 (m, 2H, *o*-H), 7.50 - 7.60 (m, 3H, 3-H, *m*'-H), 7.74 - 7.81 (m, 2H, *o*'-H) ppm.; ^{13}C NMR (176 MHz, $CDCl_3$): δ = 124.8 (C-5), 126.8 (C-4), 127.4 (C-*o*), 127.8 (C-*p*'), 128.9 (C-*m*), 129.4 (C-*p*), 129.9 (C-*o*'), 131.9 (C-*m*'),136.0 (C-*i*), 136.9 (C-*i*'), 142.5 (C-2), 145.4 (C-3), 189.4 (C-1) ppm. Spectroscopic data are in agreement with literature [13].

(2*E*,4*E*)-1-(4-Nitrophenyl)-5-phenylpenta-2,4-dienone (27ag)

According to GP3, (*E*)-but-1-en-3-ynylbenzene (**25a**, 100 mg, 0.78 mmol) was treated with Schwartz's reagent (205 mg, 0.80 mmol), 4-nitrobenzoyl chloride (**26g**, 132 mg, 0.71 mmol) and $Pd(PPh_3)_2Cl_2$ (24.9 mg, 35.5 µmol). Purification by column chromatography on silica gel (petroleum ether/EtOAc 20:1) afforded **27ag** (58 mg, 0.30 mmol, 30%) as yellow solid.

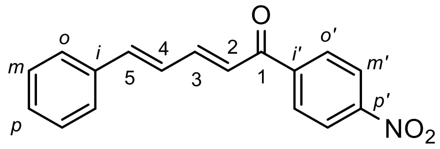

27ag

$C_{17}H_{13}NO_3$
279.30

*R*f 0.42 (petroleum ether/EtOAc 10:1); ^{1}H NMR (400 MHz, $CDCl_3$): δ = 6.99 - 7.13 (m, 3H, 2-H, 4-H, 5-H), 7.38 (dd, *J* = 9.9 Hz, 7.0 Hz, 3H, *m*-H, *p*-H), 7.52 (d, *J* = 7.3 Hz, 2H, *o*-H), 7.64 (dd, *J* = 15.0 Hz, 9.4 Hz, 1H, 3-H), 8.10 (d, *J* = 8.4 Hz, 2H, *o*'-H), 8.34 (d, *J* = 8.5 Hz, 2H, *m*'-H) ppm.; ^{13}C NMR (126 MHz, $CDCl_3$): *δ* = 123.9 (C-*m*'), 124.5 (C-4), 126.5 (C-*p*), 127.5 (C-*o*), 129.0 (C-*m*), 129.3 (C-*o*'), 129.7 (C-2), 135.8 (C-*i*), 143.1 (C-*i*'), 143.6 (C-5), 146.8 (C-3), 149.9 (C-*p*'), 188.9 (C-1) ppm. Spectroscopic data are in agreement with literature [14].

(2*E*,4*E*)-1-(*p*-Methoxyphenyl)-5-phenylpenta-2,4-dienone (27ah)

According to GP3, (*E*)-but-1-en-3-ynylbenzene (**25a**, 105 mg, 0.82 mmol) was treated with Schwartz's reagent (215 mg, 0.83 mmol), *p*-methoxybenzoyl chloride (**26h**, 127 mg, 0.75 mmol) and $Pd(PPh_3)_2Cl_2$ (26.1 mg, 37.4 mmol). Purification by column chromatography on silica gel (petroleum ether/EtOAc 30:1) afforded **27ah** (28 mg, 0.11 mmol, 14%) as yellow solid.

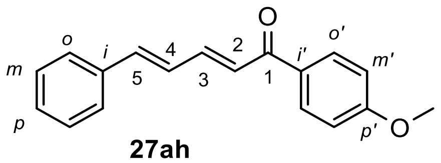

27ah

$C_{18}H_{16}O_2$
264.32

*R*f 0.26 (petroleum ether/EtOAc 20:1); ^{1}H NMR (700 MHz, $CDCl_3$) *δ* = 3.88 (s, 3H, OMe), 6.96 - 6.99 (m, 2H, *m*'-H), 6.99 - 7.05 (m, 2H, 4-H, 5-H), 7.11 (d, *J* = 15.0 Hz, 1H, 2-H), 7.32 (m, 1H, *p*-H), 7.35 - 7.40 (m, 2H, *m*-H), 7.48 - 7.52 (m, 2H, *o*-H), 7.60 (dd, *J* = 15.0 Hz, 9.7 Hz, 1H, 3-H), 7.98 - 8.03 (m, 2H, *o*'-H) ppm.; ^{13}C NMR (176 MHz, $CDCl_3$): *δ* = 55.5 (OMe), 113.8 (C-*m*'), 125.3 (C-2), 127.1 (C-5), 127.3 (C-*o*), 128.9 (C-*m*), 129.1 (C-*p*), 130.7 (C-*m*'), 131.2 (C-*i*), 136.3 (C-*i*'), 141.4 (C-4), 144.0 (C-3), 163.4 (C-*p*'), 188.7 (C-1) ppm. Spectroscopic data are in agreement with literature [12].

(2*E*,4*E*)-1-Phenyl-5-(*p*-tolyl)penta-2,4-dien-1-one (27ba)

According to GP3, (*E*)-1-(but-1-en-3-yn-1-yl)-4-methylbenzene (**25b**, 115 mg, 0.81 mmol) was treated with Schwartz's reagent (212 mg, 0.82 mmol), benzoyl chloride (**26a**, 85.4 µL, 103 mg, 0.74 mmol) and $Pd(PPh_3)_2Cl_2$ (25.8 mg, 36.8 mmol). Purification by column chromatography on silica gel (petroleum ether/EtOAc 40:1) afforded **27ba** (16 mg, 64.4 µmol, 9 %) as yellow solid.

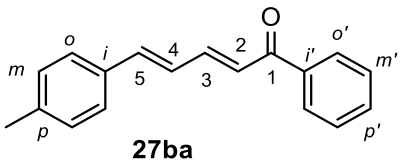

^{1}H NMR (500 MHz, $CDCl_3$): δ = 2.38 (s, 3H, Me), 6.97 - 7.02 (m, 2H, 4-H, 5-H), 7.08 (d, *J* = 14.9 Hz, 1H, 2-H), 7.19 (d, *J* = 7.9 Hz, 2H, *m*-H), 7.39 - 7.43 (m, 2H, *o*-H), 7.46 - 7.52 (m, 2H, *m*'-H), 7.54 - 7.64 (m, 2H, 3-H, *p*'-H), 7.95 - 8.01 (m, 2H, *o*'-H) ppm.; ^{13}C NMR (126 MHz, $CDCl_3$): δ = 21.5 (Me), 124.9 (C-2), 126.1 (C-4), 127.3 (C-*o*), 128.4 (C-*o*'), 128.6 (C-*m*'), 129.6 (C-*m*), 132.6 (C-*p*'), 133.4 (C-*p*), 138.4 (C-*i*), 139.6 (C-*i*'), 142.1 (C-5), 145.2 (C-3), 190.6 (C-1) ppm. Spectroscopic data are in agreement with literature [15].

(2*E*,4*E*)-5-(4-Chlorophenyl)-1-phenylpenta-2,4-dien-1-one (27ca)
According to GP3, (*E*)-1-(but-1-en-3-yn-1-yl)-4-chlorobenzene (**25c**, 100 mg, 0.62 mmol) was treated with Schwartz's reagent (162 mg, 0.63 mmol), benzoyl chloride (**26a**, 64.9 µL, 78.6 mg, 0.56 mmol) and $Pd(PPh_3)_2Cl_2$ (19.6 mg, 28.0 mmol). Purification by column chromatography on silica gel (petroleum ether/EtOAc 30:1) afforded **27ca** (25 mg, 93.0 µmol, 17%) as yellow solid.

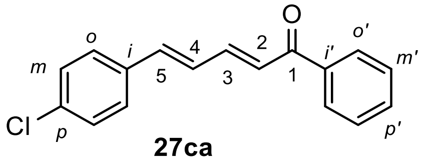

R_f 0.29 (petroleum ether/EtOAc 30:1); ^{1}H NMR (400 MHz, $CDCl_3$) δ = 6.91 - 7.04 (m, 2H, 4-H, 5-H), 7.10 (d, *J* = 15.0 Hz, 1H, 2-H), 7.32 - 7.37 (m, 2H, *m*'-H), 7.40 - 7.45 (m, 2H, *o*'-H), 7.46 - 7.52 (m, 2H, *m*-H), 7.54 - 7.62 (m, 2H, 3-H, *p*'-H), 7.95 - 8.00 (m, 2H, *o*-H) ppm; ^{13}C NMR (101 MHz, $CDCl_3$) δ = 125.9 (C-4), 127.5 (C-*m*'), 128.4 (C-2, C-*o*), 128.6 (C-*m*), 129.1 (C-*o*'), 132.7 (C-*p*), 134.6 (C-*i*), 135.0 (C-*p*'), 138.2 (C-*i*'), 140.3 (C-5), 144.3 (C-3), 190.4 (C-1) ppm. Spectroscopic data are in agreement with literature [12].

(2*E*,4*E*)-5-(4-Nitrophenyl)-1-phenylpenta-2,4-dien-1-one (27da)
According to GP3, (*E*)-1-(but-1-en-3-yn-1-yl)-4-nitrobenzene (**25d**, 71.6 mg, 0.42 mmol) was treated with Schwartz's reagent (109 mg, 0.42 mmol), benzoyl chloride (**26a**, 43.9 µL, 53.1 mg, 0.38 mmol) and $Pd(PPh_3)_2Cl_2$ (13.3 mg, 18.9 mmol). Despite repeated purification via column chromatography on silica gel (petroleum ether/EtOAc 10:1) and HPLC (petroleum ether/EtOAc 98:2, Orbit 100 Sil, 5 µm), **27da** (10 mg, 35.8 µmol, 10 %) was isolated only in 63% purity.

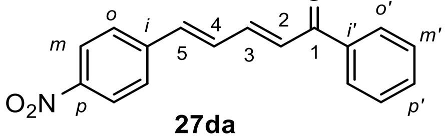

R_f 0.34 (petroleum ether/EtOAc 5:1); ^{1}H NMR (500 MHz, $CDCl_3$) δ = 7.04 (d, *J* = 15.6 Hz, 1H, 3-H), 7.12 - 7.19 (m, 1H, 5-H), 7.21 (d, *J* = 15.0 Hz, 1H, 4-H), 7.51 (dd, *J* = 8.4 Hz, 7.0 Hz, 2H, *o*'-H), 7.56 - 7.65 (m, 4H, *m*'-H, *p*'-H, 2-H), 7.95 - 8.01 (m, 2H, *o*-H), 8.21 - 8.25 (m, 2H,

m-H) ppm.; ^{13}C NMR (126 MHz, $CDCl_3$): δ = 124.2 (C-*m*), 127.7 (C-*m*'), 127.8 (C-*p*), 128.5 (C-*o*), 128.7 (C-*o*'), 131.1 (C-5), 133.1 (C-4), 137.8 (C-*i*), 138.5 (C-3), 142.3 (C-*i*'), 143.2 (C-2), 147.7 (C-*p*'), 190.1 (C-1) ppm. Spectroscopic data are in agreement with literature [16].

(1*E*,4*E*,6*E*)-1,7-Diphenylhepta-1,4,6-trien-3-one (27am)
According to GP3, (*E*)-but-1-en-3-ynylbenzene (**25a**, 101 mg, 0.79 mmol) was treated with Schwartz's reagent (207 mg, 0.80 mmol), cinnamoyl chloride (**26m**, 68.6 µL, 74.9 mg, 0.71 mmol) and $Pd(PPh_3)_2Cl_2$ (25.1 mg, 35.8 mmol). Purification by column chromatography on silica gel (petroleum ether/EtOAc 20:1) afforded **27am** (21 mg, 80.7 mmol, 12%) as yellow solid.

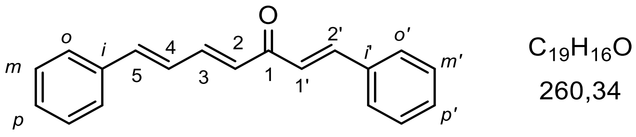

27am

R_f 0.20 (petroleum ether/EtOAc 20:1); ^{1}H NMR (700 MHz, $CDCl_3$): δ = 6.64 (d, J = 15.1 Hz, 1H, 2-H), 6.95 - 7.05 (m, 3H, 1'-H, 4-H, 5-H), 7.31 - 7.35 (m, 1H, *p*-H), 7.35 - 7.43 (m, 5H, *m*-H, *m*'-H, *p*'-H), 7.48 - 7.56 (m, 3H, 3-H, *o*-H), 7.61 (dd, J = 6.3 Hz, 2.5 Hz, 2H, *o*'-H), 7.70 (d, J = 15.9 Hz, 1H, 2'-H) ppm.; ^{13}C NMR (176 MHz, $CDCl_3$): δ = 125.5 (C-1'), 127.0 (C-4), 127.3 (C-*o*), 128.4 (C-*o*'), 128.9 (C-*m*), 129.0 (C-*2*), 129.0 (C-*m*'), 129.3 (C- *p*'), 130.5 (C-*p*), 134.9 (C-*i*'), 136.1 (C-*i*), 141.7 (C-3), 143.0 (C-2'), 143.4 (C-5), 189.0 (C-1) ppm. Spectroscopic data are in agreement with literature [13].

(1*E*,3*E*)-1-Phenyltrideca-1,3-dien-5-one (27an)
According to GP3, (*E*)-but-1-en-3-ynylbenzene (**25a**, 100 mg, 0.78 mmol) was treated with Schwartz's reagent (205 mg, 0.80 mmol), nonanoyl chloride (**26n**, 128 µL, 125 mg, 0.71 mmol) and $Pd(PPh_3)_2Cl_2$ (24.9 mg, 35.5 µmol). Purification by column chromatography on silica gel (petroleum ether/EtOAc 40:1) afforded **27an** (83.0 mg, 0.31 mmol, 43%) as yellow oil.

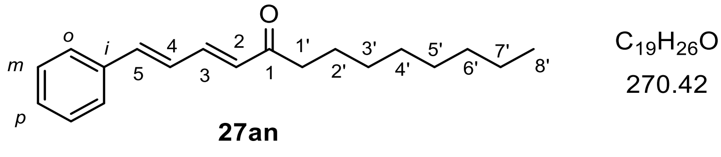

27an

R_f 0.43 (petroleum ether/EtOAc 20:1); ^{1}H NMR (700 MHz, $CDCl_3$) δ = 0.88 (t, J = 7.0 Hz, 3H, 8'-H), 1.30 (m, 10H, 3'-H, 4'-H, 5'-H, 6'-H, 7'-H), 1.64 (m, 2H, 2'-H), 2.59 (t, J = 7.5 Hz, 2H, 1'-H), 6.29 (d, J = 15.4 Hz, 1H, 5-H), 6.86 - 6.91 (m, 1H, 4-H), 6.95 (d, J = 15.6 Hz, 1H, 2-H), 7.30 - 7.34 (m, 2H, 3-H, *p*-H), 7.34 - 7.38 (m, 2H, *m*-H), 7.46 - 7.49 (m, 2H, *o*-H) ppm.; ^{13}C NMR (176 MHz, $CDCl_3$): δ = 14.1 (C-8'), 22.7 (C-7'), 24.5 (C-6'), 29.2 (C-5'), 29.4 (C-4'), 29.4 (C-3'), 31.9 (C-2'), 40.9 (C-1'), 126.8 (C-4), 127.2 (C-*o*), 128.9 (C-*m*), 129.2 (C-*p*), 129.7 (C-5), 136.1 (C-*i*), 141.1 (C-2), 142.4 (C-3), 200.9 (C-1) ppm.; MS (ESI): m/z for $C_{19}H_{26}OH^+$ calcd.: 271.21 $[M+H]^+$, found: 271.21.; m/z for $C_{19}H_{26}ONa^+$ calcd.: 293.19 $[M+Na]^+$, found: 293.19.; HRMS (ESI): m/z for $C_{19}H_{26}ONa^+$ calcd.: 293.1878 $[M+Na]^+$, found: 293.1876.; FT-IR (ATR): $\tilde{\nu}$ = 3061 (w), 3024 (w), 2999 (w), 2954 (s), 2922 (vs), 2851 (s), 1681 (vs), 1611 (m), 1590 (s), 1467 (m), 1449 (m), 1405 (w), 1334 (w), 1314 (w), 1294 (w), 1259 (w), 1223

(w), 1129 (w), 1087 (m), 1053 (w), 1034 (w), 1015 (s), 962 (w), 883 (w), 819 (w), 743 (m), 725 (w), 688 (m), 504 (w) cm^{-1}.

(2*E*,5*E*,7*E*)-8-Phenylocta-2,5,7-trien-4-one (27ao)

According to GP3, (*E*)-but-1-en-3-ynylbenzene (**25a**, 101 mg, 0.79 mmol) was treated with Schwartz's reagent (207 mg, 0.80 mmol), 4-methoxybenzoyl chloride (**26o**, 68.6 µL, 74.9 mg, 0.71 mmol) and $Pd(PPh_3)_2Cl_2$ (25.1 mg, 35.8 mmol). Purification by column chromatography on silica gel (petroleum ether/EtOAc 20:1) afforded **27ao** (40 mg, 202 mmol, 40%) as yellow solid.

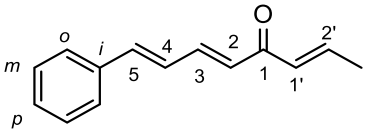

27ao

$C_{14}H_{14}O$
198.26

R_f 0.20 (petroleum ether/EtOAc 20:1); ^{1}H NMR (700 MHz, $CDCl_3$) δ = 1.95 (dd, J = 6.8 Hz, 1.7 Hz, 3H, Me), 6.38 - 6.42 (m, 1H, 1'-H), 6.51 (d, J = 15.2 Hz, 1H, 2-H), 6.90 - 7.00 (m, 3H, 2'-H, 4-H, 5-H), 7.30 - 7.33 (m, 1H, *p*-H), 7.34 - 7.39 (m, 2H, *m*-H), 7.43 (dd, J = 15.2 Hz, 10.2 Hz, 1H, 3-H), 7.46 - 7.49 (m, 2H, *o*-H) ppm.; ^{13}C NMR (176 MHz, $CDCl_3$) δ = 18.5 (Me), 127.0 C-2'), 127.2 (C-*o*), 128.3 (C-2), 128.9 (C-*m*), 129.1 (C-*p*), 130.9 (C-1'), 136.2 (C-*i*), 141.3 (C-4), 143.0 (C-5), 143.1 (C-3), 189.2 (C-1) ppm. Spectroscopic data are in agreement with literature [17].

(4*E*,6*E*)-2,2-Dimethyl-7-phenylhepta-4,6-dien-3-one (27ap)

According to GP3, (*E*)-but-1-en-3-ynylbenzene (**25a**, 100 mg, 0.78 mmol) was treated with Schwartz's reagent (205 mg, 0.80 mmol), pivaloyl chloride (**26p**, 87.4 µL, 85.5 mg, 0.71 mmol) and $Pd(PPh_3)_2Cl_2$ (24.9 mg, 35.5 µmol). Purification by column chromatography on silica gel (petroleum ether/EtOAc 60:1) afforded **27ap** (48 mg, 0.22 mmol, 32%) as yellow solid.

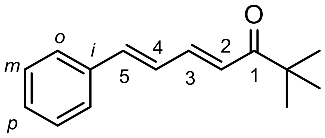

27ap

$C_{15}H_{18}O$
214.31

R_f 0.49 (petroleum ether/EtOAc 20:1); ^{1}H NMR (500 MHz, $CDCl_3$): δ = 1.20 (s, 9H, *t*-Bu), 6.68 (d, J = 15.0 Hz, 1H, 2-H), 6.87 - 6.98 (m, 2H, 4-H, 5-H), 7.27 - 7.33 (m, 1H, *p*-H), 7.33 - 7.39 (m, 2H, *m*-H), 7.43 - 7.50 (m, 3H, *o*-H, 3-H) ppm.; ^{13}C NMR (126 MHz, $CDCl_3$): δ = 26.4 ($C(CH_3)_3$), 43.1 ($C(CH_3)_3$), 124.3 (C-2), 126.9 (C-4), 127.2 (C-*o*), 128.8 (C-*m*), 129.0 (C-*p*), 136.3 (C-*i*), 141.1 (C-5), 142.9 (C-3), 204.5 (C-1) ppm. Spectroscopic data are in agreement with literature [13].

(2*E*,4*E*)-1-Phenyldodeca-2,4-dien-1-one (27ea)

According to GP3, (*E*)-undec-3-en-1-yne (**25e**, 100 mg, 0.67 mmol) was treated with Schwartz's reagent (175 mg, 0.68 mmol), benzoyl chloride (**26a**, 70.3 µL, 85.0 mg, 0.61 mmol) and $Pd(PPh_3)_2Cl_2$ (21.2 mg, 30.3 µmol). Purification by column chromatography on silica gel (petroleum ether/EtOAc 30:1) afforded **27ea** (54 mg, 0.21 mmol, 34%) as colorless oil.

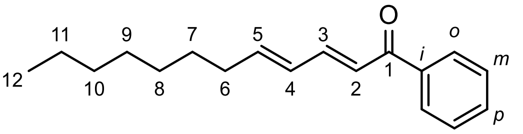

$C_{18}H_{24}O$
256.39

27ea

R_f 0.49 (petroleum ether/EtOAc 60:1); ^{1}H NMR (700 MHz, $CDCl_3$): δ = 0.89 (t, J = 7.0 Hz, 3H, 12-H), 1.30 (m, 8H, 8-H, 9-H, 10-H, 11-H), 1.45 (p, J = 7.2 Hz, 2H, 7-H), 2.21 (q, J = 7.2 Hz, 2H, 6-H), 6.26 (dt, J = 15. Hz, 6.7 Hz, 1H, 5-H), 6.32 (dd, J = 15.2 Hz, 10.6 Hz, 1H, 4-H), 6.88 (d, J = 15.0 Hz, 1H,3-H), 7.41 (dd, J = 15.1 Hz, 10.6 Hz, 1H, 2-H), 7.47 (t, J = 7.7 Hz, 2H, *m*-H), 7.53 - 7.57 (m, 1H, *p*-H), 7.94 (dd, J = 8.3 Hz, 1.4 Hz, 2H; *o*-H) ppm.; ^{13}C NMR (176 MHz, $CDCl_3$) δ = 14.1 (C-12), 22.7 (C-11), 28.7 (C-7), 29.1 (C-10), 29.2 (C-9), 31.8 (C-8), 33.3 (C-6), 123.5 (C-3), 128.4 (C-*o*), 128.5 (C-*m*), 129.1 (C-4), 132.5(C-*p*), 138.4 (C-*i*), 145.4 (C-5), 146.8 (C-2), 191.0 (C-1) ppm. Spectroscopic data are in agreement with literature [18].

(3*E*,5*E*)-Trideca-3,5-dien-2-one (27eq)

According to GP3, (*E*)-undec-3-en-1-yne (**25e**, 82.0 mg, 0.55 mmol) was treated with Schwartz's reagent (144 mg, 0.56 mmol), acetyl chloride (**22**, 35.6 µL, 39.2 mg, 0.50 mmol) and $Pd(PPh_3)_2Cl_2$ (17.5 mg, 25.0 µg). Purification by column chromatography on silica gel (petroleum ether/EtOAc 40:1) afforded **27eq** (65 mg, 0.34 mmol, 65%) as yellow solid.

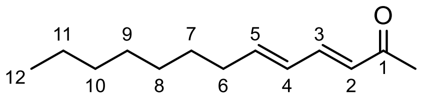

$C_{13}H_{22}O$
194.32

27eq

R_f 0.29 (petroleum ether/EtOAc 20:1); ^{1}H NMR (500 MHz, $CDCl_3$): δ = 0.88 (t, J = 6.7 Hz, 3H, 12-H), 1.22 - 1.34 (m, 8H, 8-H, 9-H, 10-H, 11-H), 1.43 (p, J = 7.5 Hz, 2H, 7-H), 2.14 - 2.22 (m, 2H, 6-H), 2.26 (d, J = 1.2 Hz, 3H, COMe), 6.05 (d, J = 15.6 Hz, 1H, 5-H), 6.13 - 6.23 (m, 2H, 2-H, 4-H), 7.09 (ddd, J = 15.6 Hz, 5.7 Hz, 3.0 Hz, 1H, 3-H) ppm.; ^{13}C NMR (176 MHz, $CDCl_3$): δ = 14.1 (C-12), 22.7 (C-8), 27.1 (COMe), 28.7 (C-11), 29.1 (C-10), 29.2 (C-9), 31.8 (C-7), 33.2 (C-6), 128.8 (C-2), 128.8 (C-3), 144.1 (C-4), 145.9 (C-5), 198.9 (C-1) ppm. Spectroscopic data are in agreement with literature [19].

(3*E*,5*E*)-6-Phenylhexa-3,5-dien-2-one (27aq)

According to GP3, (*E*)-but-1-en-3-ynylbenzene (**25a**, 100 mg, 0.78 mmol) was treated with Schwartz's reagent (205 mg, 0.80 mmol), acetyl chloride (**22**, 50.6 µL, 55.7 mg, 0.71 mmol) and $Pd(PPh_3)_2Cl_2$ (24.9 mg, 35.5 µmol). Purification by column chromatography on silica gel (petroleum ether/EtOAc 20:1) afforded **27aq** (39.0 mg, 0.23 mmol, 32%) as yellow solid.

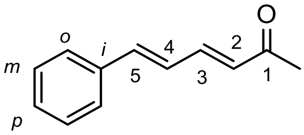

$C_{12}H_{12}O$
172.23

27aq

R_f 0.29 (petroleum ether/EtOAc 20:1); ^{1}H NMR (700 MHz, $CDCl_3$): δ = 2.35 (s, 3H, Me), 6.29 (d, J = 15.5 Hz, 1H, 5-H), 6.91 (dd, J = 15.6 Hz, 10.6 Hz, 1H, 4-H), 6.98 (d, J = 15.5 Hz, 1H, 2-H), 7.29 - 7.33 (m, 1H, 3-H), 7.33 - 7.37 (m, 1H, *p*-H), 7.39 (dd, J = 8.4 Hz, 6.8 Hz, 2H, *m*-

H), 7.48 - 7.52 (m, 2H, *o*-H) ppm.; ^{13}C NMR (176 MHz, $CDCl_3$) δ = 27.4 (Me), 126.7 (4-H), 127.3 (C-*o*), 128.9 (C-*m*), 129.3 (C-*p*), 130.5 (C-5), 136.0 (C-*i*), 141.3 (C-2), 143.5 (C-3), 198.5 (C-1) ppm. Spectroscopic data are in agreement with literature [20].

Ethyl (2*E*,4*E*)-5-phenylpenta-2,4-dienoate (27ar)

According to GP3, (*E*)-but-1-en-3-ynylbenzene (**25a**, 100 mg, 0.78 mmol) was treated with Schwartz's reagent (205 mg, 0.80 mmol), ethyl chloroformate (**26r**, 67.5 µL, 77.0 mg, 0.71 mmol) and $Pd(PPh_3)_2Cl_2$ (24.9 mg, 35.5 µmol). Purification by column chromatography on silica gel (petroleum ether/EtOAc 40:1) afforded **27ar** (32 mg, 0.16 mmol, 22%) as yellow solid.

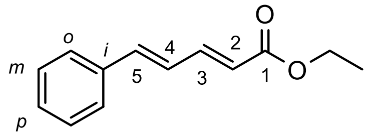

27ar

$C_{13}H_{14}O_2$
202.25

R_f 0.43 (petroleum ether/EtOAc 20:1); ^{1}H NMR (400 MHz, $CDCl_3$): δ = 1.25 (t, J = 7.1 Hz, 3H, CH_2CH_3), 4.16 (q, J = 7.1 Hz, 2H, CH_2CH_3), 5.92 (d, J = 15.3 Hz, 1H. 2-H), 6.74 - 6.88 (m, 2H, 4-H, 5-H), 7.22 - 7.32 (m, 3H, *m*-H, *p*-H), 7.33 - 7.43 (m, 3H, 3-H, *o*-H) ppm. ^{13}C NMR (126 MHz, $CDCl_3$): δ = 14.4 (CH_2CH_3), 60.4 (CH_2CH_3), 121.4 (C-2), 126.3 (C-4), 127.2 (C-*o*), 128.8 (C-*m*), 129.0 (C-*p*), 136.1 (C-*i*), 140.4 (C-3), 144.6 (C-5), 167.1 (C-1) ppm. Spectroscopic data are in agreement with literature [21].

(3*E*,5*E*)-1-Chloro-6-phenylhexa-3,5-dien-2-one (27as)

According to GP3, (*E*)-but-1-en-3-ynylbenzene (**25a**, 100 mg, 0.78 mmol) was treated with Schwartz's reagent (205 mg, 0.80 mmol), 2-cloroacetyl chloride (**26s**, 56.4 µL, 80.1 mg, 0.71 mmol) and $Pd(PPh_3)_2Cl_2$ (24.9 mg, 35.5 µmol). Purification by column chromatography on silica gel (petroleum ether/EtOAc 30:1) afforded **27as** (34 mg, 0.17 mmol, 23%) as yellow solid.

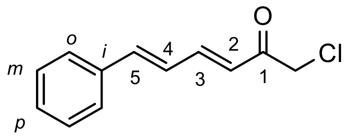

27as

$C_{12}H_{11}ClO$
206.67

R_f 0.34 (petroleum ether/EtOAc 20:1); ^{1}H NMR (500 MHz, $CDCl_3$): δ = 4.23 (s, 2H, CH_2), 6.52 (d, J = 15.3 Hz, 1H, 2-H), 6.91 (dd, J = 15.5 Hz, 10.9 Hz, 1H, 4-H), 7.02 (d, J = 15.5 Hz, 1H, 5-H), 7.31 - 7.41 (m, 3H, *m*-H, *p*-H), 7.45 - 7.52 (m, 3H, 3-H, *o*-H) ppm.; ^{13}C NMR (126 MHz, $CDCl_3$): δ = 47.4 (CH_2), 125.0 (C-2), 127.6 (C-4), 129.0 (C-*o*), 129.2 (C-*m*), 129.7 (C-*p*), 135.8 (C-*i*), 143.3 (C-5), 145.3 (C-3), 191.4 (C-1) ppm. Spectroscopic data are in agreement with literature [22].

1.6. Synthesis of substituted dienones

(2*E*,4*E*)-4-Methyl-1,5-diphenylpenta-2,4-dien-1-one (27fa)
According to GP3, (*E*)-(2-methylbut-1-en-3-yn-1-yl)benzene (**25f**, 0.10 g, 0.70 mmol) was treated with Schwartz's reagent (185 mg, 0.72 mmol), benzoyl chloride (**26a**, 0.08 mL, 90 mg, 0.64 mmol) and $Pd(PPh_3)_2Cl_2$ (22 mg, 0.03 mmol). Purification by column chromatography on silica gel (petroleum ether/EtOAc 50:1) afforded **27fa** (88 mg, 0.35 mmol, 55%) as yellow solid.

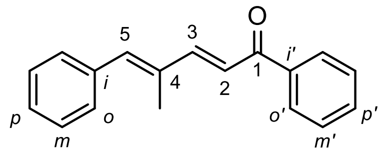

27fa

$C_{18}H_{16}O$

248.33

R_f 0.20 (petroleum ether/EtOAc 50:1); ^{1}H NMR (400 MHz, $CDCl_3$): δ = 2.17 (d, J = 1.4 Hz, 3H, Me), 6.98 (s, 1H, 5-H), 7.06 (d, J = 15.3 Hz, 1H, 2-H), 7.27 - 7.42 (m, 5H, *o*-H, *m*-H, *p*-H), 7.45 - 7.54 (m, 2H, *m*'-H), 7.54 - 7.60 (m, 1H, *p*'-H), 7.64 (d, J = 15.3 Hz, 1H, 3-H), 7.92 - 8.12 (m, 2H, *o*'-H) ppm.; ^{13}C NMR (101 MHz, $CDCl_3$): δ = 14.1 (Me), 121.7 (C-2), 128.0 (C-*p*), 128.5 (C-*o*), 128.6 (C-*o*'), 128.7 (C-*m*), 129.7 (C-*m*'), 132.7 (C-*p*'), 134.8 (C-*i*), 136.9 (C-4), 138.7 (C-*i*'), 140.7 (C-5), 150.4 (C-3), 191.0 (C-1) ppm. Spectroscopic data are in agreement with literature [16].

(2*E*,4*E*)-2,4-Dimethyl-1,5-diphenylpenta-2,4-dien-1-one (27ha)
According to GP3, (*E*)-(2-methylpent-1-en-3-yn-1-yl)benzene (**25h**, 0.10 g, 0.64 mmol) was treated with Schwartz's reagent (168 mg, 0.65 mmol), benzoyl chloride (**26a**, 0.07 mL, 82 mg, 0.58 mmol) and $Pd(PPh_3)_2Cl_2$ (20 mg, 0.03 mmol). Purification by column chromatography on silica gel (petroleum ether/EtOAc 50:1) afforded **27ha** (25 mg, 0.1 mmol, 17%) as yellow oil.

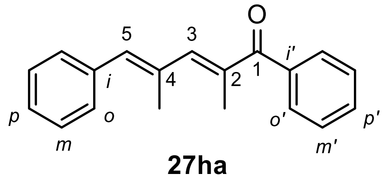

27ha

$C_{19}H_{18}O$

262.35

R_f 0.29 (petroleum ether/EtOAc 50:1); ^{1}H NMR (400 MHz, $CDCl_3$): δ = 2.14 (d, J = 1.4 Hz, 3H, 4-Me), 2.25 (d, J = 1.4 Hz, 3H, 2-Me), 6.63 (s, 1H, 5-H), 6.80 (s, 1H, 3-H), 7.30 - 7.40 (m, 5H, *o*-H, *m*-H, *p*-H), 7.43 - 7.49 (m, 2H, *m*'-H), 7.50 - 7.57 (m, 1H, *p*'-H), 7.67 - 7.73 (m, 2H, *o*'-H) ppm.; ^{13}C NMR (126 MHz, $CDCl_3$): δ = 13.3 (2-Me), 17.2 (4-Me), 126.1 (C-*p*), 126.9 (C-*m*'), 127.1 (C-*m*), 128.1 (C-*o*'), 128.2 (C-*o*), 130.2 (C-*p*'), 133.2 (C-*i*), 133.9 (C-5), 134.2 (C-2), 135.7 (C-*i*'), 137.6 (C-4), 146.1 (C-3), 198.6 (C-1) ppm.; FT-IR (ATR): $\tilde{\nu}$ = 3058 (w), 3024 (w), 2924 (w), 2854 (w), 1953 (w), 1723 (w), 1641 (m), 1597 (m), 1577 (w), 1491 (w), 1445 (m), 1380 (w), 1358 (w), 1314 (w), 1264 (m), 1177 (w), 1149 (w), 1073 (w), 1016 (m), 921 (w), 848 (w), 790 (w), 742 (m), 710 (s), 696 (s), 672 (w), 647 (w), 618 (w), 568 (w), 517 (br) cm^{-1}; MS (ESI): m/z for $C_{19}H_{18}ONa^+$ calcd.: 285.13 $[M+Na]^+$, found: 285.13.; HRMS (ESI): m/z for $C_{19}H_{18}ONa^+$ calcd.: 285.1251 $[M+Na]^+$, found: 285.1250.

(2*E*,4*E*)-2-Methyl-1-phenyl-5-(*p*-tolyl)penta-2,4-dien-1-one (27oa)
According to GP3, (*E*)-1-methyl-4-(pent-1-en-3-yn-1-yl)benzene (**25o**, 86 mg, 0.55 mmol) was treated with Schwartz's reagent (145 mg, 0.56 mmol), benzoyl chloride (**26a**, 0.06 mL, 70 mg, 0.50 mmol) and $Pd(PPh_3)_2Cl_2$ (18 mg, 0.03 mmol). Purification by column chromatography on silica gel (petroleum ether / EtOAc 50 : 1) afforded **27oa** (13 mg, 0.05 mmol, 10 %) as orange oil.

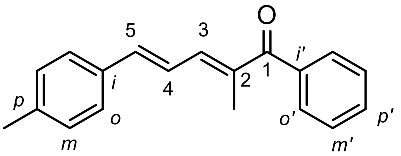

27oa

$C_{19}H_{18}O$

262.35

R_f 0.29 (petroleum ether/EtOAc 50:1); ^{1}H NMR (700 MHz, $CDCl_3$): δ = 2.18 (s, 3H, 2-Me), 2.36 (s, 3H, *p*-Me), 6.76 (d, *J* = 15.4 Hz, 1H, 5-H), 6.90 - 6.95 (m, 1H, 4-H), 7.12 - 7.18 (m, 3H, *m*-H, 3-H), 7.37 (d, *J* = 7.9 Hz, 2H, *o*-H), 7.45 (t, *J* = 7.6 Hz, 2H, *m*'-H), 7.49 - 7.55 (m, 1H, *p*'-H), 7.64 (d, *J* = 7.5 Hz, 2H, *o*'-H) ppm.; ^{13}C NMR (176 MHz, $CDCl_3$): δ = 13.0 (2-Me), 21.5 (*p*-Me), 123.4 (C-3), 127.3 (C-*o*), 128.3 (C-*m*'), 129.3 (C-*o*'), 129.7 (C-*m*), 131.3 (C-*p*'), 133.8 (C-*p*), 135.6 (C-2), 139.1 (C-*i*'), 139.4 (C-*i*), 140.1 (C-5), 143.3 (C-4), 199.0 (C-1) ppm.; FT-IR (ATR): $\tilde{\nu}$ = 3024 (br), 2923 (br), 2857 (br), 1639 (s), 1614 (s), 1577 (m), 1511 (w), 1490 (w), 1446 (m), 1383 (w), 1361 (w), 1329 (m), 1309 (m), 1281 (s), 1245 (m), 1169 (br), 1109 (w), 1074 (w), 1033 (m), 1012 (m), 970 (m), 909 (w), 806 (m), 714 (s), 648 (m), 597 (w), 520 (w), 454 (w) cm^{-1}.; MS (ESI): *m / z* for $C_{19}H_{18}ONa^+$ calcd.: 285.34 $[M+Na]^+$, found: 285.12.; HRMS (ESI): *m / z* for $C_{19}H_{18}ONa^+$ calcd.: 285.1250 $[M+Na]^+$, found: 285.1246.

1.7. Hydrozirconation of terpene-derived enynes

(*E*)-3-(6,6-Dimethylbicyclo[3.1.1]hept-2-en-2-yl)-1-phenylprop-2-en-1-one (27pa)
According to GP3, 2-ethynyl-6,6-dimethylbicyclo[3.1.1]hept-2-ene **(25p**, 105 mg, 0.72 mmol) was treated with Schwartz's reagent (189 mg, 0.73 mmol), benzoyl chloride (**26a**, 75.8 µL, 91.8 mg, 0.65 mmol) and $Pd(PPh_3)_2Cl_2$ (22.9 mg, 32.6 µmol). Purification by column chromatography on silica gel (petroleum ether/EtOAc 40:1) afforded **27pa** (77 mg, 0.31 mmol, 47%) as colorless oil.

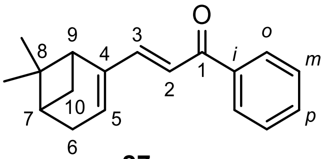

27pa

$C_{18}H_{20}O$

252.36

R_f 0.46 (petroleum ether/EtOAc 20:1); ^{1}H NMR (700 MHz, $CDCl_3$): δ = 0.80 (s, 3H, Me^1), 1.19 (d, *J* = 9.1 Hz, 1H, 10-H_A), 1.38 (s, 3H, Me^2), 2.15 - 2.21 (m, 1H, 7-H), 2.44 (dt, *J* = 20.0 Hz, 3.2 Hz, 1H, 6-H_A), 2.48 - 2.56 (m, 2H, 6-H_B, 10-H_B), 2.69 (td, *J* = 5.6 Hz, 1.5 Hz, 1H, 7-H), 6.17 (d, *J* = 3.8 Hz, 1H, 5-H), 6.84 (d, *J* = 15.4 Hz, 1H, 3-H), 7. 43 - 7.49 (m, 3H, 2-H, *m*-H), 7.51 - 7.57 (m, 1H, *p*-H), 7.94 (dd, *J* = 8.0 Hz, 1.4 Hz, 2H, *o*-H) ppm.; ^{13}C NMR (176 MHz, $CDCl_3$): δ = 20.9 (Me^1), 26.2 (Me^2), 31.2 (C-10), 32.9 (C-6), 37.9 (C-8), 40.7 (C-7), 41.4 (C-9), 118.6 (C-3), 128.4 (C-*m*), 128.5 (C-*o*), 132.4 (C-*p*), 136.4 (C-6), 138.7 (C-*i*), 145.8 (C-2),

146.4 (C-4), 191.3 (C-1) ppm.; MS (ESI): *m / z* for $C_{18}H_{21}O^+$ calcd.: 253.37 $[M+H]^+$, found: 253.16.; *m / z* for $C_{19}H_{26}ONa^+$ calcd.: 275.35 $[M+Na]^+$, found: 275.14.; HRMS (ESI): *m / z* for $C_{18}H_{20}ONa^+$ calcd.: 275.1406 $[M+Na]^+$, found: 275.1408.; FT-IR (ATR): $\tilde{\nu}$ = 3059 (w), 2917 (m), 2881 (m), 2821 (w), 1657 (s), 1596 (s), 1583 (vs), 1466 (), 1446 (m), 1422 (m), 1383 (m), 1366 (m), 1326 (s), 1316 (s), 1284 (vs), 1266 (m), 1209 (vs), 1175 (s), 1131 (m), 1098 (m), 1074 (m), 1056 (m), 1033 (m), 1016 (vs), 1000 (m), 981 (s), 915 (m), 886 (m), 857 (m), 824 (m), 801 (m), 768 (vs), 703 (vs), 692 (vs), 650 (s), 624 (s), 586 (m), 518 (m), 465 (w), 445 (w) cm^{-1}.

2,2,6-Trimethylcyclohexan-1-one (34)

Under an inert atmosphere (N_2), *n*-butyllithium (3.1 mL, 491 mg, 7.66 mmol, 2.5 M in hexane) was added dropwise to a solution of diisopropylamine (1.26 mL, 907 g, 8.96 mmol) in dry THF (6 mL) at −78 °C. After 1 h at 0 °C, 2,6-dimethylcyclohexanone (**32**, 1.00 mL, 912 mg, 7.23 mmol) was added at −78 °C. Methyl iodide (0.68 mL, 1.54 g, 10.8 mmol) was added at the same temperature. The reaction mixture was stirred for 1 h at −78 °C, warmed to room temperature and further stirred for 17 h, before quenching in a mixture of sat. NH_4Cl (200 mL), water (50 mL) and diethyl ether (150 mL). The layers were separated and the aqueous phase was extracted with diethyl ether (3 × 50 mL). The combined organic phases were washed with sat. NaCl (50 mL), dried ($MgSO_4$), and concentrated to yield **34** (1.00 g, 7.13 mmol, 99%) as colorless oil.

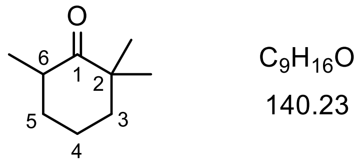

34

^{1}H NMR (500 MHz, $CDCl_3$) δ = 0.96 - 1.02 (m, 3H, 6-Me), 1.04 (s, 3H, 2-Me^1), 1.17 (s, 3H, 2-Me^2), 1.31 (qd, *J* = 13.1 Hz, 4.0 Hz, 1H, 5-H_A), 1.54 (td, *J* = 13.4 Hz, 4.2 Hz, 1H, 4-H_A), 1.60 - 1.68 (m, 1H, 3- H_A), 1.76 (dq, *J* = 13.4 Hz, 3.1 Hz, 1H, 4-H_B), 1.81 - 1.94 (m, 1H, 3-H_B), 2.04 (ddq, *J* = 12.5 Hz, 6.0 Hz, 3.1 Hz, 1H, 5-H_B), 2.65 (dp, *J* = 12.6 Hz, 6.3 Hz, 1H, 6-H) ppm; ^{13}C NMR (126 MHz, $CDCl_3$) δ = 15.0 (6-Me), 21.6 (C-3), 25.3 (2-Me^1), 25.7 (2-Me^2), 36.8 (C-5), 40.8 (C-6), 41.9 (C-4), 45.3 (C-2), 217.4 (C-1). Spectroscopic data are in agreement with literature [23].

1-Ethynyl-2,2,6-trimethylcyclohexan-1-ol (35)

Under an inert atmosphere (N_2), 2,2,6-trimethylcyclohexan-1-one (**34**, 1.00 g, 7.13 mmol) was dissolved in dry THF (20 mL) and ethynylmagnesium bromide (21.6 mL, 1.40 g, 10.8 mmol, 0.5 M in THF) was added dropwise at 0 °C. After stirring for 4 h at room temperature, the reaction mixture was quenched with 1 M HCl (25 mL). The layers were separated and the aqueous phase was extracted with EtOAc (3 × 50 mL), washed with sat. NaCl (50 mL), and dried ($MgSO_4$). Purification by column chromatography on silica gel (petroleum ether/EtOAc 40:1) afforded a diastereomeric mixture of **35** (847 mg, 5.07 mmol, 71%, diastereomeric ratio dr (diastereomer 1:diastereomer 2) 0.45:0.55, isolated yield) as colorless oil.

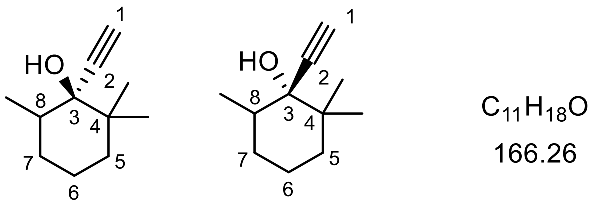

35

For the characterization, both diastereomers were isolated. The stereochemical elucidation was not performed. Therefore, a specific assignment to one diastereomer was not possible.

Diastereomer 1 of **35**

R_f 0.43 (petroleum ether/EtOAc 20:1); ^{1}H NMR (500 MHz, $CDCl_3$): δ = 1.07 (d, J = 6.7 Hz, 3H, 8-Me), 1.09 (d, J = 5.0 Hz, 6H, 4-Me), 1.16 - 1.34 (m, 2H, 5-H_A, 7-H_A), 1.40 - 1.52 (m, 3H., 6-H, 7-H_B), 1.59 (td, J = 12.5 Hz, 5.7 Hz, 1H, 5-H_B), 1.70 (s, 1H, OH), 1.90 (ddd, J = 12.5 Hz, 6.6 Hz, 3.9 Hz, 1H, 8-H), 2.41 (s, 1H, 1-H) ppm.; ^{13}C NMR (126 MHz, $CDCl_3$): δ = 17.0 (8-Me), 21.1 (6-H), 23.6 (4-Me^1), 26.1 (4-Me^2), 28.7 (C-7), 33.6 (C-5), 36.0 (C-8), 38.2 (C-4), 73.0 (C-1), 76.1 (C-2), 86.2 (C-3) ppm.; GC-MS (EI): m/z for $C_{11}H_{17}O^+$ calcd.: 165.3 $[M]^+$, found: 165.1.; HRMS (ESI): m/z for $C_{11}H_{18}O$ calcd.: 166.1352 $[M]^+$, found: 166.1351.; FT-IR (ATR): $\tilde{\nu}$ = 3487 (b), 3307 (m), 2967 (s), 2933 (vs), 2866 (m), 1704 (w), 1460 (m), 1376 (m), 1366 (m), 1298 (m), 1218 (m), 1181 (w), 1158 (m), 1132 (m), 1104 (w), 1075 (m), 1061 (m), 1028 (m), 1017 (m), 965 (vs), 954 (vs), 924 (w), 884 (m), 857 (w), 840 (w), 735 (m), 651 (s), 626 (s), 565 (m), 550 (w), 489 (w), 469 (w), 421 (w) cm^{-1}.

Diastereomer 2 of **35** (indicated with *)

$^*R_f$ 0.47 (petroleum ether/EtOAc 20:1); ^{*1}H NMR (500 MHz, $CDCl_3$): δ = 1.00 (s, 3H, 4-Me^1), 1.04 (d, J = 6.5, 3H, 6-Me), 1.12 (s, 3H, 4-Me^2), 1.28 - 1.39 (m, 2H, 5-H_A, 7-H_A), 1.39 - 1.51 (m, 2H, 6-H).1.54 - 1.67 (m, 2H, 5-H_B, 7-H_B), 1.81 - 1.93 (m, 2H, 8-H, OH), 2.48 (s, 1H, 1-H) ppm. ^{*13}C NMR (126 MHz, $CDCl_3$) δ = 16.3 (8-Me), 19.7 (4-Me^1), 21.2 (6-H), 26.7 (4-Me^2), 32.6 (C-5), 36.7 (C-8), 37.9 (C-7), 38.7 (C-4), 75.2 (C-1), 78.6 (C-2), 84.1 (C-3) ppm.; *GC-MS (EI): m/z for $C_{11}H_{17}O^+$ calcd.: 165.3 $[M]^+$, found: 165.1.; *HRMS (ESI): m/z for $C_{11}H_{18}O$ calcd.: 166.1352 [M], found: 166.1352. *FT-IR (ATR): $\tilde{\nu}$ = 3615 (w), 3477 (b), 3307 (s), 2962 (s), 2930 (vs), 2867 (s), 1460 (s), 1385 (m), 1367 (m), 1332 (m), 1301 (m), 1275 (m), 1220 (m), 1183 (w), 1136 (w), 1073 (s), 1059 (s), 1032 (m), 991 (s), 973 (s), 961 (w), 922 (w), 885 (w), 855 (w), 787 (w), 651 (s), 626 (s), 552 (w), 476 (w), 434 (w), 416 (w) cm^{-1}.

2-Ethynyl-1,1,3-trimethylcyclohexane (25q)

Under an inert atmosphere (N_2), 1-ethynyl-2,2,6-trimethylcyclohexan-1-ol (**35**, 686 mg, 4.13 mmol) in dichloromethane (10 mL) was treated with NEt_3 (2.90 mL, 2.09 g, 20.6 mmol) and MsCl (0.80 mL 1.18 g, 10.3 mmol) at 0 °C. After stirring for 1 h, additional NEt_3 (0.50 mL, 0.36 g, 3.59 mmol) and MsCl (0.50 mL 0.74 g, 6.4 mmol) were added. Upon completion the reaction was quenched with sat. NH_4Cl (25 mL) and the phases were separated. The aqueous phase was extracted with dichloromethane (3 × 20 mL), washed with sat. NaCl (20 mL), and dried ($MgSO_4$). Purification by column chromatography on silica gel (petroleum ether) afforded **25q** (300 mg, 2.02 mmol, 49%) as colorless oil.

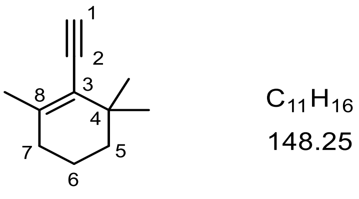

25q

R_f 0.69 (petroleum ether); ^{1}H NMR (400 MHz, $CDCl_3$) δ = 1.08 - 1.26 (m, 7H, 4-Me; 6-H_A), 1.42 - 1.65 (m, 3H, 5-H, 6-H_B), 1.89 (s, 3H, 8-Me), 1.98 - 2.05 (m, 2H, 7-H), 3.07 (s, 1H, 1-H) ppm.; ^{13}C NMR (126 MHz, $CDCl_3$): δ = 18.9 (C-6), 22.6 (8-Me), 28.8 (4-Me), 31.9 (C-7), 33.6, 37.6 (C-5), 80.5 (C-1), 82.5 (C-2), 123.1 (C-3), 142.8 (C-8) ppm.; GC-MS (EI): *m / z* for $C_{11}H_{16}$: calcd.: 148.3 [M], found: 148.1.; HRMS (ESI): *m / z* for $C_{11}H_{16}^+$ calcd.: 148.1246 [M], found: 148.1247.; FT-IR (ATR): $\tilde{\nu}$ = 3307 (m), 2932 (vs), 2864 (s), 2222 (m), 2141 (w), 2087 (m), 2072 (s), 2049 (w), 2028 (m), 1964 (w), 1933 (w), 1899 (w), 161 (m), 1592 (w), 1507 (m), 1459 (s), 1377 (m), 1360 (m), 1298 (m), 1260 (m), 1228 (s), 1196 (m), 1173 (w), 1131 (w), 1078 (w), 1027 (m), 925 (w), 895 (w), 849 (m), 827 (m), 746 (w), 706 (w), 632 (m), 594 (w), 576 (w), 534 (w), 482 (w), 455 (w), 424 (w) cm^{-1}.

(*E*)-4-(2,6,6-Trimethylcyclohex-1-en-1-yl)but-3-en-2-one (3)

According to GP3, 2-ethynyl-1,1,3-trimethylcyclohexane (**25o**, 80 mg, 0.54 mmol) was treated with Schwartz's reagent (141 mg, 0.55 mmol), acetyl chloride (**22**, 30.3 µL, 38.5 mg, 0.49 mmol) and $Pd(PPh_3)_2Cl_2$ (17.2 mg, 24.5 µmol). Purification by column chromatography on silica gel (petroleum ether/EtOAc 30:1) afforded **3** (17 mg, 0.88 mmol, 18%) as colorless oil.

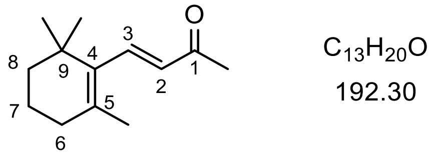

3

R_f 0.3 (petroleum ether/EtOAc 20:1); ^{1}H NMR (500 MHz, $CDCl_3$): δ = 1.06 (s, 6H, 9-Me), 1.44 - 1.50 (m, 2H, 8-H), 1.57 - 1.66 (m, 2H, 7-H), 1.75 (d, *J* = 1.0 Hz, 3H, 5-Me), 2.06 (t, *J* = 6.4, 2H, 6-H), 2.29 (s, 3H, 1-Me), 6.11 (d, *J* = 16.4 Hz, 1H, 2-H), 7.23 - 7.30 (m, 1H. 3-H) ppm.; ^{13}C NMR (126 MHz, $CDCl_3$): δ = 18.9 (C-7), 21.8 (5-Me), 27.2 (1-Me), 28.8 (9-Me), 33.6 (C-6), 34.1 (C-9), 39.7 (C-8), 131.6 (C-2), 135.9 (C-5), 136.1 (C-4), 143.2 (C-3), 198.8 (C-1) ppm. Spectroscopic data are in agreement with literature [24].

(*E*)-1-Phenyl-3-(2,6,6-trimethylcyclohex-1-en-1-yl)prop-2-en-1-one (27qa)

According to GP3, 2-ethynyl-6,6-dimethylbicyclo[3.1.1]hept-2-ene **(25q**, 100 mg, 0.68 mmol) was treated with Schwartz's reagent (177 mg, 0.69 mmol), benzoyl chloride (**26a**, 67.9 µL, 86.2 mg, 0.61 mmol) and $Pd(PPh_3)_2Cl_2$ (21.5 mg, 30.7 µmol). Purification by column chromatography on silica gel (petroleum ether/EtOAc 40:1) afforded **27qa** (39 mg, 0.15 mmol, 25%) as yellow oil.

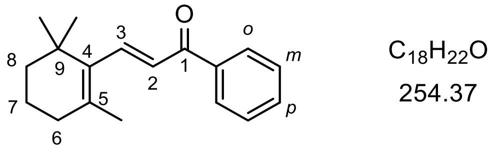

27qa

R_f 0.47 (petroleum ether); ^{1}H NMR (500 MHz, $CDCl_3$) δ = 1.13 (s, 6H, 9-Me), 1.48 - 1.55 (m, 2H, 8-H), 1.61 - 1.69 (m, 2H, 7-H), 1.85 (d, J = 1.1 Hz, 3H, 5-Me), 2.03 - 2.16 (m, 2H, 6-H), 6.93 (d, J = 15.8 Hz, 1H, 2-H), 7.44 - 7.50 (m, 2H, *m*-H), 7.52 - 7.62 (m, 2H, 3-H, *p*-H), 7.91 - 7.97 (m, 2H, *o*-H) ppm.; ^{13}C NMR (126 MHz, $CDCl_3$) δ = 18.9 (C-7), 21.9 (5-Me), 28.9 (9-Me), 33.8 (C-6), 34.2 (C-9), 39.9 (C-8), 126.2 (C-2), 128.5 (C-*m*), 128.5 (C-*o*), 132.5 (C-*p*), 136.8 (C-5), 136.9 (C-4), 138.5 (C-*i*), 144.6 (C-*p*), 190.8 (C-1) ppm.; MS (EI): *m / z* for $C_{18}H_{23}O^+$: calcd.: 255.17 $[M+H]^+$, found: 255.17.; HRMS (ESI): *m / z* for $C_{18}H_{23}O^+$ calcd.: 255.1741 $[M+H]^+$, found: 255.1743.; FT-IR (ATR): $\tilde{\nu}$ =3060 (w), 2958 (s), 2930 (s), 2866 (m), 2825 (w), 2175 (vs), 1663 (vs), 1600 (vs), 1579 (s), 1447 (w), 1375 (m), 1361 (w), 1315 (s), 1278 (s), 1262 (s), 1216 (s), 1178 (m), 1141 (w), 1072 (w), 1036 (m), 1009 (s), 984 (m), 938 (w), 869 (w), 850 (w), 799 (w), 777 (w), 698 (s), 633 (w) cm^{-1}.

1.8. Synthesis of bromo diene S1

((1*E*,3*E*)-4-bromobuta-1,3-dien-1-yl)benzene (S1)

Under an inert atmosphere (N_2), enyne **25a** (100 mg, 0.78 mmol) was dissolved in anhydrous toluene under absence of light and Schwartz's reagent (205 mg, 0.79 mmol) was added. After stirring for 1 h at 50 °C, the formed dark red solution showed complete conversion. NBS (141 mg, 0.79 mmol) was added at room temperature and the reaction mixture was stirred for 18 h, filtered through a pad of SiO_2, and washed with EtOAc. Purification by column chromatography on silica gel (petroleum ether) afforded **S1** (131 mg, 0.63 mmol, 88%) as yellow solid.

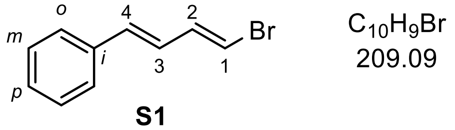

S1

R_f 0.62 (petroleum ether); ^{1}H NMR (700 MHz, $CDCl_3$): δ = 6.37 (d, J = 13.5 Hz, 1H, 4-H), 6.51 (d, J = 15.6, 1H, 1-H), 6.61 (dd, J = 15.6 Hz, 10.7 Hz, 1H, 2-H), 6.81 (dd, J = 13.5 Hz, 10.7 Hz, 1H, 3-H), 7.18 – 7.21 (m, 1H, *p*-H), 7.24 – 7.28 (m, 2H, *m*-H), 7.30 – 7.35 (m, 2H, *o*-H) ppm. ^{13}C NMR (176 MHz, $CDCl_3$): δ = 109.0 (C-4), 126.1 (C-2), 126.5 (C-*o*), 128.1 (C-*p*), 128.8 (C-*m*), 133.4 (C-3), 137.7 (C-1) ppm. Spectroscopic data are in agreement with literature [25]

2. Control experiments

2.1. Trapping experiments with NBS

In order to investigate the conversion of starting material **25** in the first step of the reaction sequence, the formed (*E*)-alkenylzirconium **24** was reacted with *N*-bromosuccinimide (NBS, Scheme S1).

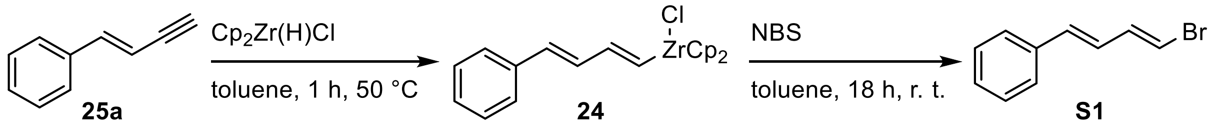

Scheme S1

Therefore, enyne **25a** was dissolved in toluene, shielded from light and treated with Schwartz's reagent for 1 h at 50 °C. After cooling to room temperature, NBS was added, and the reaction mixture was stirred overnight. The crude ^{1}H NMR spectrum of **S1** showed no proton signals of **25a**, thus starting material **25a** was fully converted to **24**. However, GC–MS analysis of the crude product showed, apart from major product **S1** only traces of three side products. One of these side products showed an *m*/*z* = 208 [M − H]$^-$ peak, which was presumably caused by the regioisomer of **S1**, but this side product could not be isolated. Column chromatography yielded **S1** in 88% yield, indicating a major conversion of **25a** to **24**.

2.2. Crude NMR spectra

Crude products were filtered through a pad of silica before NMR analysis. Figure S1 shows (**A**) the crude ^{1}H NMR spectrum of **27ap** and (**B**) an expansion of the area between 6.45–7.65 ppm. The double bond geometry was determined for example by 2-H signal and its coupling constant ^3J_H = 14.9 Hz (highlighted as 2-H in Figure S1) indicating an (*E*)-geometry. Furthermore, 2-H signals of the (*Z*)-**27ap** isomer could not be detected, revealing (*E*)- **27ap** as only isomer.

However, in case of products with low yields, such as **27ac** with 17%, crude product ^{1}H NMR and GC–MS analysis revealed the formation of side products and decomposition of the starting material in the reaction sequence. Nevertheless, no configurational isomers of **27ac** were detected by GC–MS, indicating a selective formation of the (*E*)-isomer. Figure S2 shows the crude ^{1}H NMR spectrum of **27ac** (**A**) and an expansion of the area between 6.40–7.6 ppm (**B**). The (*E*)-geometry of the double bond was determined by the 2-H signal and its coupling constant ^3J_H = 15.2 Hz (highlighted as 2-H in Figure S2).

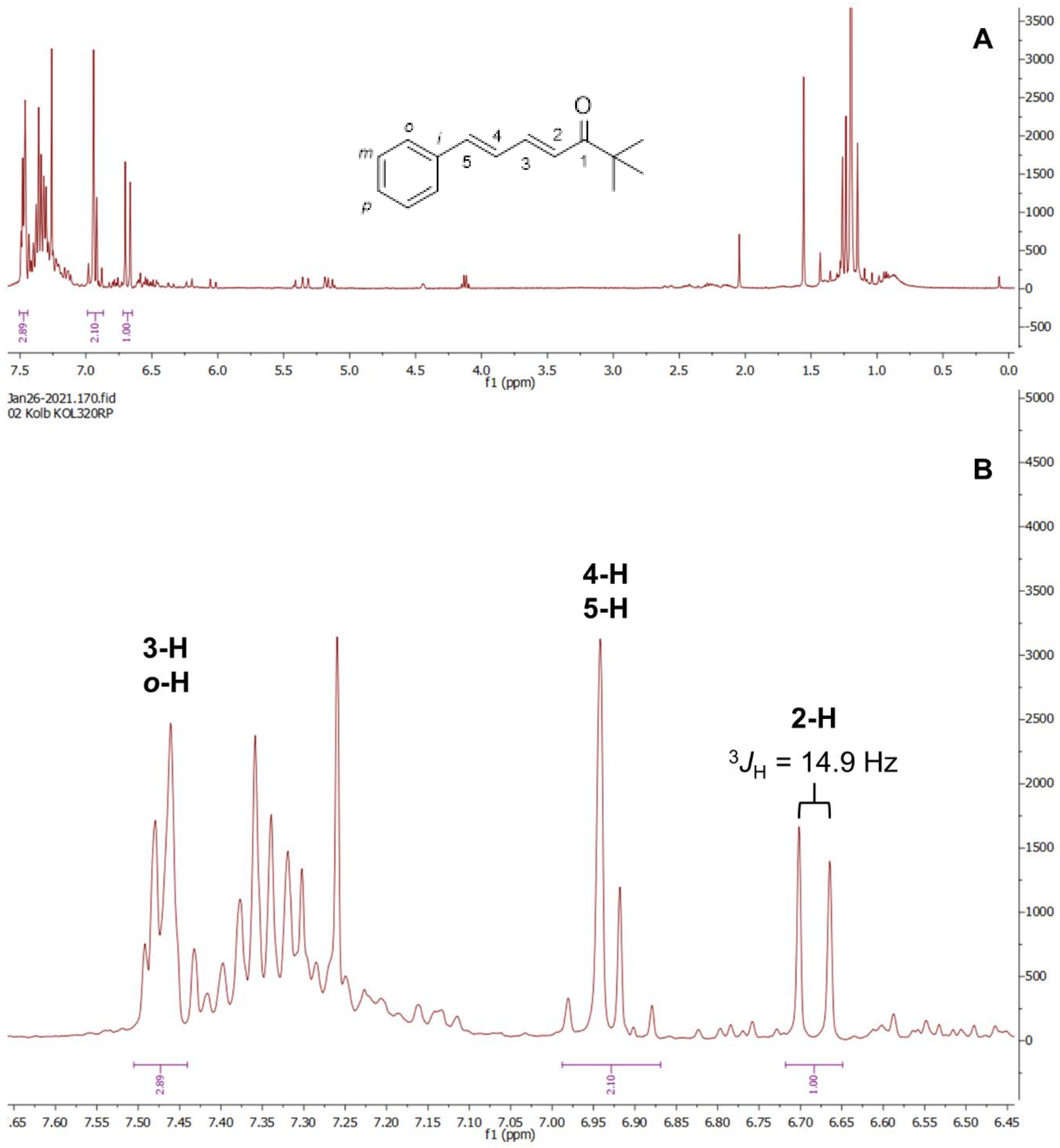

Figure S1: (**A**) Crude ^{1}H NMR spectrum (400 MHz, $CDCl_3$) of **27ap**. (B) Section of the crude ^{1}H NMR spectrum of **27ap** between 6.45–7.65 ppm, indicating the (*E*)-configuration of the C2–C3 double bond with a coupling constant of $^{3}J_H$ = 14.9 Hz.

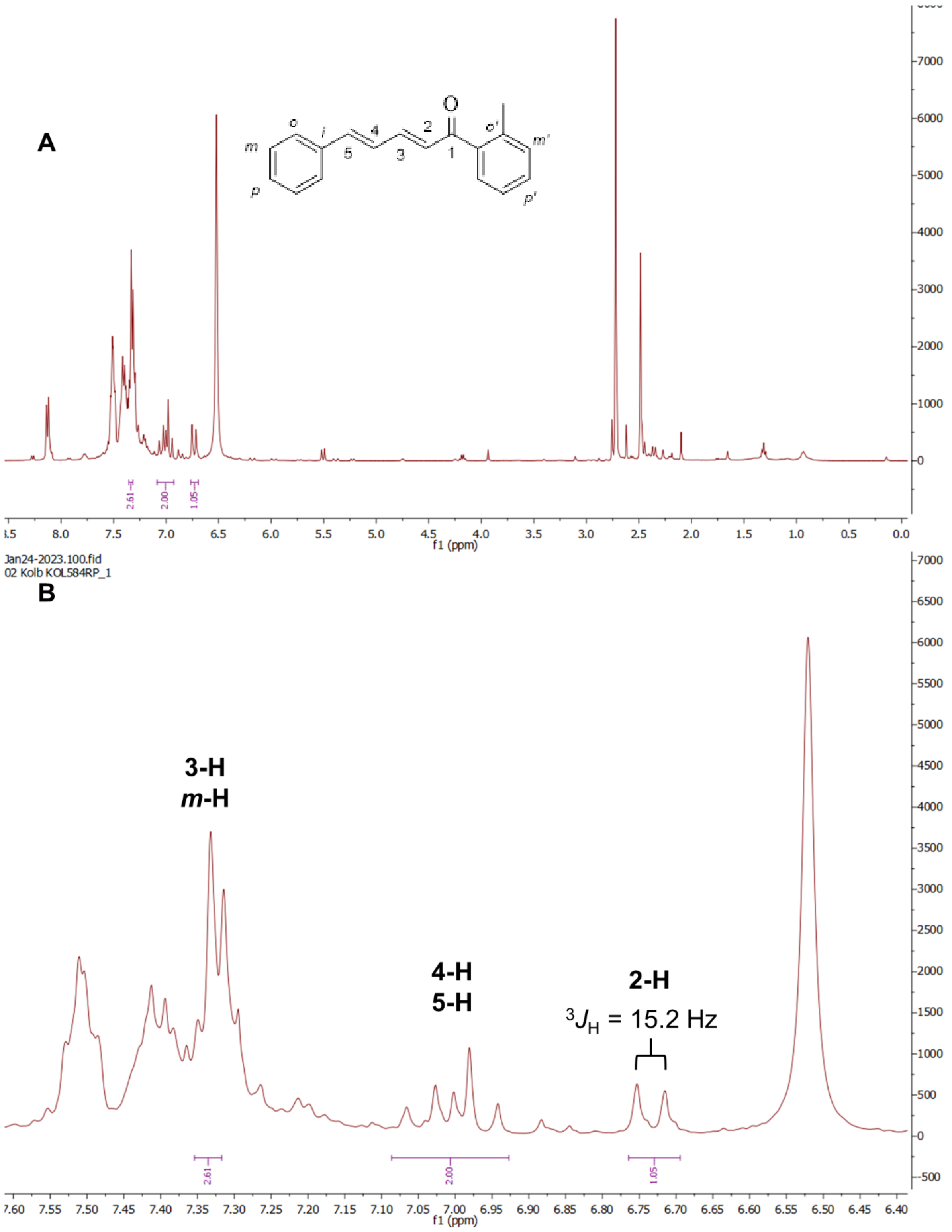

Figure S2: (**A**) Crude ^{1}H NMR spectrum (400 MHz, $CDCl_3$) of **27ac**. (B) Section of the crude ^{1}H NMR spectrum of **27ac** between 6.40–7.60 ppm, indicating the (*E*)-configuration of the C2–C3 double bond with a coupling constant of $^{3}J_{H}$ = 15.2 Hz.

3. Analytical data

(*E*)-Undec-3-en-1-yne (25e)

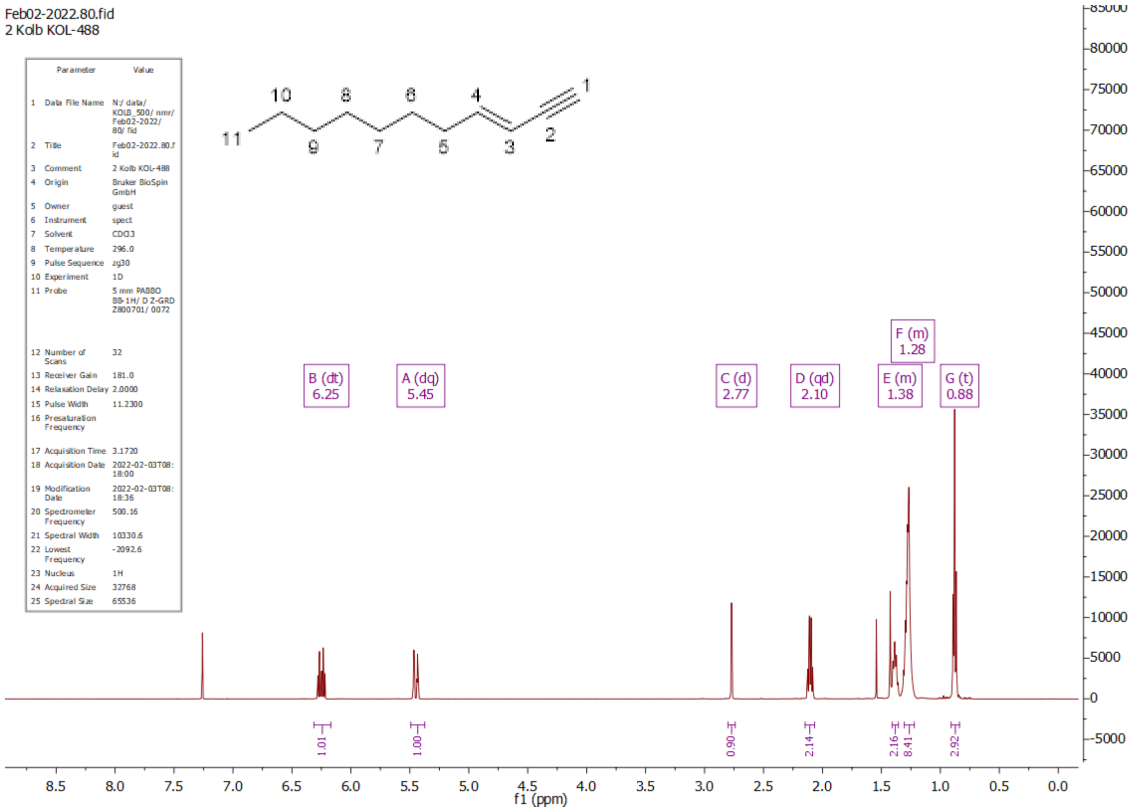

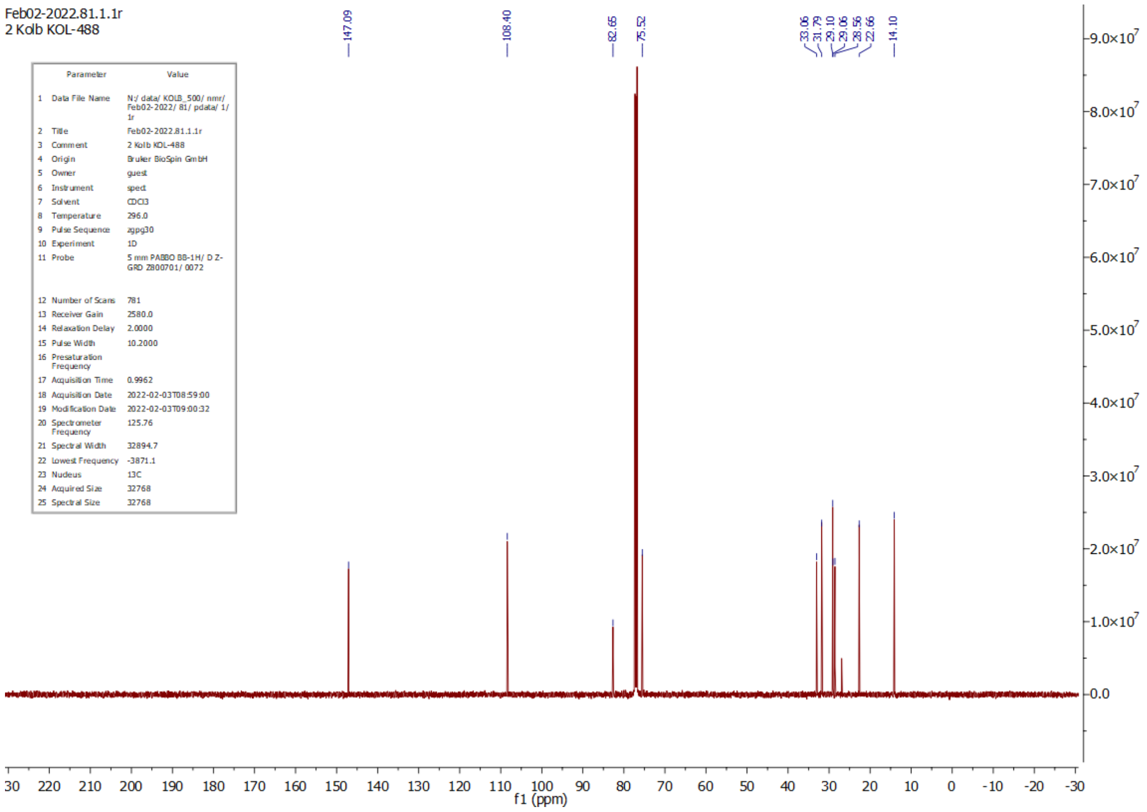

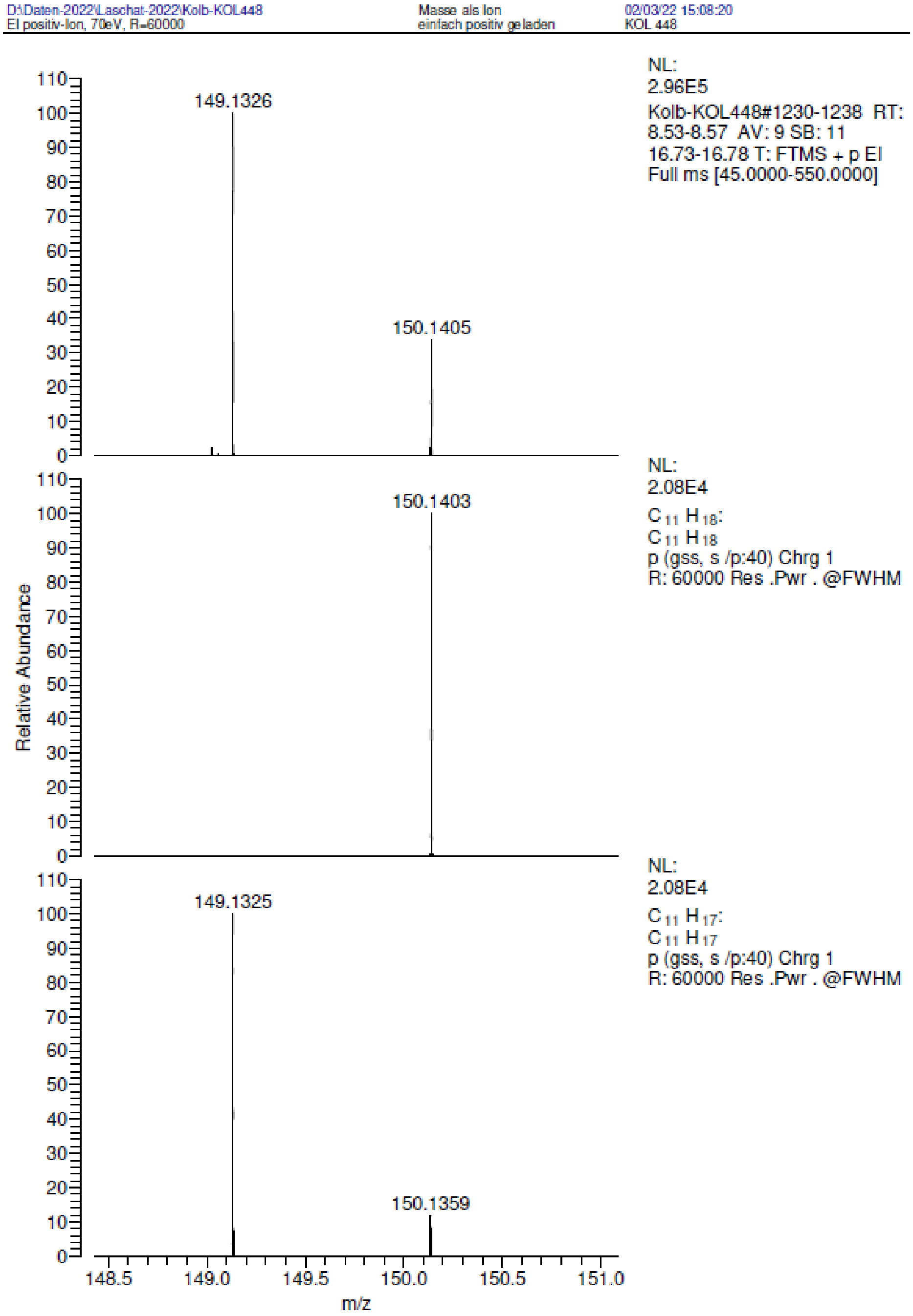

D:\Daten-2022\Laschat-2022\Kolb-KOL448
EI positiv-Ion, 70eV, R=60000
Masse als Ion
einfach positiv geladen
02/03/22 15:08:20
KOL 448
NL:
2.96E5
Kolb-KOL448#1230-1238 RT: 8.53-8.57 AV: 9 SB: 11 16.73-16.78 T: FTMS + p EI Full ms [45.0000-550.0000]
149.1326
150.1405
NL:
2.08E4
C 11 H 18:
C 11 H 18
p (gss, s /p:40) Chrg 1
R: 60000 Res .Pwr . @FWHM
150.1403
NL:
2.08E4
C 11 H 17:
C 11 H 17
p (gss, s /p:40) Chrg 1
R: 60000 Res .Pwr . @FWHM
149.1325
150.1359
Relative Abundance
m/z

(1*E*,3*E*)-1-Phenyltrideca-1,3-dien-5-one (27an)

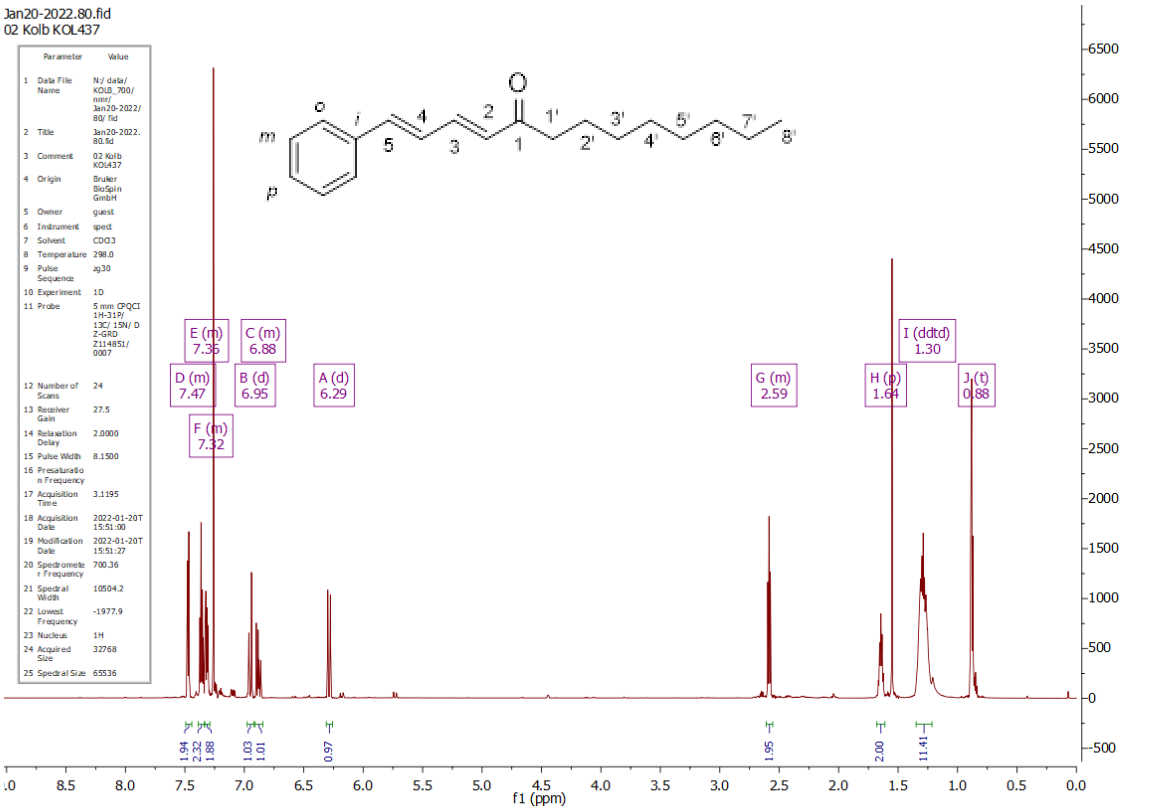

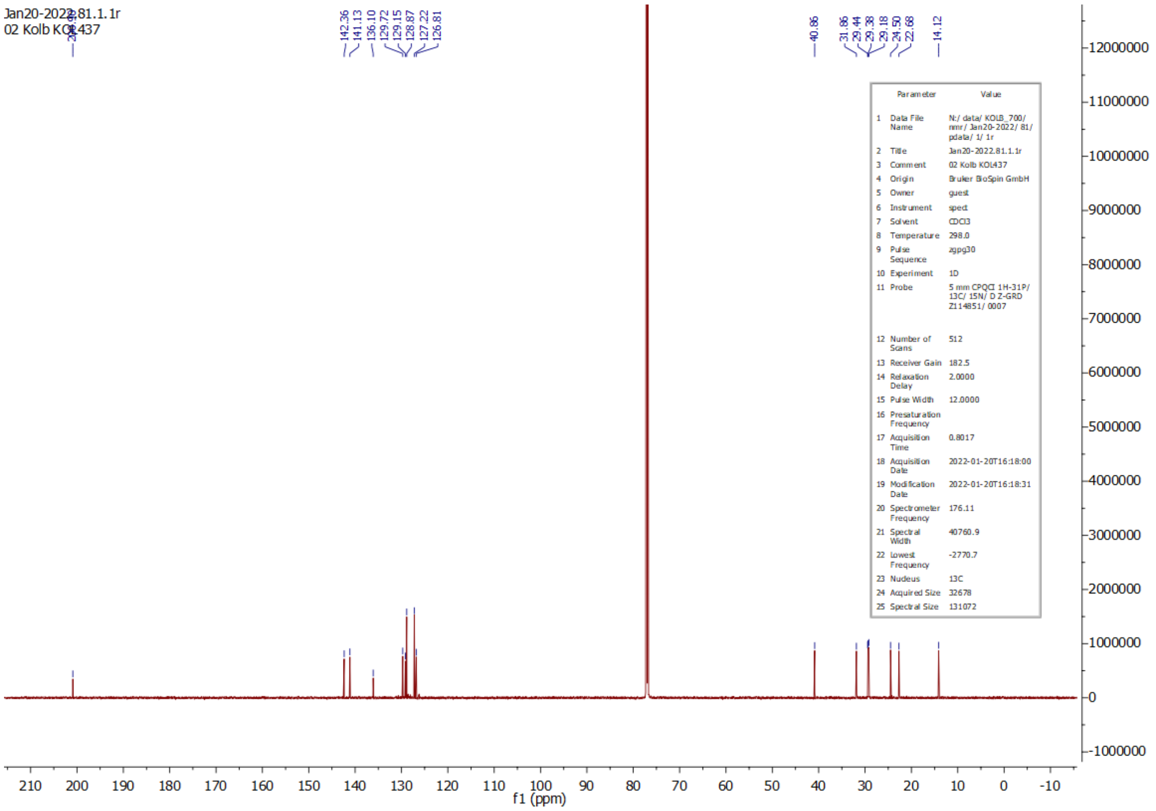

Massenspektrometrie - Universität Stuttgart

Analysis Info

Analysis Name	Kolb-KOL437_1_01_36765.d	Acquisition Date	1/27/2022 3:10:41 PM
Method	tune-low-oktober-2019.m	Operator	BDAL@DE
Sample Name	Kolb-KOL437	Instrument	micrOTOF-Q 228888.00043
Comment			

Acquisition Parameter

Source Type	ESI	Ion Polarity	Positive	Set Nebulizer	0.4 Bar
Focus	Not active	Set Capillary	4500 V	Set Dry Heater	200 °C
Scan Begin	50 m/z	Set End Plate Offset	-500 V	Set Dry Gas	4.0 l/min
Scan End	1500 m/z	Set Collision Cell RF	180.0 Vpp	Set Divert Valve	Waste

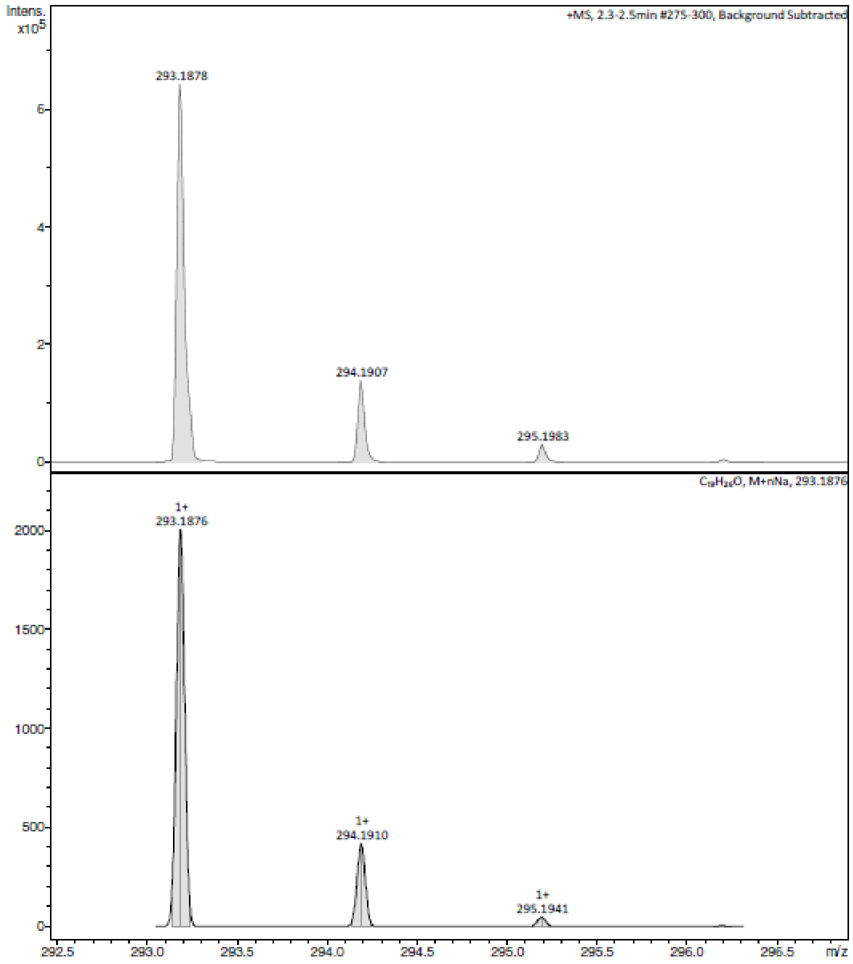

Bruker Compass DataAnalysis 4.2 printed: 1/27/2022 3:38:05 PM Page 1 of 1

(*E*)-3-(6,6-Dimethylbicyclo[3.1.1]hept-2-en-2-yl)-1-phenylprop-2-en-1-one (27pa)

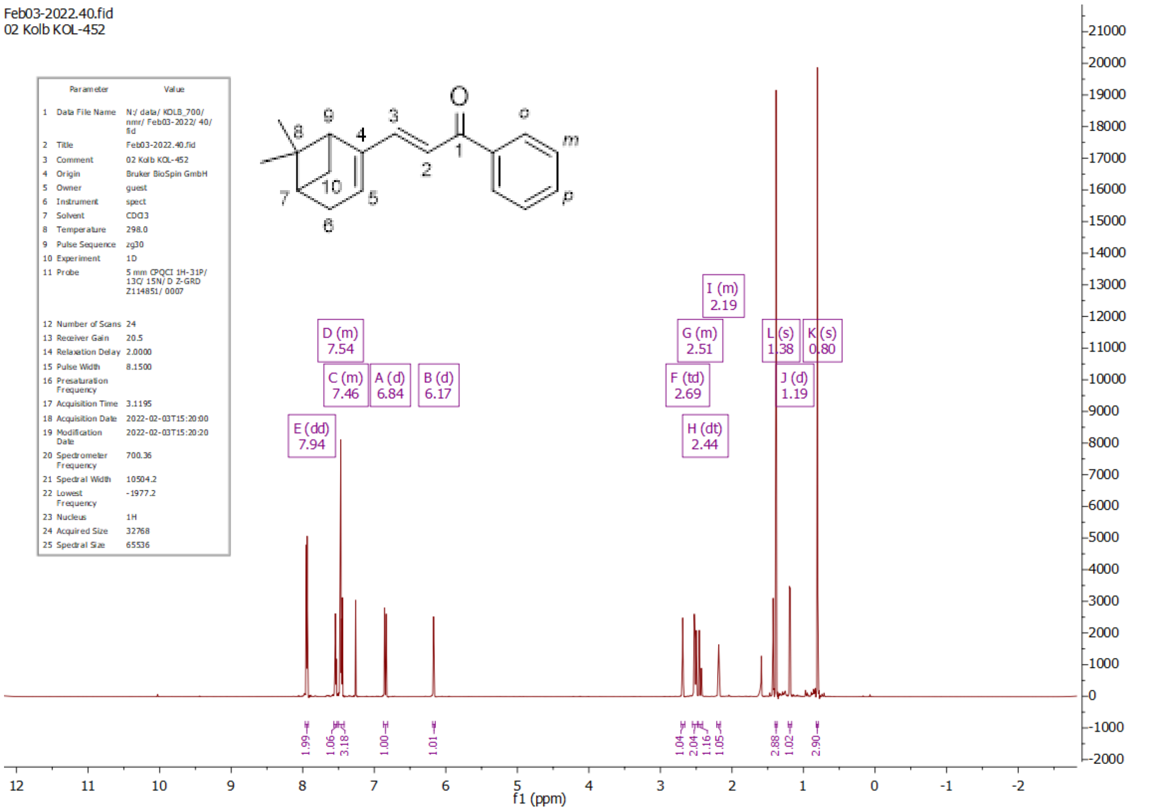

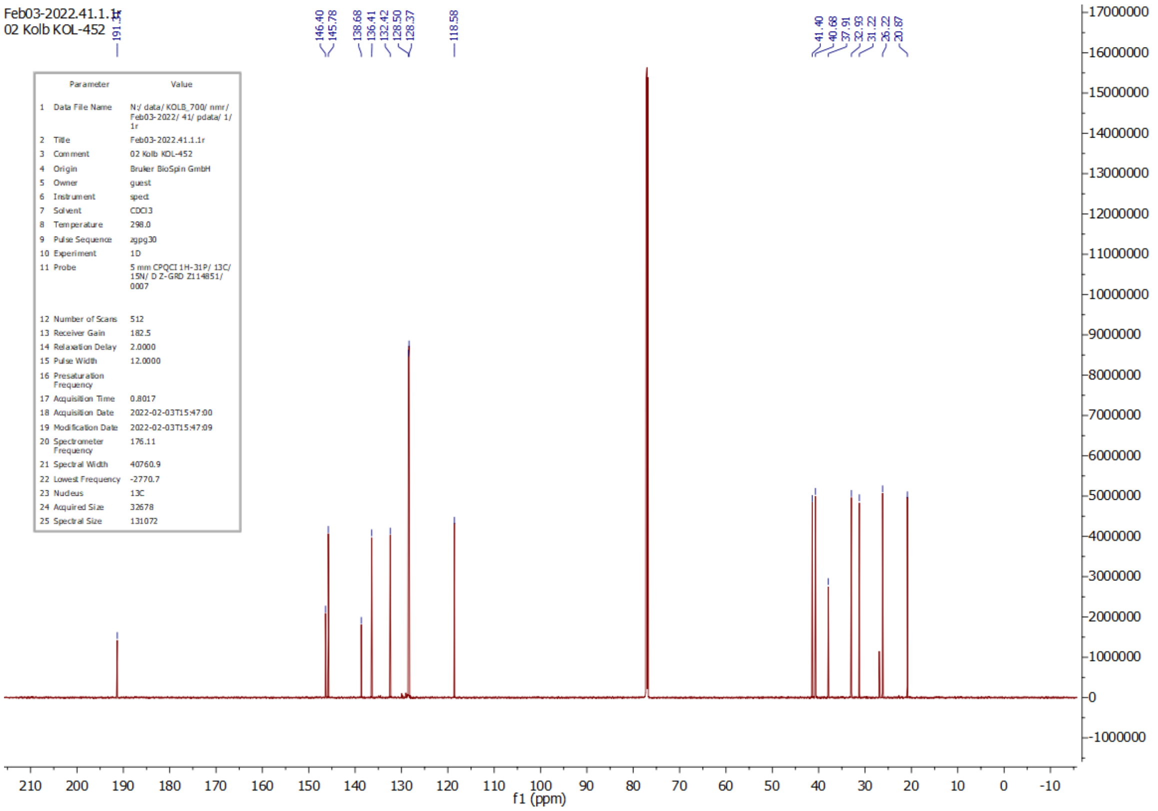

Massenspektrometrie - Universität Stuttgart

Analysis Info

Analysis Name	Kolb-KOL452_4_01_36885.d	Acquisition Date	2/4/2022 12:42:34 PM
Method	tune-low-oktober-2019.m	Operator	BDAL@DE
Sample Name	Kolb-KOL452	Instrument	micrOTOF-Q 228888.00043
Comment			

Acquisition Parameter

Source Type	ESI	Ion Polarity	Positive	Set Nebulizer	0.4 Bar
Focus	Not active	Set Capillary	4500 V	Set Dry Heater	200 °C
Scan Begin	50 m/z	Set End Plate Offset	-500 V	Set Dry Gas	4.0 l/min
Scan End	1500 m/z	Set Collision Cell RF	180.0 Vpp	Set Divert Valve	Waste

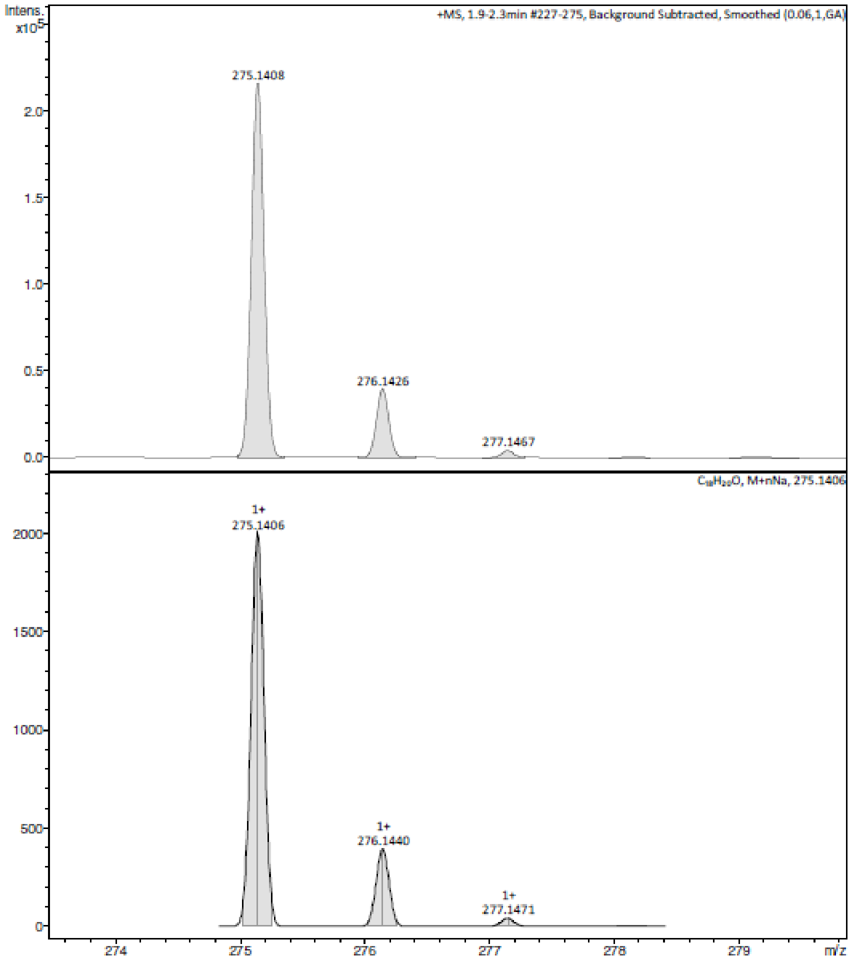

Bruker Compass DataAnalysis 4.2 printed: 2/4/2022 1:54:47 PM Page 1 of 1

1-Ethynyl-2,2,6-trimethylcyclohexan-1-ol (35) (diastereomer 1)

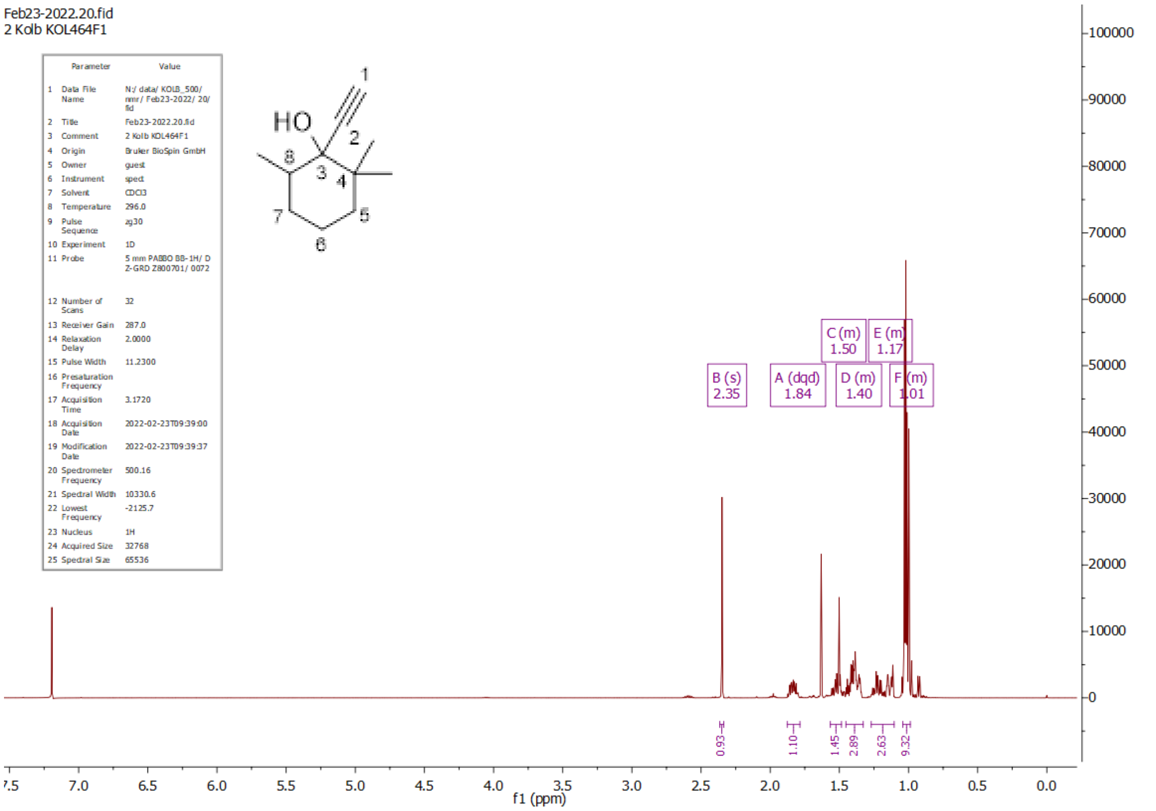

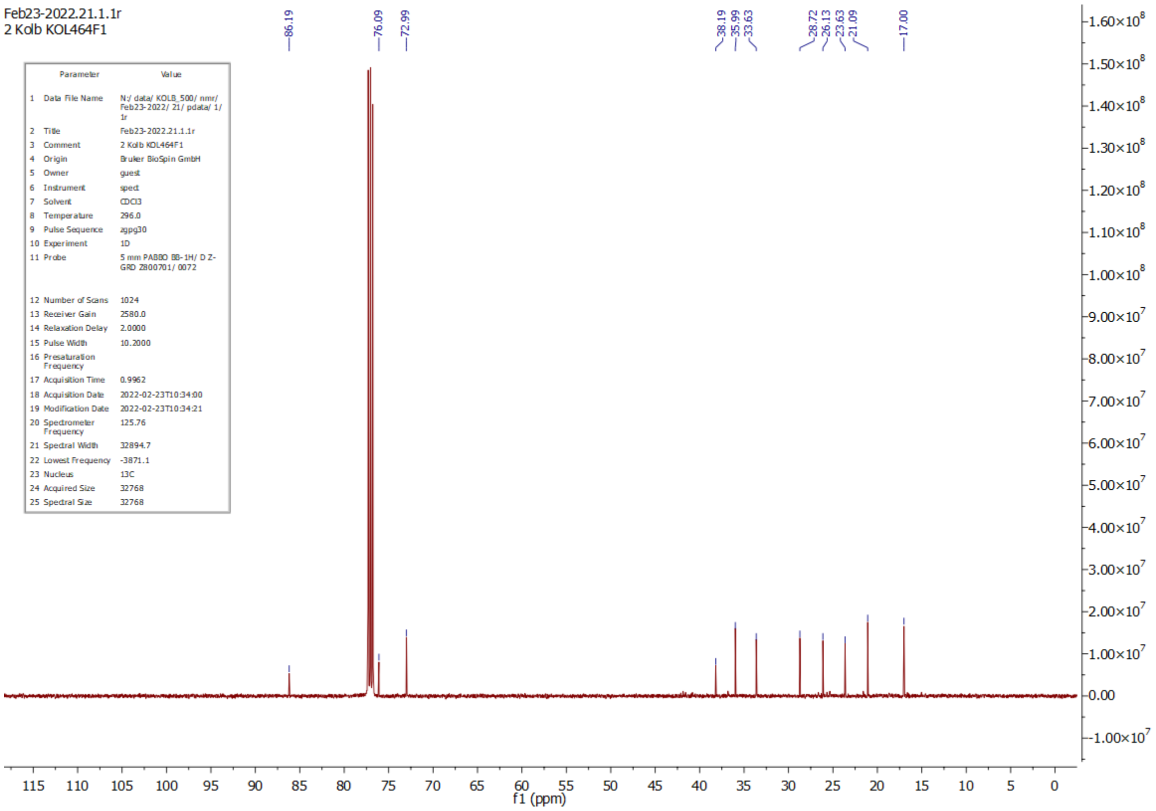

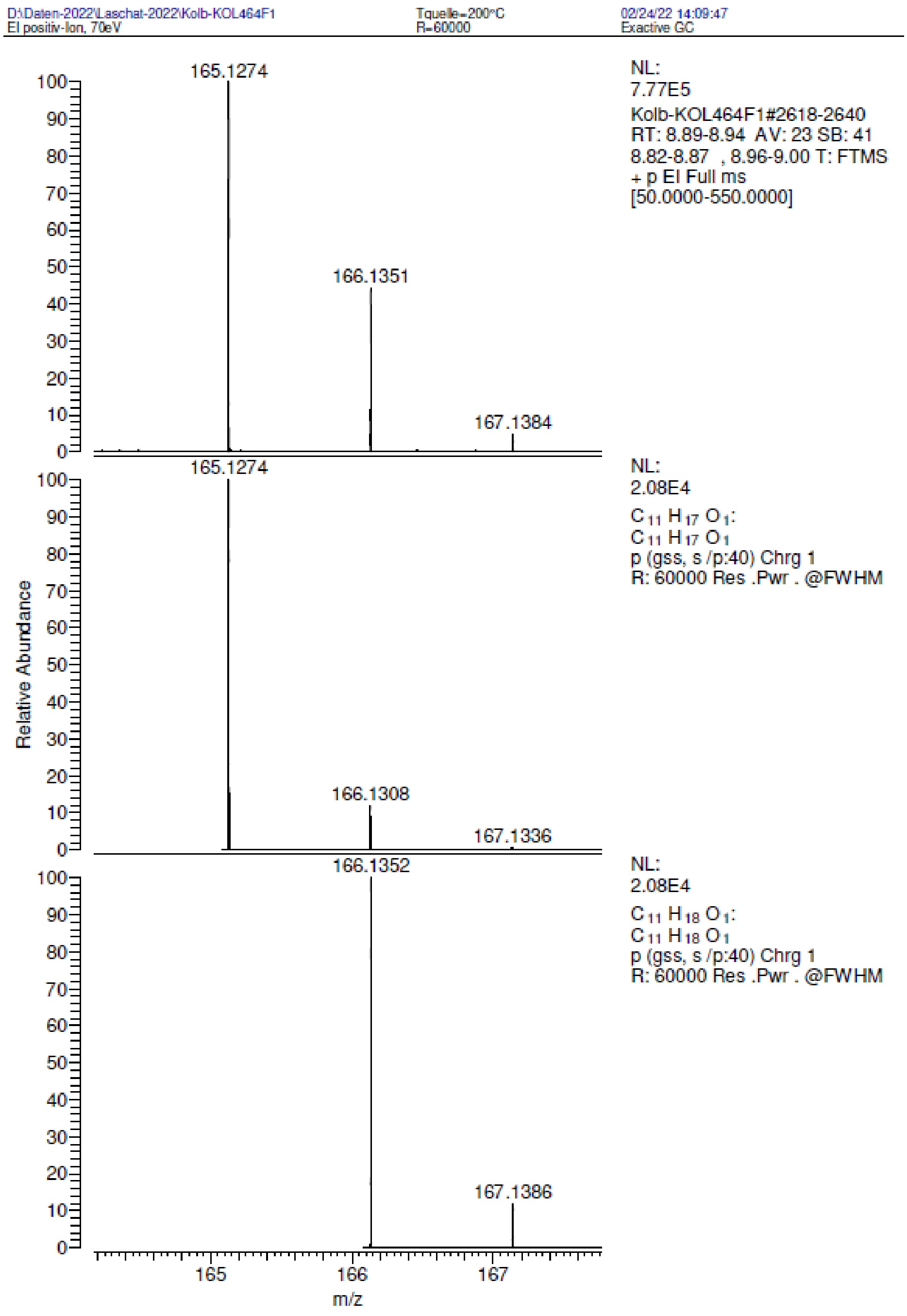

D:\Daten-2022\Laschat-2022\Kolb-KOL464F1
EI positiv-Ion, 70eV
Tquelle=200°C
R=60000
02/24/22 14:09:47
Exactive GC
165.1274
166.1351
167.1384
NL:
7.77E5
Kolb-KOL464F1#2618-2640
RT: 8.89-8.94 AV: 23 SB: 41
8.82-8.87 , 8.96-9.00 T: FTMS
+ p EI Full ms
[50.0000-550.0000]
165.1274
166.1308
167.1336
NL:
2.08E4
C 11 H 17 O 1:
C 11 H 17 O 1
p (gss, s /p:40) Chrg 1
R: 60000 Res .Pwr . @FWHM
166.1352
167.1386
NL:
2.08E4
C 11 H 18 O 1:
C 11 H 18 O 1
p (gss, s /p:40) Chrg 1
R: 60000 Res .Pwr . @FWHM
Relative Abundance
m/z

1-Ethynyl-2,2,6-trimethylcyclohexan-1-ol (35) (diastereomer 2)

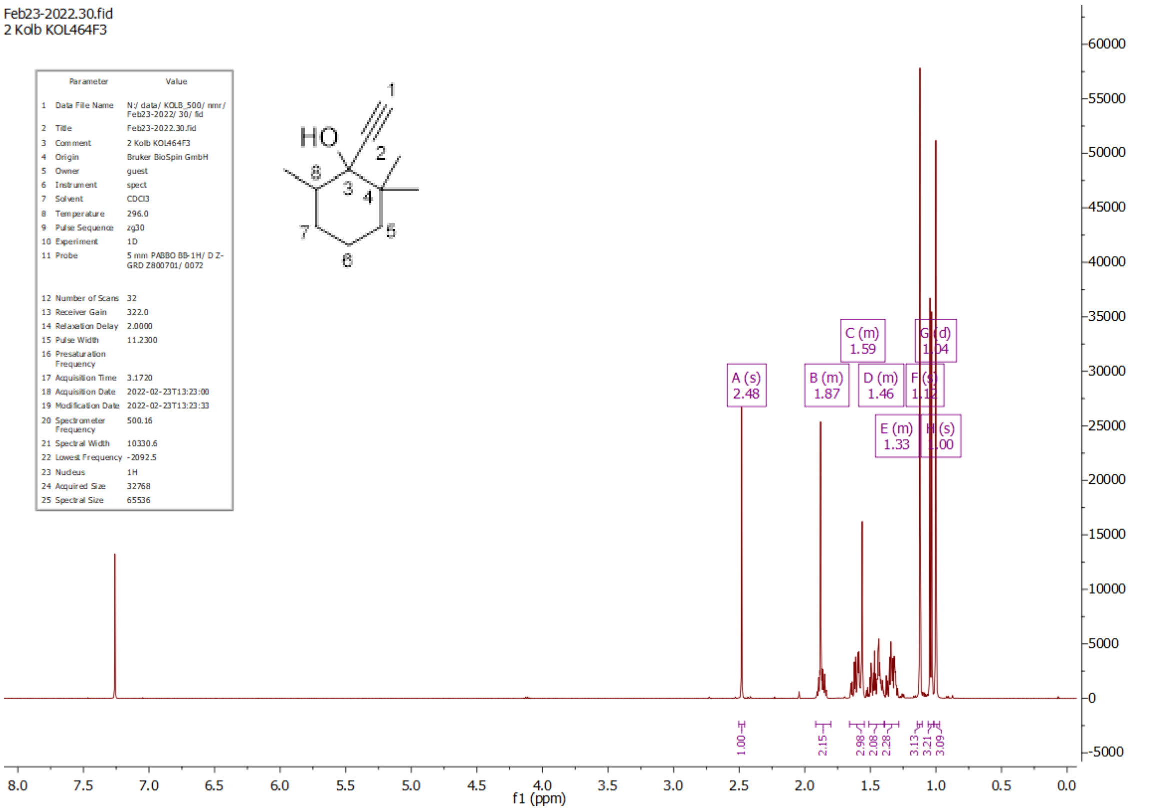

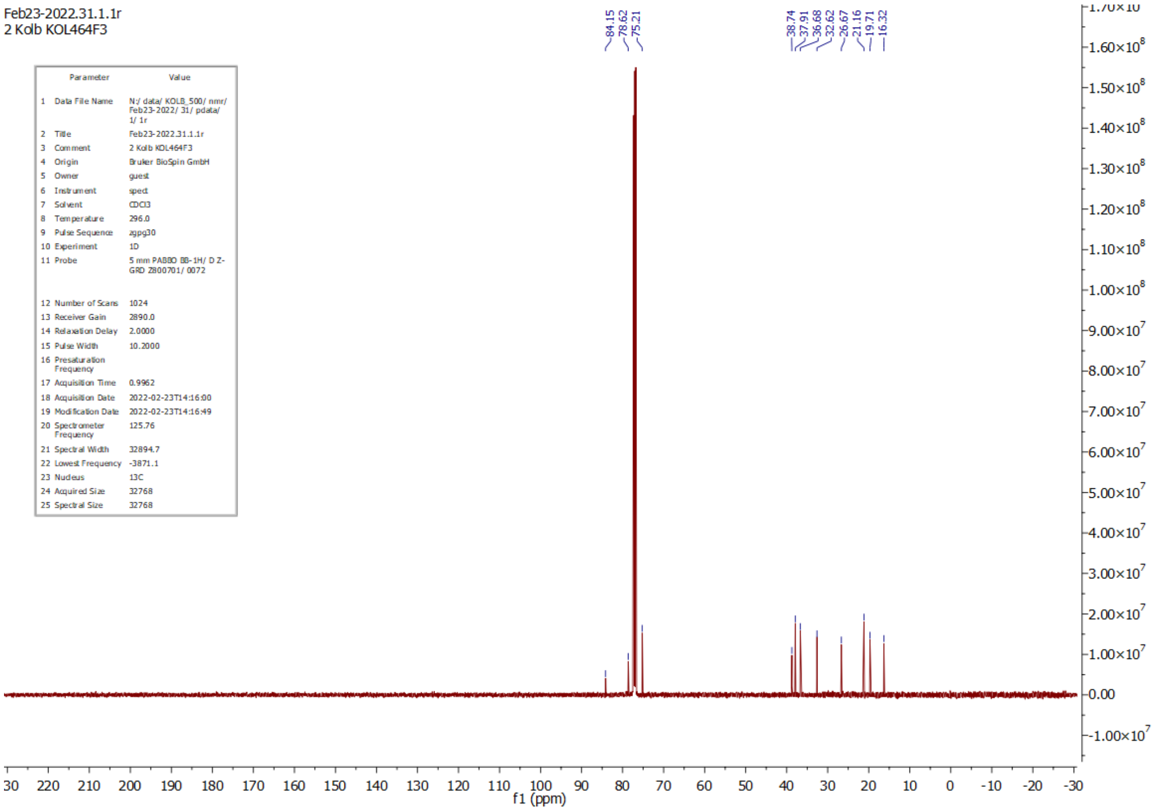

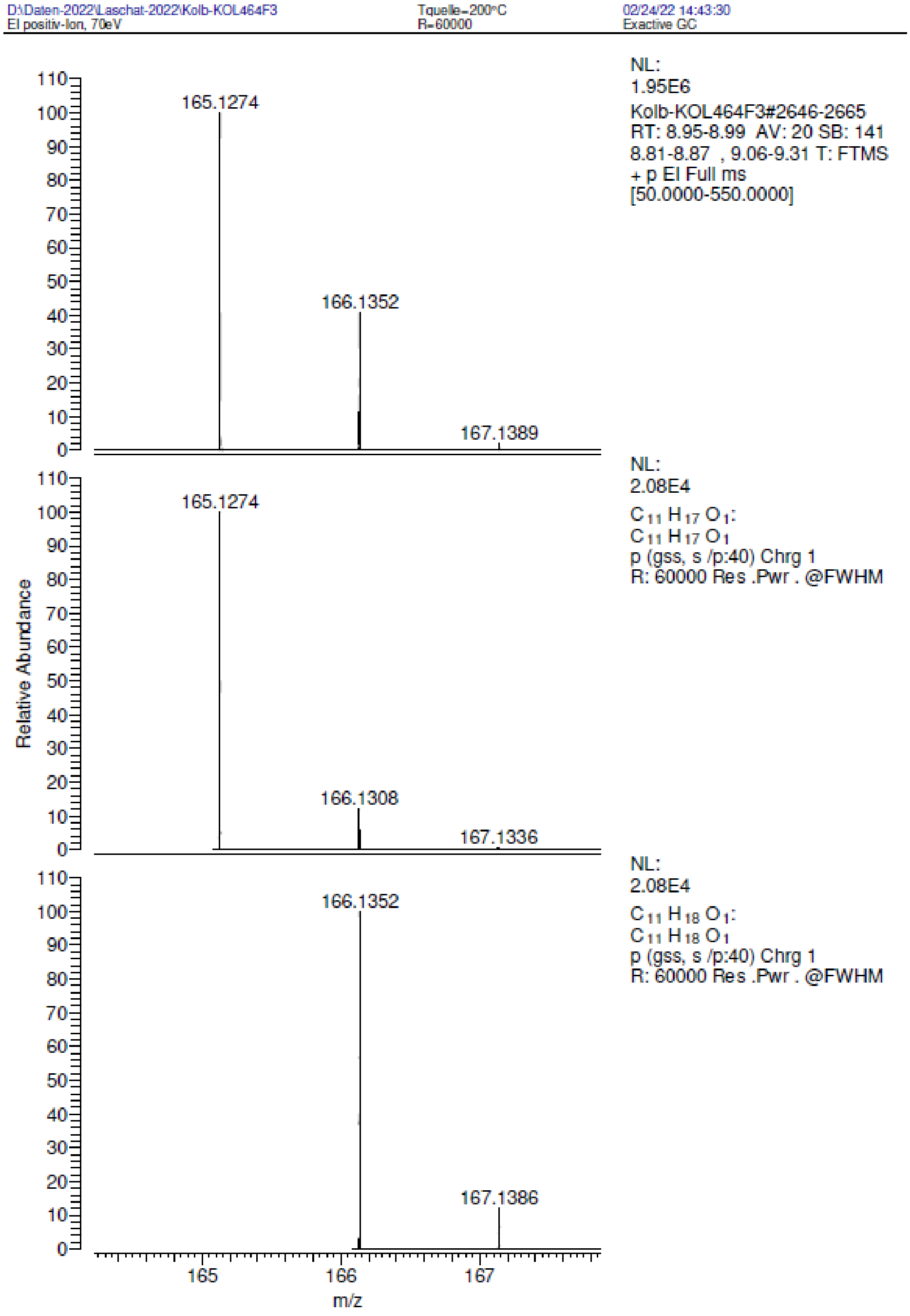

D:\Daten-2022\Laschat-2022\Kolb-KOL464F3
EI positiv-Ion, 70eV
Tquelle=200°C
R=60000
02/24/22 14:43:30
Exactive GC
NL:
1.95E6
Kolb-KOL464F3#2646-2665
RT: 8.95-8.99 AV: 20 SB: 141
8.81-8.87 , 9.06-9.31 T: FTMS
+ p EI Full ms
[50.0000-550.0000]
165.1274
166.1352
167.1389
NL:
2.08E4
C 11 H 17 O 1:
C 11 H 17 O 1
p (gss, s /p:40) Chrg 1
R: 60000 Res .Pwr . @FWHM
165.1274
166.1308
167.1336
NL:
2.08E4
C 11 H 18 O 1:
C 11 H 18 O 1
p (gss, s /p:40) Chrg 1
R: 60000 Res .Pwr . @FWHM
166.1352
167.1386
Relative Abundance
m/z

2-Ethynyl-1,1,3-trimethylcyclohexane (25q)

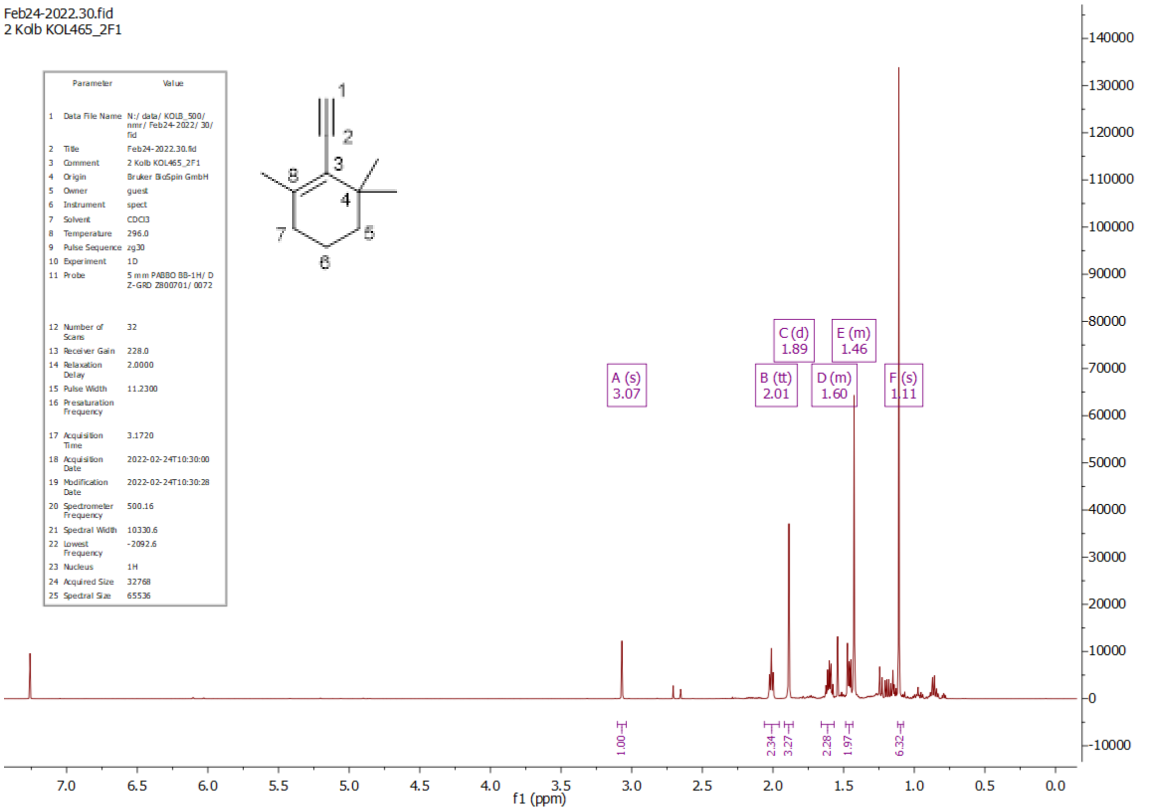

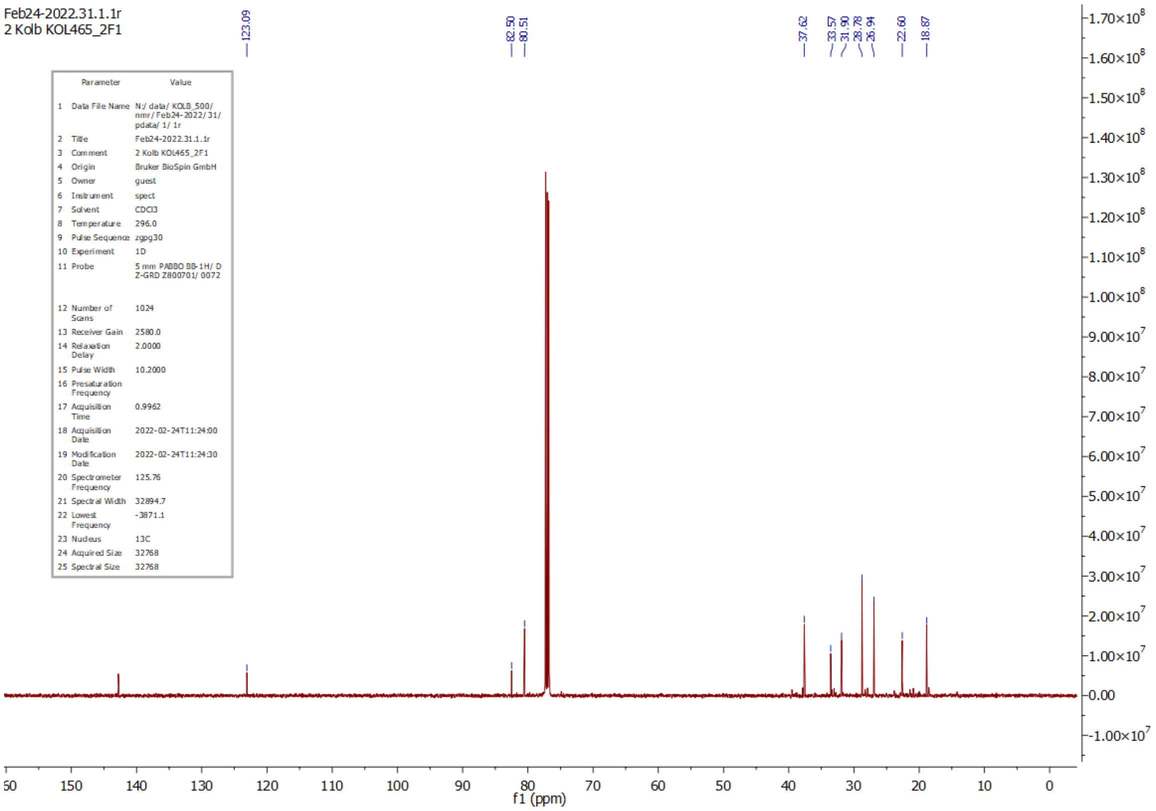

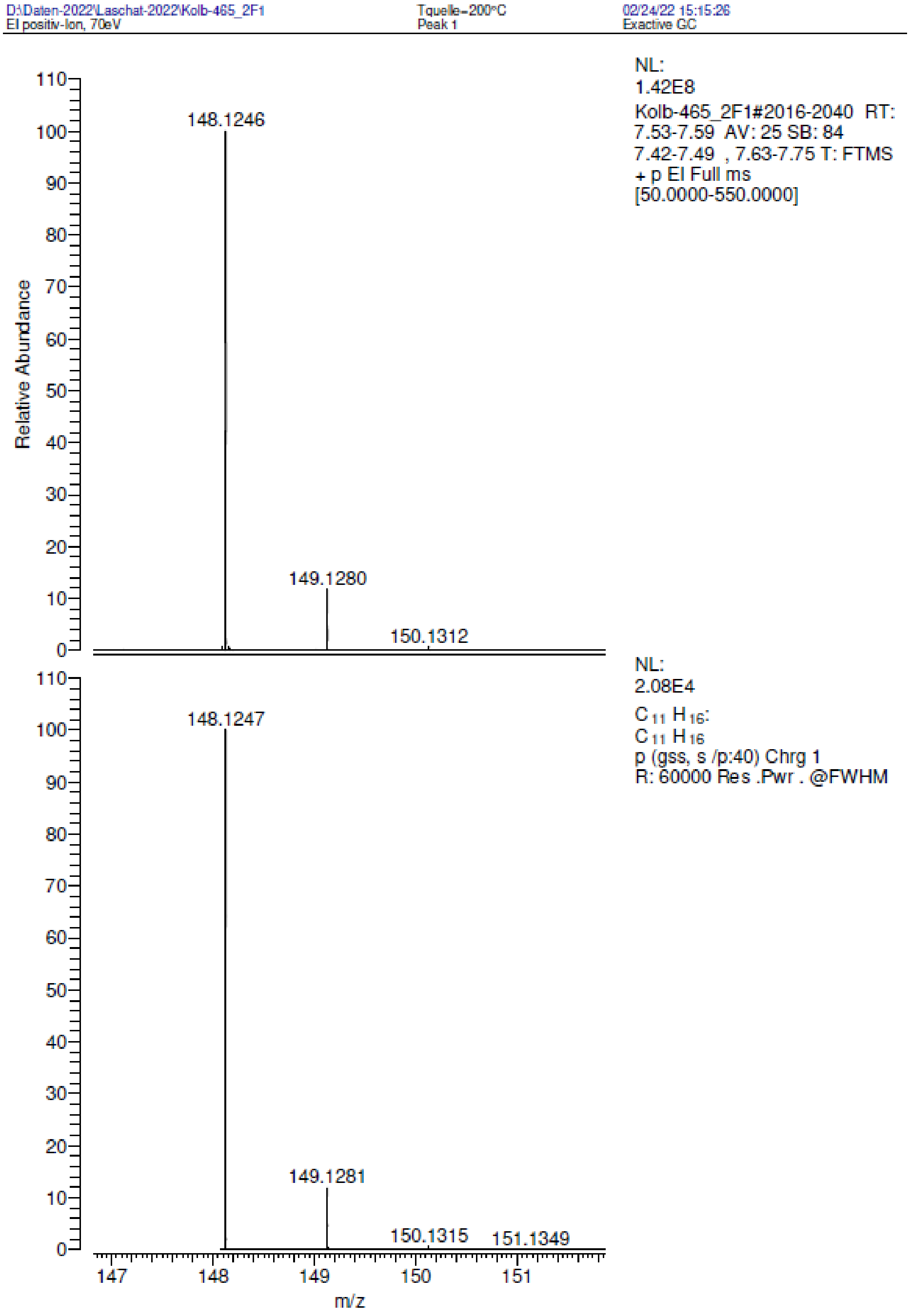
D:\Daten-2022\Laschat-2022\Kolb-465_2F1
EI positiv-Ion, 70eV
Tquelle=200°C
Peak 1
02/24/22 15:15:26
Exactive GC
NL:
1.42E8
Kolb-465_2F1#2016-2040 RT: 7.53-7.59 AV: 25 SB: 84 7.42-7.49 , 7.63-7.75 T: FTMS + p EI Full ms [50.0000-550.0000]
148.1246
149.1280
150.1312
NL:
2.08E4
C 11 H 16:
C 11 H 16
p (gss, s /p:40) Chrg 1
R: 60000 Res .Pwr . @FWHM
148.1247
149.1281
150.1315
151.1349
Relative Abundance
m/z

(*E*)-1-Phenyl-3-(2,6,6-trimethylcyclohex-1-en-1-yl)prop-2-en-1-one (27qa)

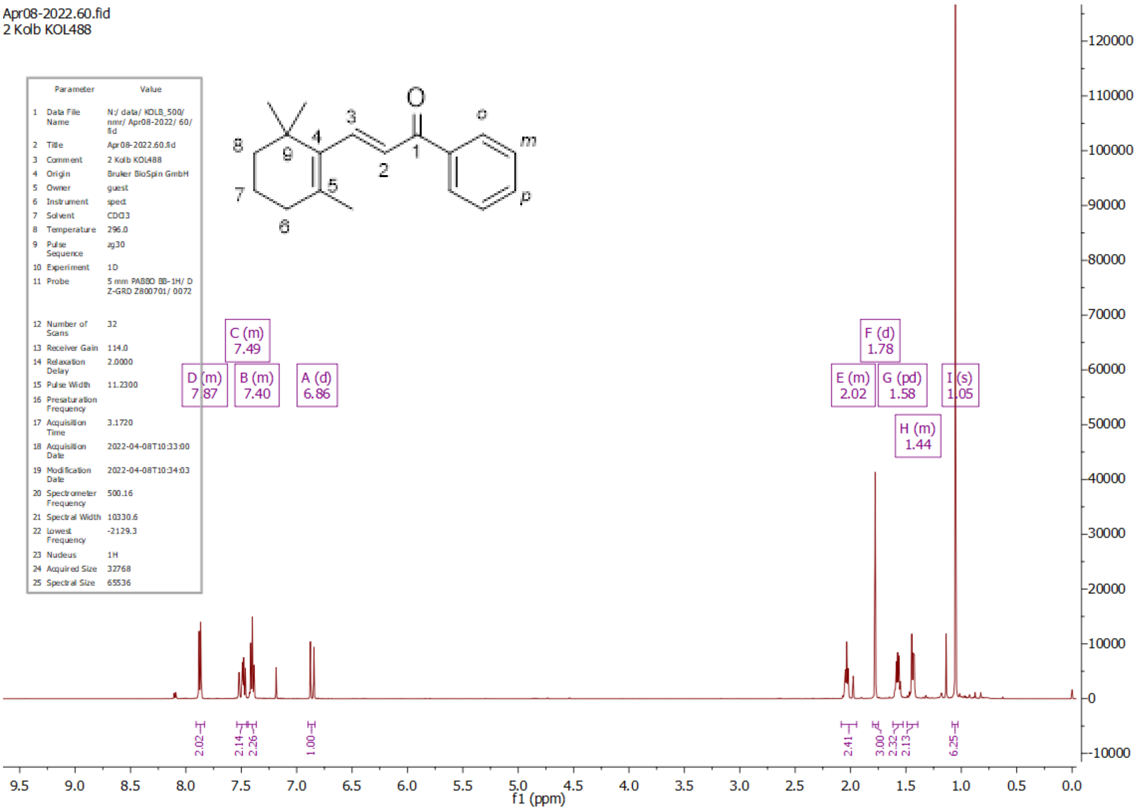

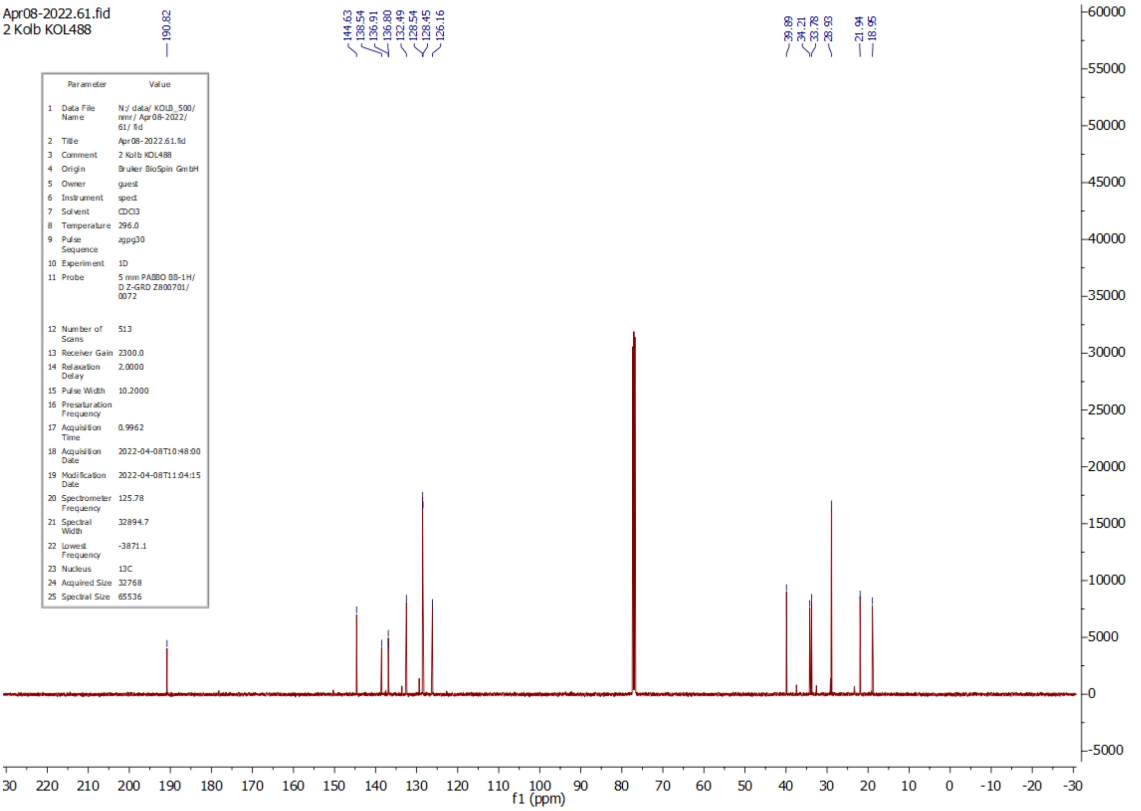

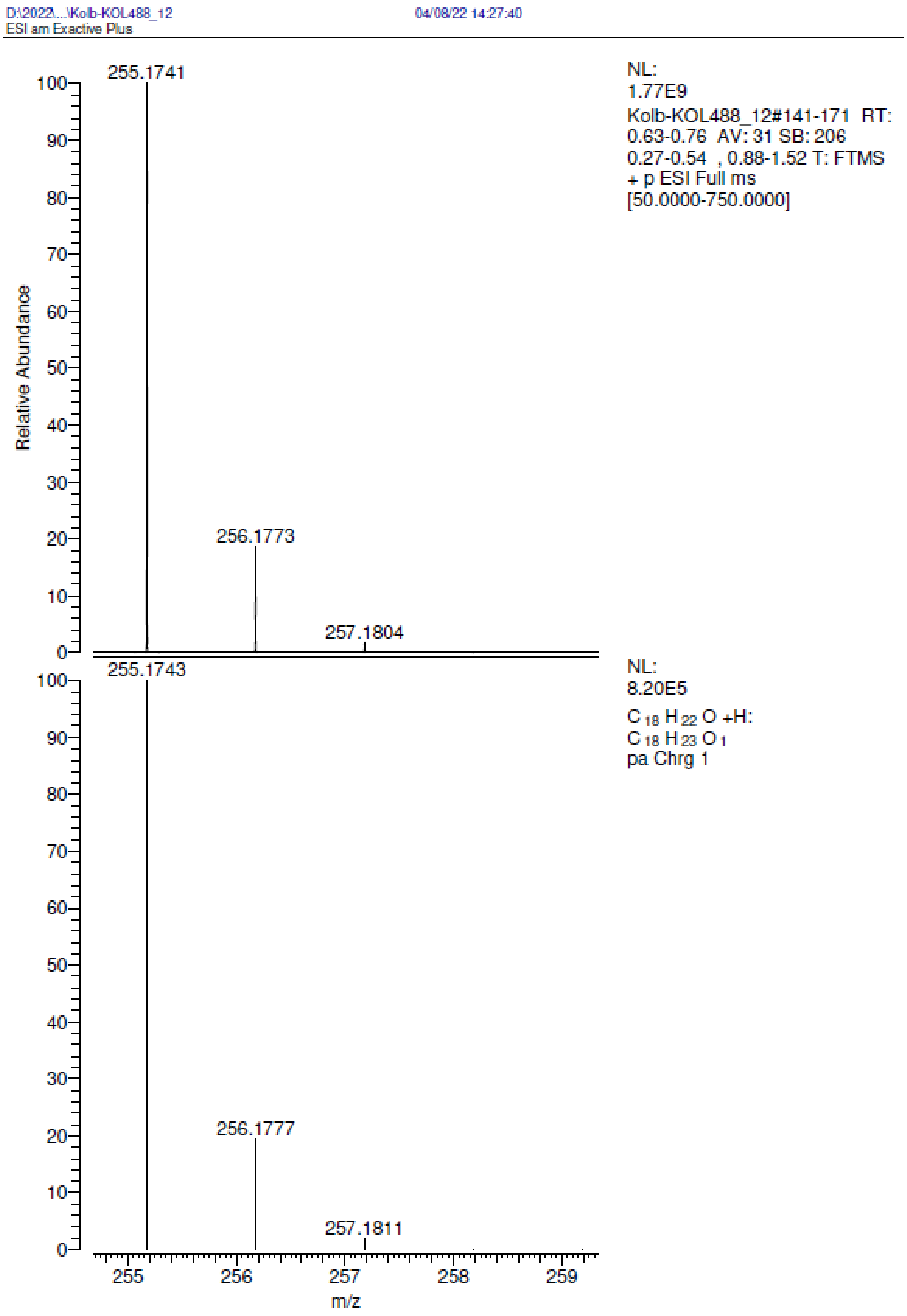

D:\2022\...\Kolb-KOL488_12
ESI am Exactive Plus
04/08/22 14:27:40
NL:
1.77E9
Kolb-KOL488_12#141-171 RT:
0.63-0.76 AV: 31 SB: 206
0.27-0.54 , 0.88-1.52 T: FTMS
+ p ESI Full ms
[50.0000-750.0000]
255.1741
256.1773
257.1804
NL:
8.20E5
C 18 H 22 O +H:
C 18 H 23 O 1
pa Chrg 1
255.1743
256.1777
257.1811
Relative Abundance
m/z

(2*E*,4*E*)-2,4-Dimethyl-1,5-diphenylpenta-2,4-dien-1-one (27ha)

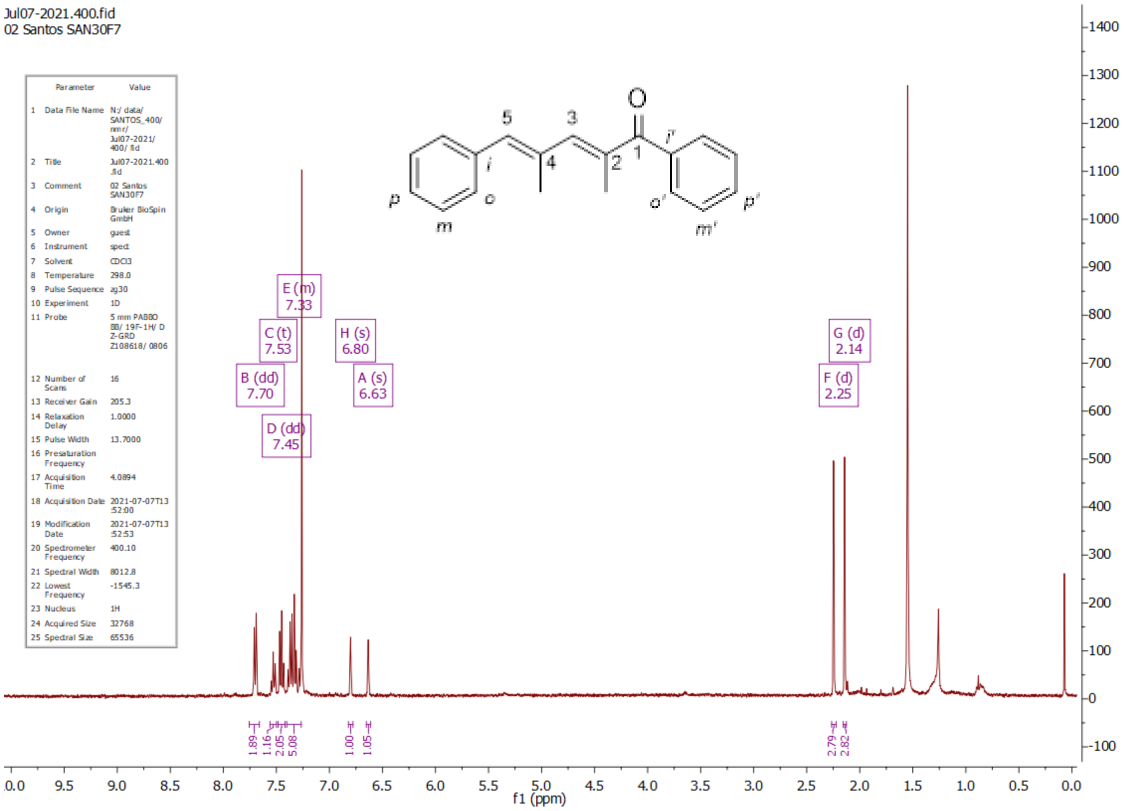

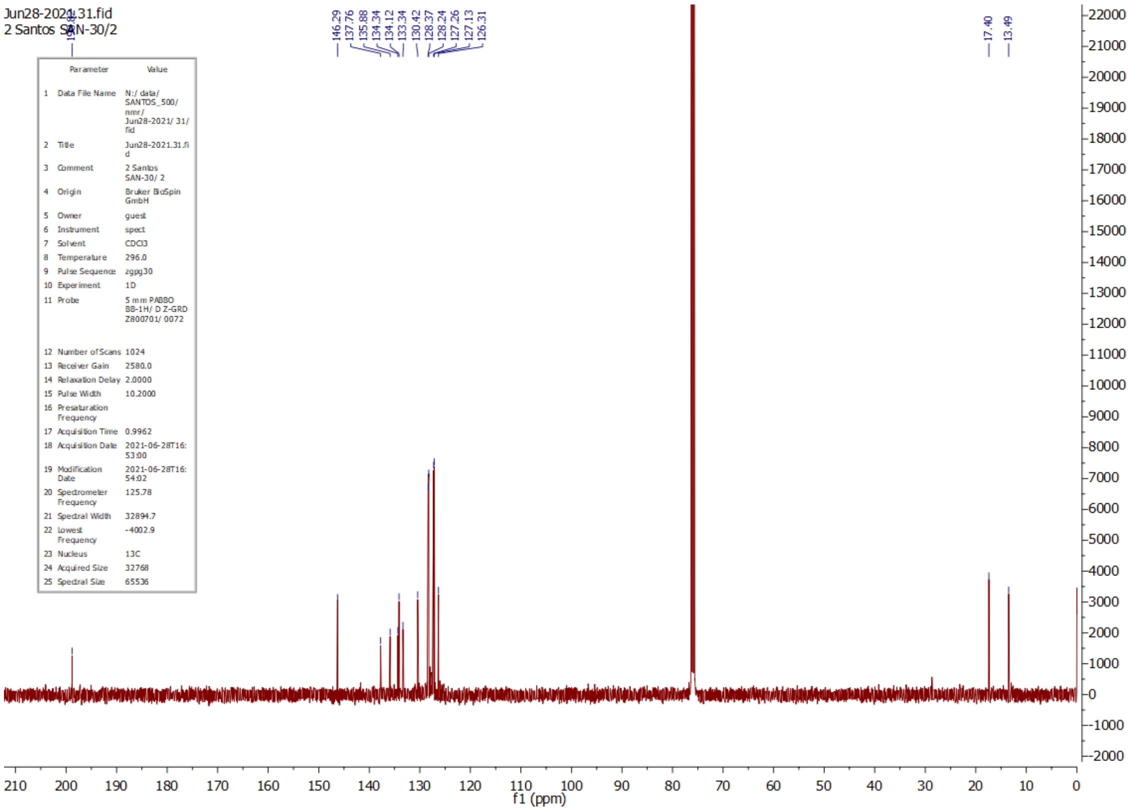

Massenspektrometrie - Universität Stuttgart

Analysis Info

Analysis Name	Santos-SAN-30-2_17_01_32835.d	Acquisition Date	6/29/2021 5:29:52 PM
Method	tune-low-oktober-2019.m	Operator	BDAL@DE
Sample Name	Santos-SAN-30-2	Instrument	micrOTOF-Q 228888.00043
Comment			

Acquisition Parameter

Source Type	ESI	Ion Polarity	Positive	Set Nebulizer	0.4 Bar
Focus	Not active	Set Capillary	4500 V	Set Dry Heater	200 °C
Scan Begin	50 m/z	Set End Plate Offset	-500 V	Set Dry Gas	4.0 l/min
Scan End	1500 m/z	Set Collision Cell RF	180.0 Vpp	Set Divert Valve	Waste

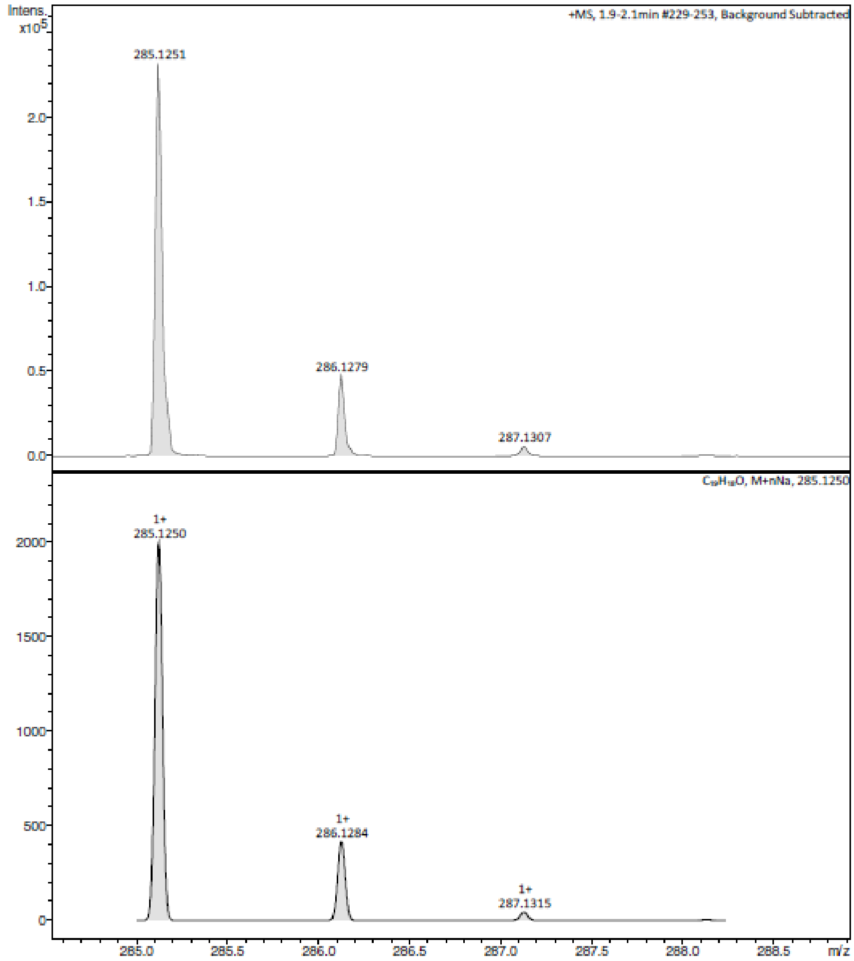

(2*E*,4*E*)-2-Methyl-1-phenyl-5-(*p*-tolyl)penta-2,4-dien-1-one (27oa)

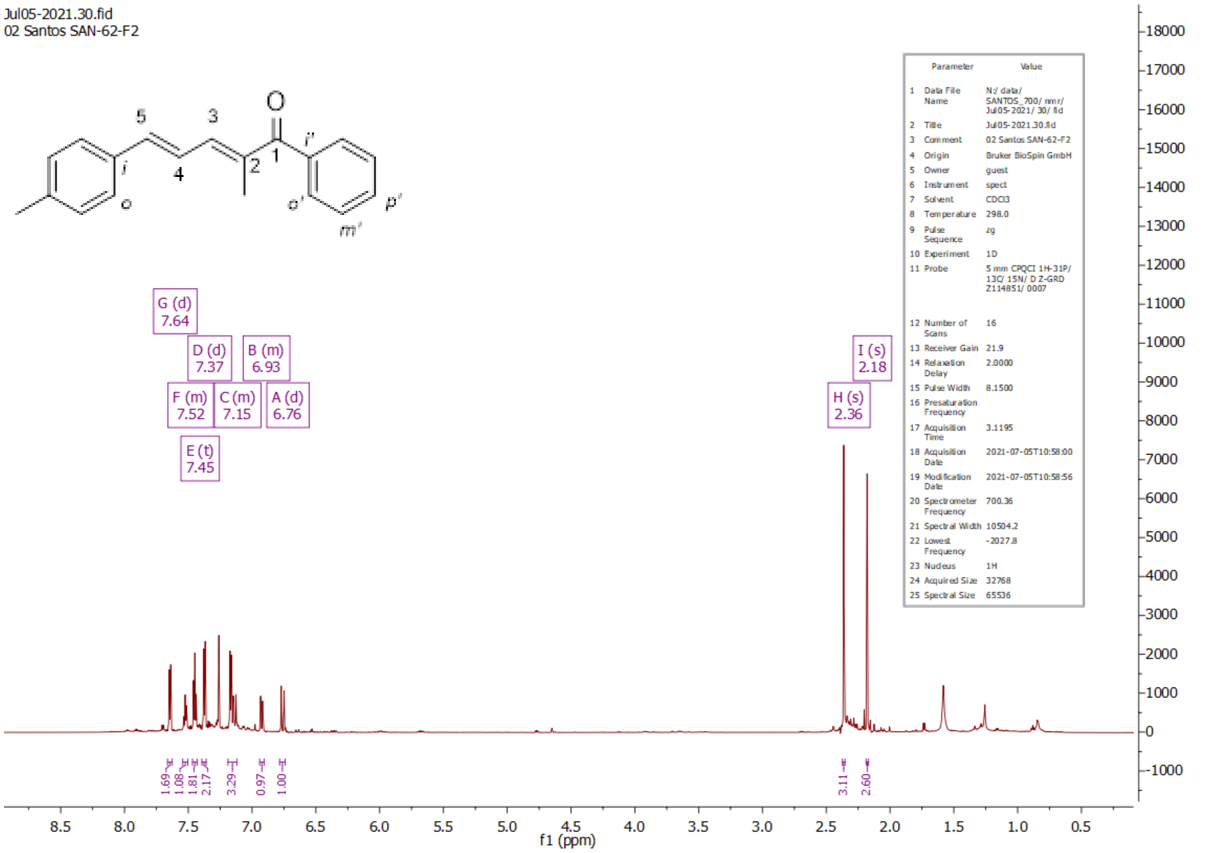

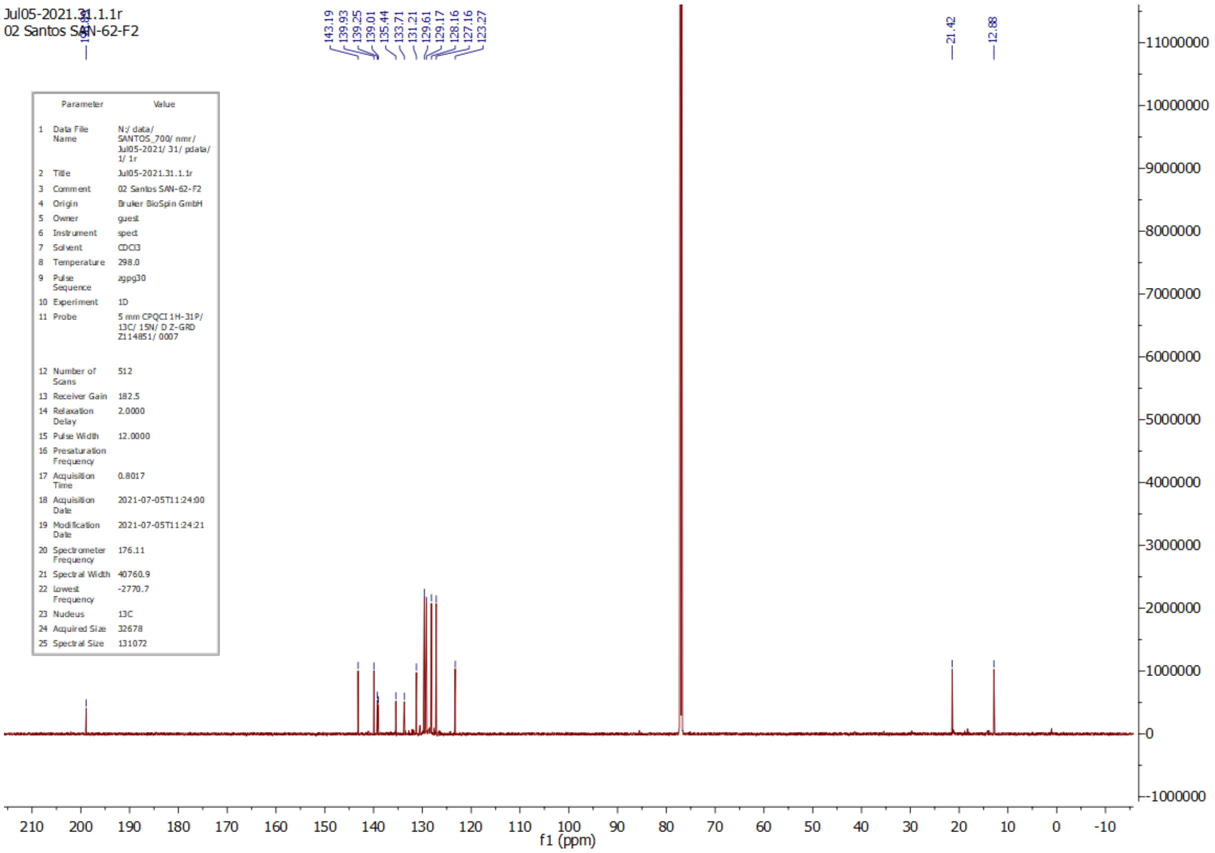

Massenspektrometrie - Universität Stuttgart

Analysis Info

Analysis Name	Santos-SAN-62-F2_1_01_33181.d	Acquisition Date	7/13/2021 7:40:38 AM
Method	tune-low-oktober-2019.m	Operator	BDAL@DE
Sample Name	Santos-SAN-62-F2	Instrument	micrOTOF-Q 228888.00043
Comment			

Acquisition Parameter

Source Type	ESI	Ion Polarity	Positive	Set Nebulizer	0.4 Bar
Focus	Not active	Set Capillary	4500 V	Set Dry Heater	200 °C
Scan Begin	50 m/z	Set End Plate Offset	-500 V	Set Dry Gas	4.0 l/min
Scan End	1500 m/z	Set Collision Cell RF	180.0 Vpp	Set Divert Valve	Waste

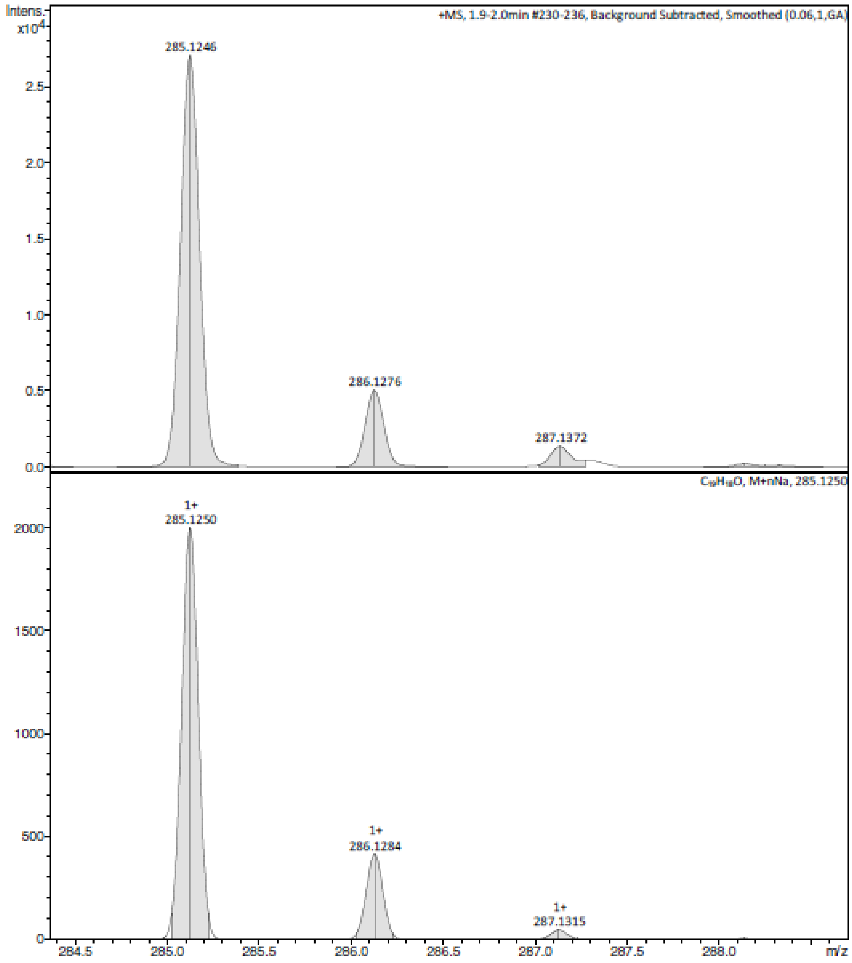

Bruker Compass DataAnalysis 4.2 printed: 7/13/2021 8:16:36 AM Page 1 of 1

(*E*)-(4,4-Dibromo-2-methylbuta-1,3-dien-1-yl)benzene (29f)

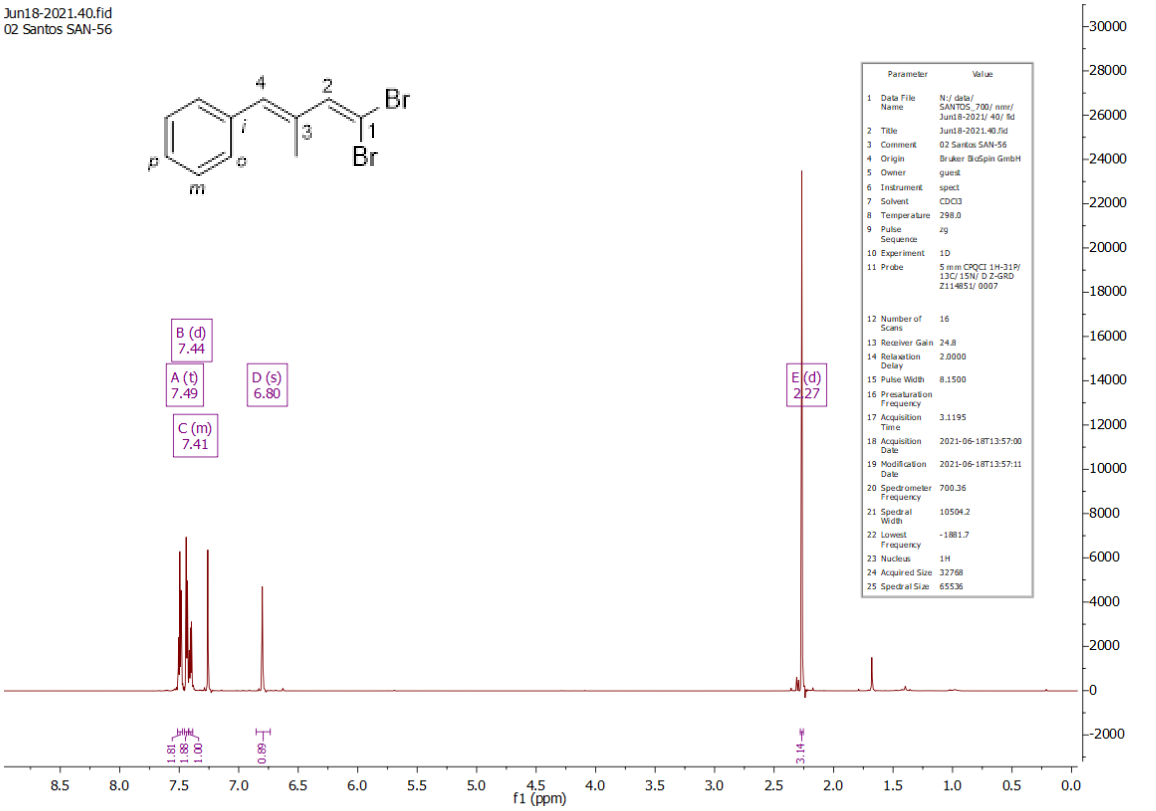

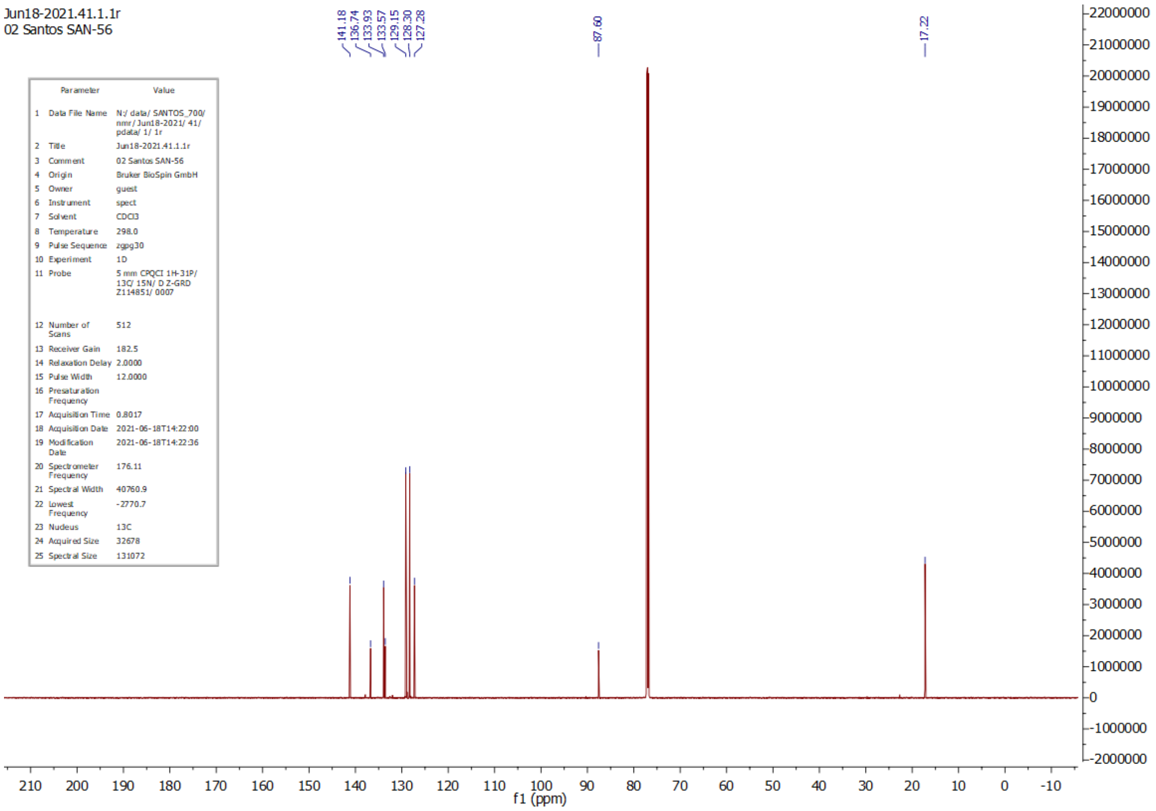

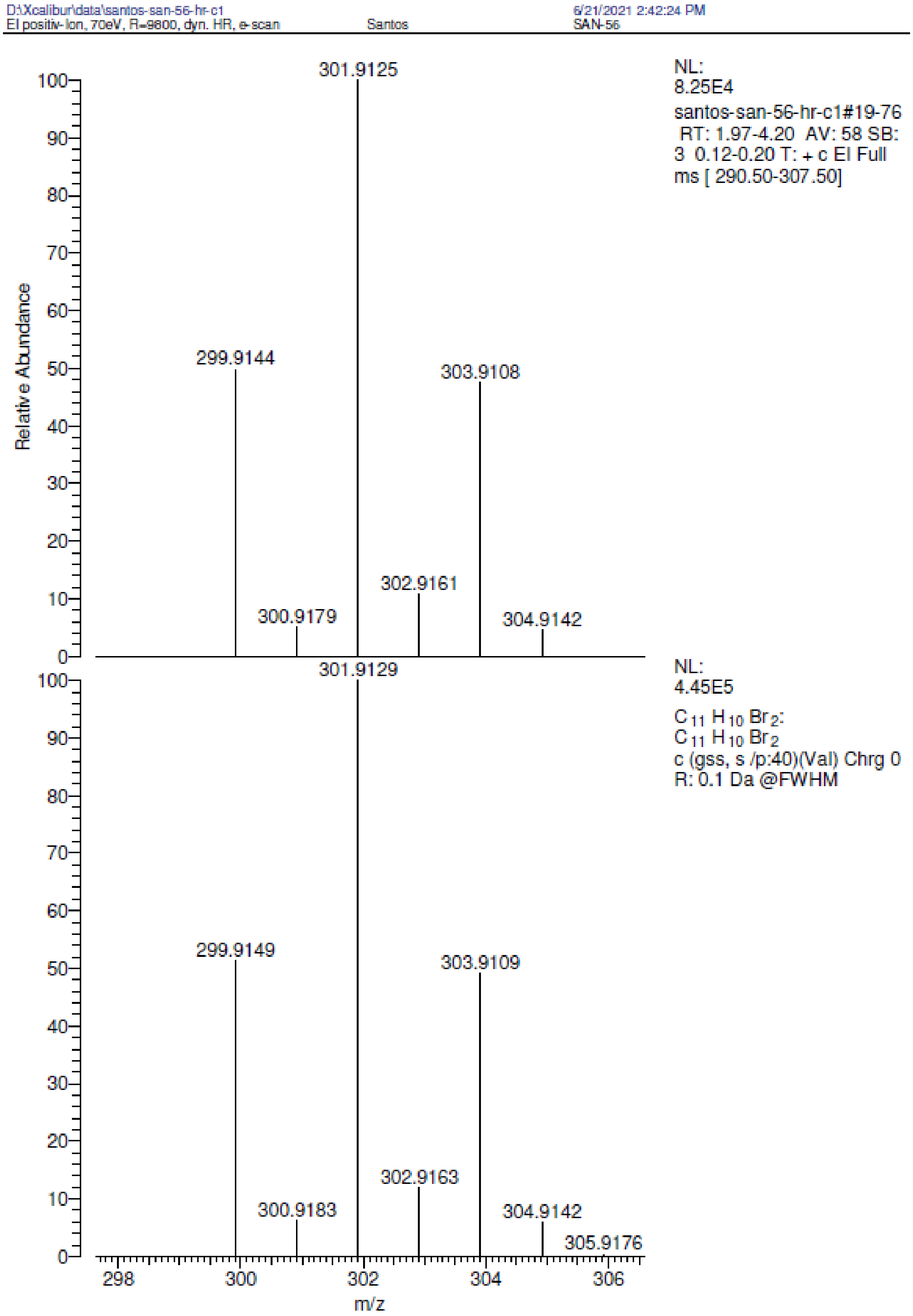
D:\Xcalibur\data\santos-san-56-hr-c1
EI positiv-Ion, 70eV, R=9800, dyn. HR, e-scan
Santos
6/21/2021 2:42:24 PM
SAN-56
NL:
8.25E4
santos-san-56-hr-c1#19-76 RT: 1.97-4.20 AV: 58 SB: 3 0.12-0.20 T: + c EI Full ms [290.50-307.50]
301.9125
299.9144
303.9108
302.9161
300.9179
304.9142
NL:
4.45E5
C 11 H 10 Br 2:
C 11 H 10 Br 2
c (gss, s /p:40)(Val) Chrg 0
R: 0.1 Da @FWHM
301.9129
299.9149
303.9109
302.9163
300.9183
304.9142
305.9176
Relative Abundance
m/z

(*E*)-(2-Methylhex-1-en-3-in-1-yl)benzene (25j)

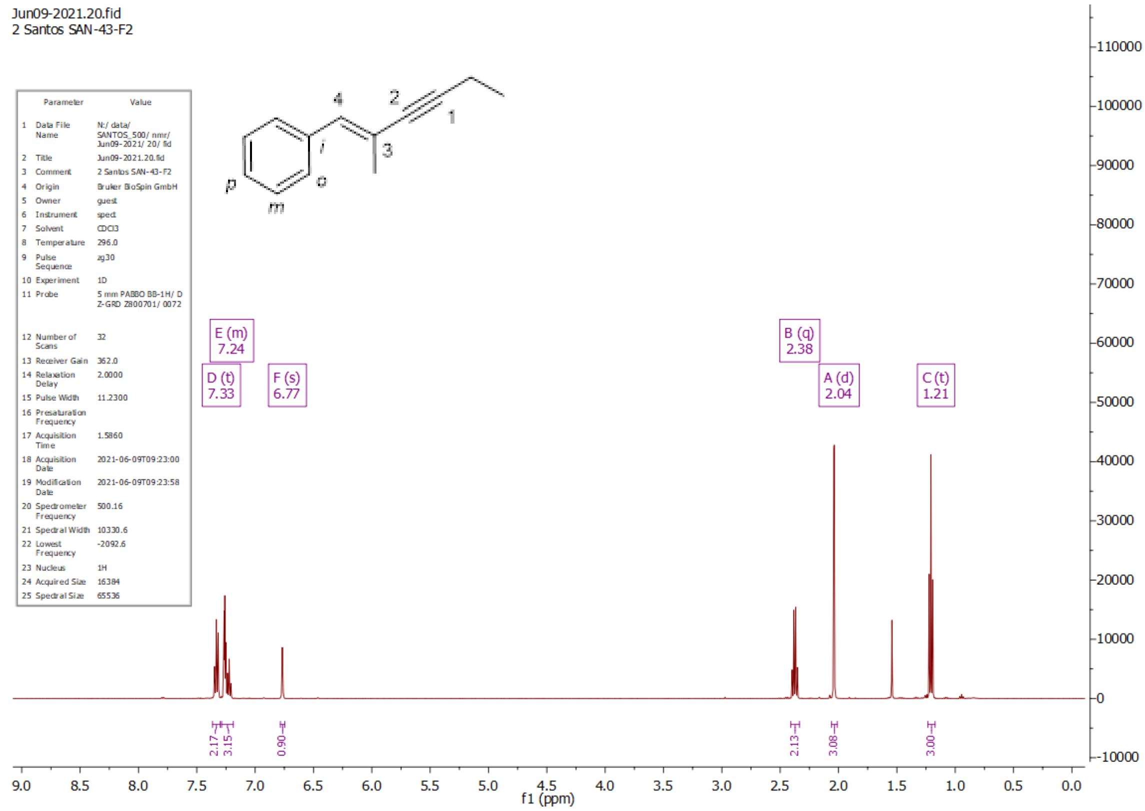

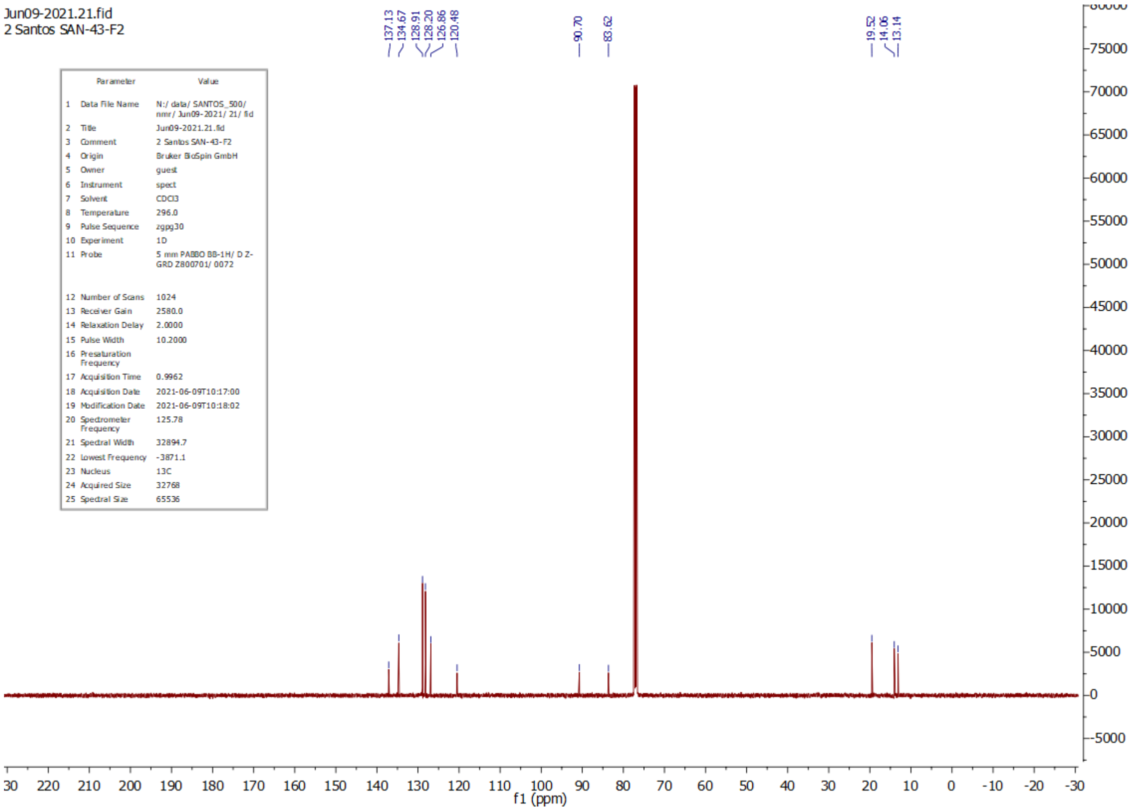

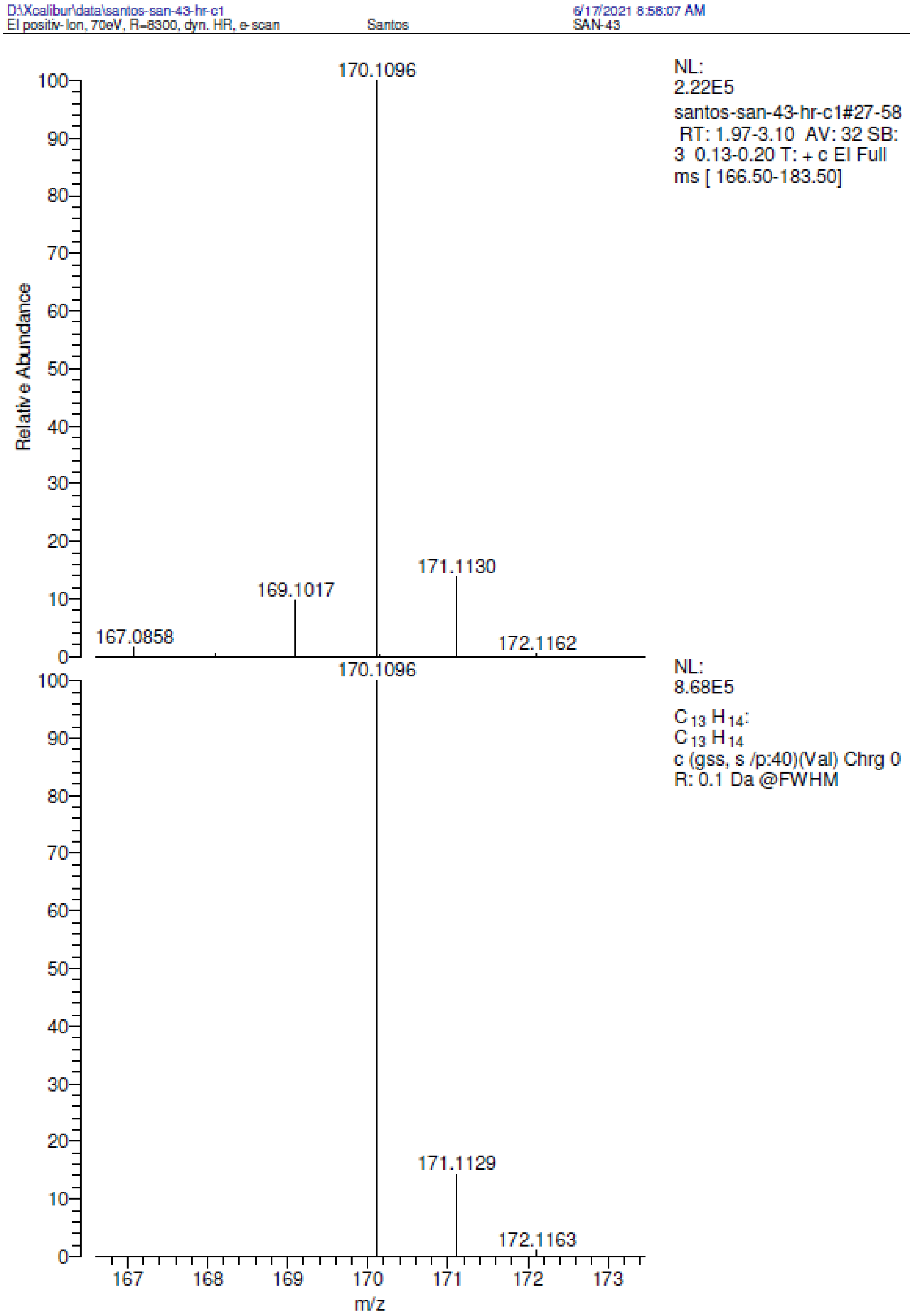
D:\Xcalibur\data\santos-san-43-hr-c1
EI positiv-Ion, 70eV, R~8300, dyn. HR, e-scan
Santos
6/17/2021 8:58:07 AM
SAN-43
NL: 2.22E5
santos-san-43-hr-c1#27-58 RT: 1.97-3.10 AV: 32 SB: 3 0.13-0.20 T: + c EI Full ms [166.50-183.50]
170.1096
171.1130
169.1017
167.0858
172.1162
NL: 8.68E5
C 13 H 14:
C 13 H 14
c (gss, s /p:40)(Val) Chrg 0
R: 0.1 Da @FWHM
170.1096
171.1129
172.1163
Relative Abundance
m/z

(*E*)-(4-Bromo-2-methylbut-1-en-3-in-1-yl)benzene (25n)

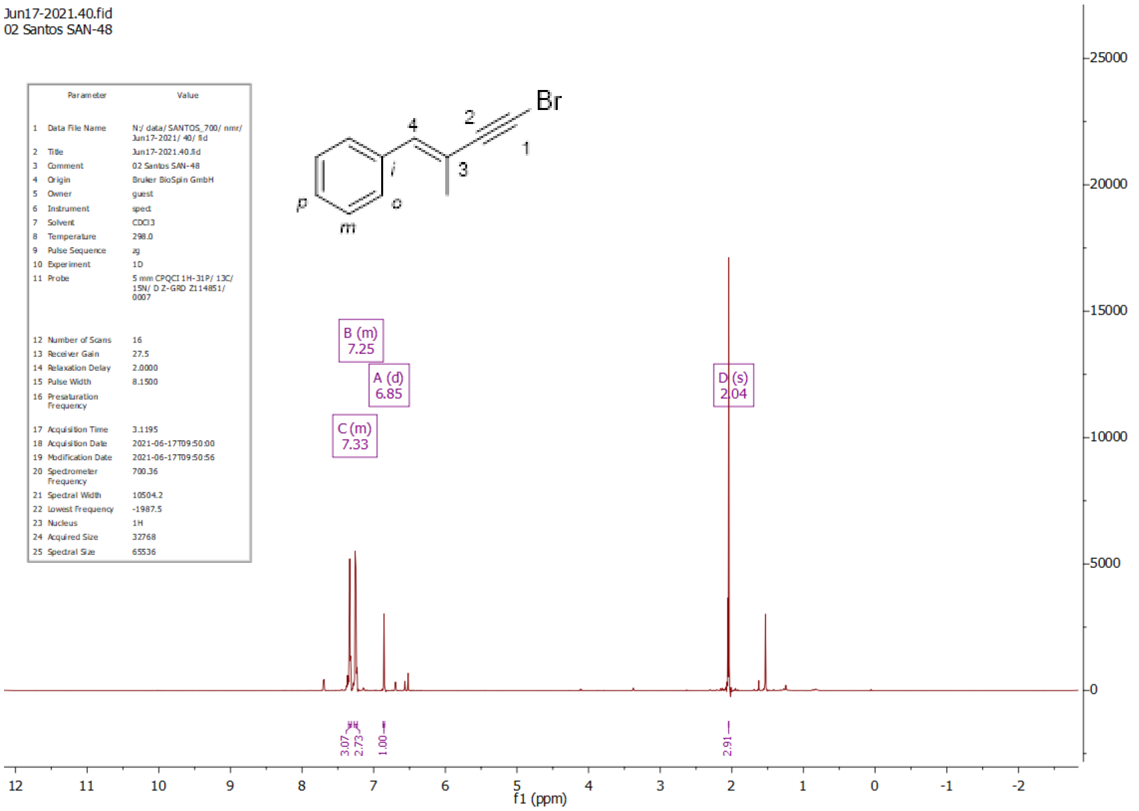

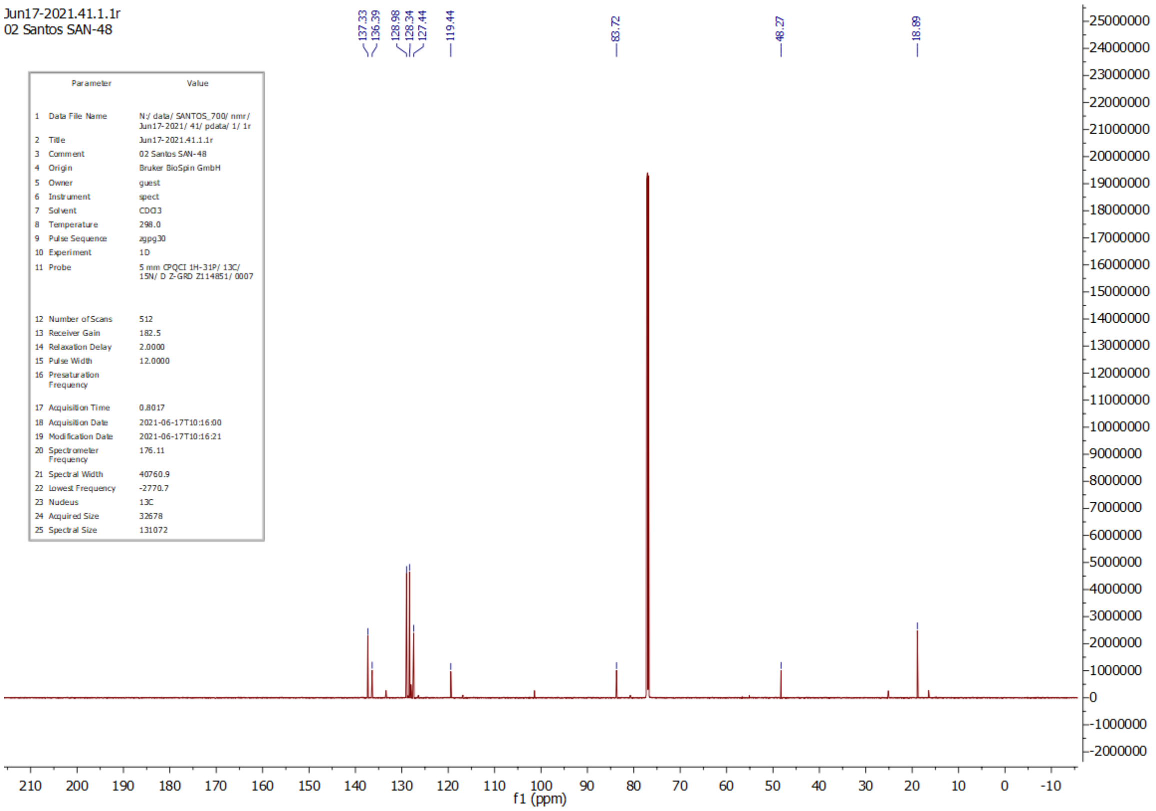

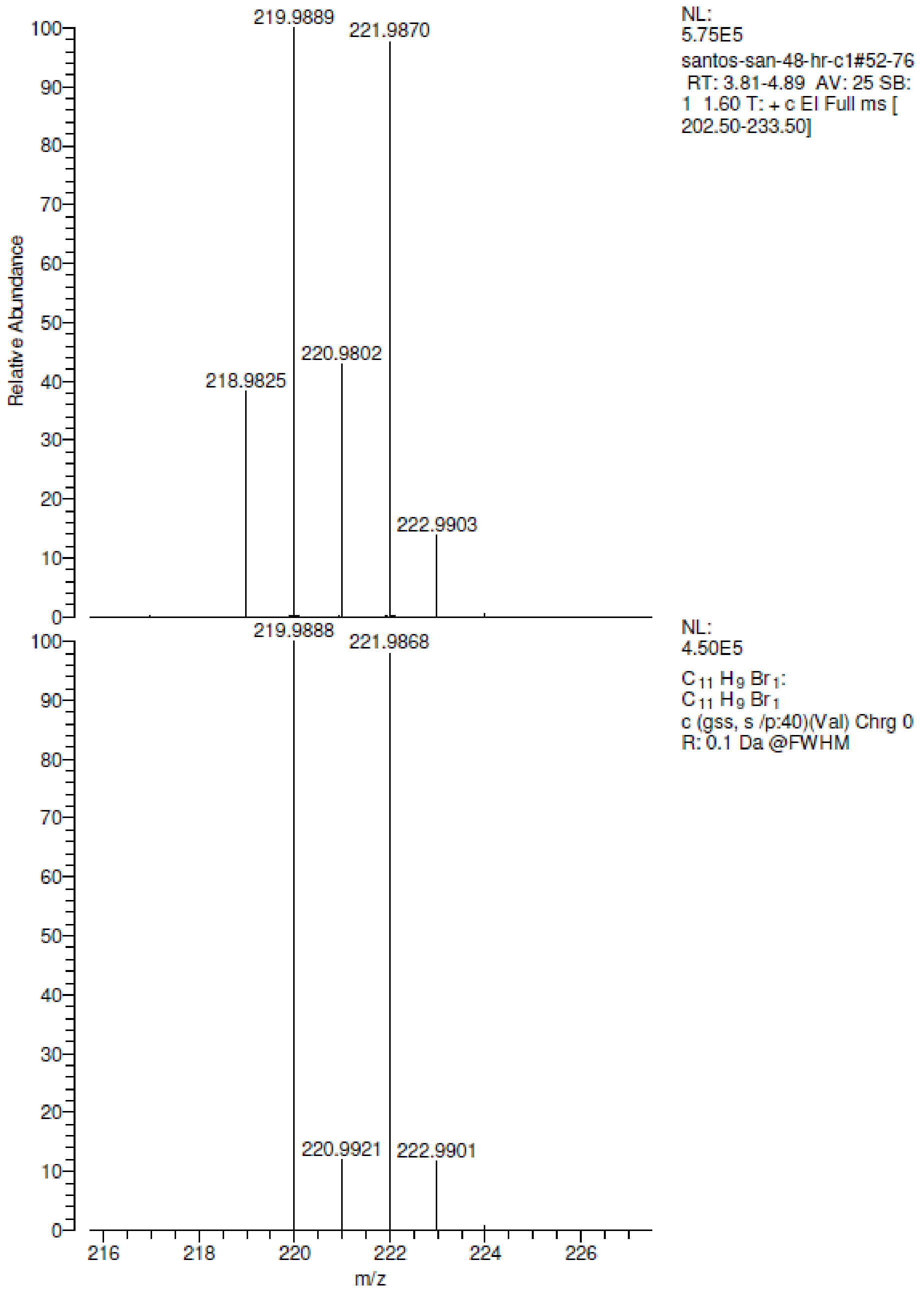
NL:
5.75E5
santos-san-48-hr-c1#52-76 RT: 3.81-4.89 AV: 25 SB: 1 1.60 T: + c EI Full ms [202.50-233.50]
219.9889
221.9870
220.9802
218.9825
222.9903
NL:
4.50E5
C 11 H 9 Br 1:
C 11 H 9 Br 1
c (gss, s /p:40)(Val) Chrg 0
R: 0.1 Da @FWHM
219.9888
221.9868
220.9921
222.9901
Relative Abundance
m/z

4. References

1. Yamamoto, K.; Bruun, T.; Kim, J. Y.; Zhang, L.; Lautens, M. *Org. Lett.* **2016**, *18*, 2644–2647.
2. Birepinte, M.; Liautard, V.; Chabaud, L.; Pucheault, M. *Org. Lett.* **2020**, *22*, 2838–2843.
3. Tian, M.-Q.; Shen, Z.-Y.; Zhao, X.; Walsh, P. J.; Hu, X.-H. *Angew. Chem. Int. Ed.* **2021**, *60*, 9706–9711.
4. Zhao, J.; Zheng, X.; Tao, S.; Zhu, Y.; Yi, J.; Tang, S.; Li, R.; Chen, H.; Fu, H.; Yuan, M. *Org. Lett.* **2021**, *23*, 6067–6072.
5. Yuan, P.; Yin, J.; Yu, G.; Hu, Q.; Liu, S. H. *Organometallics* **2007**, *26*, 196–200.
6. Thummala, Y.; Karunakar, G. V.; Doddi, V. R. *Adv. Synth. Catal.* **2019**, *361*, 611–616.
7. Galler, D. J.; Parker, K. A. *Org. Lett.* **2015**, *17*, 5544–5546
8. Xu, S.; Zhang, Y.; Li, B.; Liu, S.-Y. *J. Am. Chem. Soc.* **2016**, *138*, 14566–14569.
9. Rajmani Singh, R.; Liu, R.-S. *Chem. Comm.* **2014**, *50*, 15864–15866.
10. Westley, E.; Sowden, M. J.; Magann, N. L.; Horvath, K. L.; Connor, K. P. E.; Sherburn, M. S. *J. Am. Chem. Soc.* **2022**, *144*, 977–986.
11. Yang, Z.; Gong, P.-X.; Chen, J.; Zhang, J.; Gong, X.; Han, W. *Synlett* **2021**, *32*, 1207–1212.
12. Yu, B.; Gao, B.; Zhang, X.; Zhang, H.; Huang, H. *Chinese J. Chem*. **2021**, *39*, 566–570.
13. Li, C.; Li, M.; Zhong, W.; Jin, Y.; Li, J.; Wu, W.; Jiang, H. *Org. Lett.* **2019**, *21*, 872–875.
14. Polaquini, C. R.; Torrezan, G. S.; Santos, V. R.; Nazaré, A. C.; Campos, D. L.; Almeida, L. A.; Silva, I. C.; Ferreira, H.; Pavan, F. R.; Duque, C. *Molecules* **2017**, *22*, 1685
15. Santos, C. M. M.; Silva, A. M. S.; Cavaleiro, J. A. S.; Lévai, A.; Patonay, T. *Eur. J. Org. Chem*. **2007**, *2007*, 2877–2887.
16. Armstrong, A.; Pullin, R. D. C.; Jenner, C. R.; Scutt, J. N. *J. Org. Chem.* **2010**, *75*, 3499–3502.
17. Tang, X.-Y.; Shi, M. *Tetrahedron* **2009**, *65*, 8863–8868.
18. Magrez-Chiquet, M.; Morin, M. S. T.; Wencel-Delord, J.; Drissi Amraoui, S.; Baslé, O.; Alexakis, A.; Crévisy, C.; Mauduit, M. *Chem. Eur. J.* **2013**, *19*, 13663–13667.
19. Molander, G. A.; Singaram, B.; Brown, H. C. *J. Org. Chem.* **1984**, *49*, 5024–5025.
20. Peralta-Neel, Z.; Woerpel, K. A. *Org. Lett.* **2021**, *23*, 5002–5006.
21. Bezerra França, S.; Carine Barros de Lima, L.; Rychard da Silva Cunha, C.; Santos Anunciação, D.; Ferreira da Silva-Júnior, E.; Ester de Sá Barreto Barros, M.; José da Paz Lima, D. *Bioorg. Med. Chem.* **2021**, *44*, 116299.
22. Pace, V.; Castoldi, L.; Holzer, W. *J. Org. Chem.* **2013**, *78*, 7764–7770.

23. Dabdoub, M. J.; Silveira, C. C.; Lenardão, E. J.; Guerrero, P. G.; Viana, L. H.; Kawasoko, C. Y.; Baroni, A. C. M. *Tetrahedron Lett.* **2009**, *50*, 5569–5571.

24. Livingstone, K.; Tenberge, M.; Pape, F.; Daniliuc, C. G.; Jamieson, C.; Gilmour, R. *Org. Lett.* **2019**, *21*, 9677–9680.

25. Qian, M.; Huang, Z.; Negishi, E. *Org. Lett.* **2004**, *6*, 1531–1534.
